# Design, Synthesis and Biological Evaluation of Chromeno[3,4‑*b*]xanthones as Multifunctional Agents for Alzheimer’s Disease

**DOI:** 10.1021/acschemneuro.5c00425

**Published:** 2025-08-06

**Authors:** Daniela Malafaia, Natércia F. Brás, Anna Sampietro, Inês Quintelas, Pedro Ferreira, Lúcia Melo, Joana Saavedra, Loreto Martinez-Gonzalez, Marisa Pereira, Jessica Sarabando, Leo König, Isabel Cardoso, Daniela Ribeiro, Ana R. Soares, Raimon Sabaté, Gert Fricker, Ana Martinez, Pedro A. Fernandes, Artur M. S. Silva, Hélio M. T. Albuquerque

**Affiliations:** a LAQV-REQUIMTE, Department of Chemistry, University of Aveiro, 3810-193 Aveiro, Portugal; b LAQV-REQUIMTE, Department of Chemistry and Biochemistry, University of Porto, 4169-007 Porto, Portugal; c Laboratory of Medicinal Chemistry (CSIC Associated Unit), Faculty of Pharmacy and Food Sciences, and Institute of Biomedicine (IBUB), 16724 University of Barcelona, 08007 Barcelona, Spain; d Molecular Neurobiology Group, Institute for Health Research and Innovation (i3S), Institute for Molecular and Cellular Biology (IBMC), 4150-180 Porto, Portugal; e Department of Molecular Biology, Abel Salazar Institute of Biomedical Sciences (ICBAS), University of Porto, 4099-002 Porto, Portugal; f Centro de Investigación Biomédica en Red de Enfermedades Neurodegenerativas (CIBERNED), Instituto de Salud Carlos III, 28040 Madrid, Spain; g Centro de Investigaciones Biologicas, CSIC, Ramiro de Maeztu 9, 28040 Madrid, Spain; h Institute of Biomedicine (iBiMED), Department of Medical Sciences, 56062 University of Aveiro, 3810-193 Aveiro, Portugal; i Ruprecht-Karls Universität Institut für Pharmazie und Molekulare Biotechnologie Im Neuenheimer Feld 329, D-69120 Heidelberg, Germany; j Department of Pharmacy and Pharmaceutical Technology, Department of Physical Chemistry, School of Pharmacy, iUniversity of Barcelona, 08028 Barcelona, Spain; k Institute of Nanoscience and Nanotechnology (IN2UB), University of Barcelona, 08028 Barcelona, Spain; α Centro de Investigaciones Biologicas “Margarita Salas” (CIB-CSIC), Ramiro de Maeztu 9, Madrid 28040, Spain

**Keywords:** Alzheimer’s disease, multifunctional compounds, chromeno[3,4-*b*]xanthone cholinesterases, Aβ aggregation, tau protein aggregation, blood-brain barrier permeability

## Abstract

Alzheimer’s disease (AD) remains a complex and unmet medical challenge, requiring innovative approaches to address its multifaceted pathology. In this study, we explored chromeno­[3,4-*b*]­xanthones as a novel multifunctional scaffold, synthesized via the straightforward cyclization of their precursor, (*E*)-2-styrylchromones. Compounds **10** and **11q**–**s** exhibited potent and selective cholinesterase inhibition (IC_50_ 1.7–9.0 μM for AChE and BChE), along with significant antiamyloid activity (inhibition exceeding 50% at 50 μM). Among them, compound **11r** demonstrated the most well-balanced multifunctional profile against all four AD-relevant targets. Molecular docking studies revealed key π-stacking, hydrogen bonding, and halogen interactions, which underlie the selective binding of compound **11r** to AChE and BChE. Moreover, docking and molecular dynamics simulations showed that compound **11r** binds strongly to the L-S-shaped β-amyloid 1–42 (Aβ_42_) fibril with a binding affinity of −11.3 kcal/mol, representing a structural barrier to Aβ_42_ elongation. Additionally, compound **11r**, selected as the representative scaffold, effectively disrupted Aβ aggregation, as demonstrated by *in vitro* studies, transmission electron microscopy (TEM), and cellular studies. It also displayed favorable drug-like properties, including predicted blood-brain barrier (BBB) permeability and an acceptable safety profile at active doses. The calcein-AM-assay also showed that this compound is unlikely to be actively effluxed from the brain. These findings underscore the therapeutic potential of chromeno­[3,4-*b*]­xanthone as multifunctional agents for AD, broadening the chemical space of small-molecule exploration.

## Graphical abstract

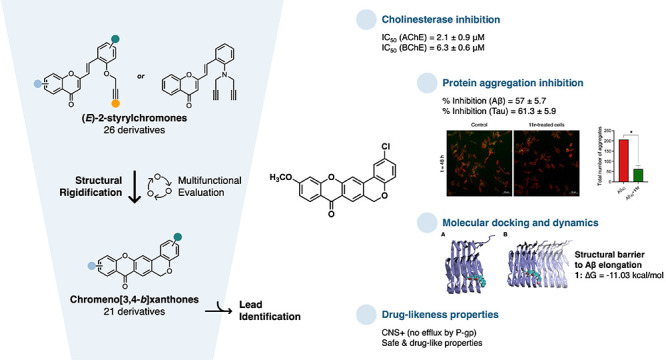

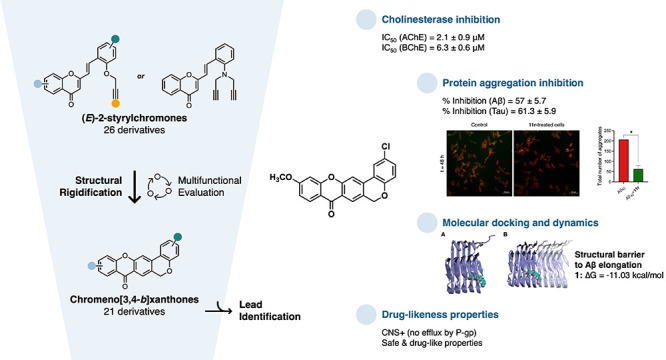

## Introduction

Alzheimer’s disease (AD) is the most common neurodegenerative disorder, characterized by the progressive decline of the cognitive, motor, and functional capacities, ultimately leading to dementia.^1,2^ This complex disease has been progressively growing as the world’s population ages, currently affecting an estimated 50 million people worldwide, number that is projected to reach 150 million by 2050.^3–5^ Alongside the demographic challenge posed by the increasing global prevalence and mortality of AD, is the low success rate in the development of new disease-modifying therapies (DMTs).^4,6^ The current therapeutic approach for AD is predominantly based on four pharmacological agents, responsible for the modulation of the cholinergic (donepezil, rivastigmine and galantamine)^7–9^ and glutamatergic (memantine)^10^ neurotransmission systems, that only alleviate the symptoms and do not alter the underlying disease pathology.^4,11,12^ Since the approval of these pharmacological agents in the early 2000s, only a limited number of new drugs were permitted for the treatment of AD, including sodium oligomannate GV-971 (only approved in China, in 2019)^13^ and the monoclonal antibodies aducanumab,^14^ lecanemab^15^ and donanemab (currently under review for market approval).^16^ Despite the controversy concerning the approval by the Food and Drug Administration (FDA), the monoclonal antibodies were a landmark in AD drug discovery and the resurgence of a critical hypothesis related to the onset of the disease, the β-amyloid (Aβ) aggregation process. Nevertheless, given the high cost associated with this type of therapy (estimated at up to $26.500 per patient per year), most experts anticipate that access to it will be highly restricted, particularly in low- and middle-income countries, with limited healthcare resources.^17^ For this reason, there has been a need for a re-examination of the drug development process, focused on the disease-modifying pharmacological targets and on patient compliance.^5^ Small molecules offer a promising alternative for overcoming these challenges by enabling earlier initiation of treatment regimens and improving patient adherence over extended periods. They are relatively less complex structures, typically more cost-effective and suitable for at-home consumption, as they provide versatile administration options, including pills, inhalers, suppositories or injectables.^18^ Their structural simplicity allows for customization to meet specific therapeutic objectives, as they can be engineered to interact selectively with particular biological targets or structurally modified to precisely tune their properties to achieve targeted therapeutic outcomes.^18^ Over the last year, around 187 trials have been conducted for 141 distinct treatments for AD, of which 111 were focused on DMTs and more than half involve small-molecule therapeutic agents.^6^ However, previous small-molecule candidates have been hindered by limited efficacy, lack of novelty and insufficient structural diversity.^19^ Classical drug discovery follows the “one disease, one target, one drug” paradigm. While this strategy has led to the development of numerous successful therapies, it proves less effective for diseases such as AD, which are characterized by multiple contributing factors, each partially influencing the disease’s underlying pathophysiology.^4^ Consequently, a shift toward “polypharmacology” using multitarget directed ligands (MTDLs) has been gaining attraction since the beginning of the millennium, offering a particularly promising approach for treating complex and multifactorial diseases.^20^ Under optimal conditions, the MTDL approach is expected to provide more advantages compared to combination therapy, such as a superior pharmacokinetic and safety profile (since the risk of adverse effects increases with the number of combined drugs), reduced risk of acquired resistance, and a simplified formulation that may enhance patient compliance.^20,21^ The development of MTDLs begins with the careful selection of suitable targets, a critical and complex step in the process. Not all target combinations produce synergetic effects, nor are all combinations amenable to being addressed simultaneously by a single molecular entity.^20^ For this reason, many experts argue that multitarget drugs should be designed using a more rational and comprehensive approach to target combinations, as current strategies often rely on the simplistic method of linking, fusing or merging two or more active ligands together, usually derived from existing drugs (selected examples depicted in Figure 1).^22–24^

**Figure 1:**
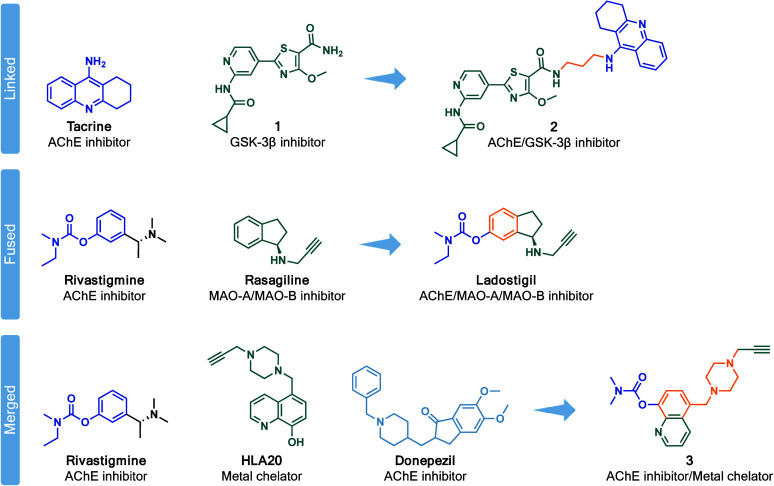
Examples of promising MTDLs for AD based on the framework combination strategy: linkage, fusion, and merging.

Another critical consideration in developing MTDLs is that, to achieve synergy, they must aim at the subpathologies of AD that occur simultaneously across its progression.^20^ Based on this, we can categorize the stages of intervention into three phases: (i) primary prevention, which targets subpathologies before any signs or biomarkers of AD appear, such as therapies addressing metabolic risk factors; (ii) secondary prevention, focused on preventing cognitive decline by targeting early stage subpathologies like Aβ and tau protein aggregation; and (iii) symptomatic treatment, which targets the later stages of the disease, aiming to manage cognitive deterioration through therapies that address neurotransmission, such as the currently available drugs (Figure 2).

**Figure 2:**
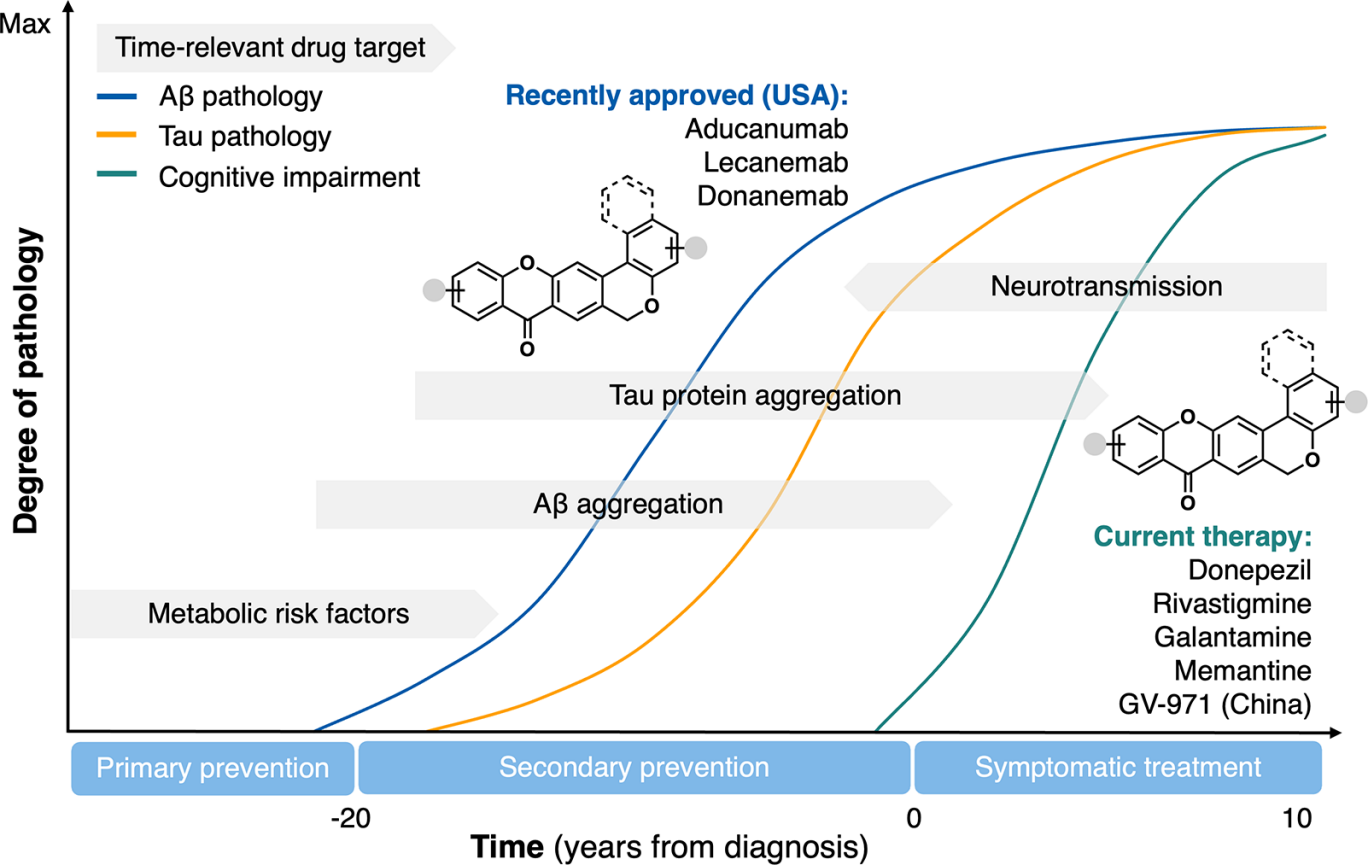
Graphical representation of a simplified hypothetical model illustrating the major hallmarks of AD, emphasizing the relevance of therapeutic interventions to its progression, alongside the potential therapeutic application of the multifunctional chromeno­[3,4-*b*]­xanthone scaffold.

Recently, we disclosed a novel scaffold, i.e., chromeno­[3,4-*b*]­xanthones,^25^ which incorporate structural features of both chromone^26,27^ and xanthone-based^28^ pharmacophores, two privileged scaffolds in medicinal chemistry, particularly in neurodegenerative disorder research (Figure 3). Synthetically derived chromone-based compounds, such as 2-phenylchromones and chromone-aminoalkyl hybrids, have demonstrated potent inhibition of cholinesterases (AChE and BChE), monoamine oxidases (MAO-A and MAO-B), and Aβ aggregation, as well as antioxidant activity and predicted blood-brain barrier (BBB) permeability (Figure 3A).^29,30^ Similarly, synthetic xanthone derivatives, including aminoalkoxy-substituted xanthones, have also shown multifunctional activity, with some compounds displaying dual AChE/MAO-B inhibition, significant antioxidant effects and neuroprotective properties both *in vitro* and *in vivo* (Figure 3A).^31–33^ The chromeno­[3,4-*b*]­xanthone scaffold, centered on extended aromaticity and planarity, features commonly found in amyloid and tau aggregation inhibitors as well as in cholinesterase ligands such as tacrine or its analogues,^34–38^ thus integrates these two pharmacologically relevant structural motifs into a single, synthetically accessible framework (Figure 3B). Although the development of these compounds deviates from the conventional strategies of MTDLs, both in terms of design and synthesis, it remains notably straightforward. This simplicity is achieved through the cyclization of (*E*)-2-styrylchromones, which retain the chromone pharmacophore, while exhibiting structural flexibility (Figure 3B). The aromatic nature of the chromeno­[3,4-*b*]­xanthone scaffold coupled with strategically positioned hydrogen bonding sites enhances the binding affinity and biological activity against the targets and contributes to the lipophilicity and, subsequently, BBB permeability of the compounds, one of the major challenges in central nervous system (CNS) drug discovery (Figure 3B). The preliminary biological evaluation of these compounds revealed a well-balanced inhibitory profile against acetylcholinesterase (AChE) and Aβ aggregation at low micromolar concentrations, with some compounds exhibiting inhibition percentages exceeding 50% for both targets at a screening concentration of 20 μM.^25^ Building on these promising results and to further explore their therapeutic potential, we expanded the chromeno­[3,4-*b*]­xanthone scaffold by synthesizing and evaluating structurally related derivatives designed to address multiple pathological features of AD (Figure 2 and 3). Unlike traditional MTDLs, which typically aim to address subpathologies that occur simultaneously, our approach introduces a molecule that can target distinct subpathologies at different stages of the disease. Specifically, it can address secondary prevention early in the disease progression, while also providing symptomatic relief as the disease advances (Figure 2). This flexibility offers a more personalized therapeutic approach, catering to the dynamic needs of the patient as their condition evolves.

**Figure 3:**
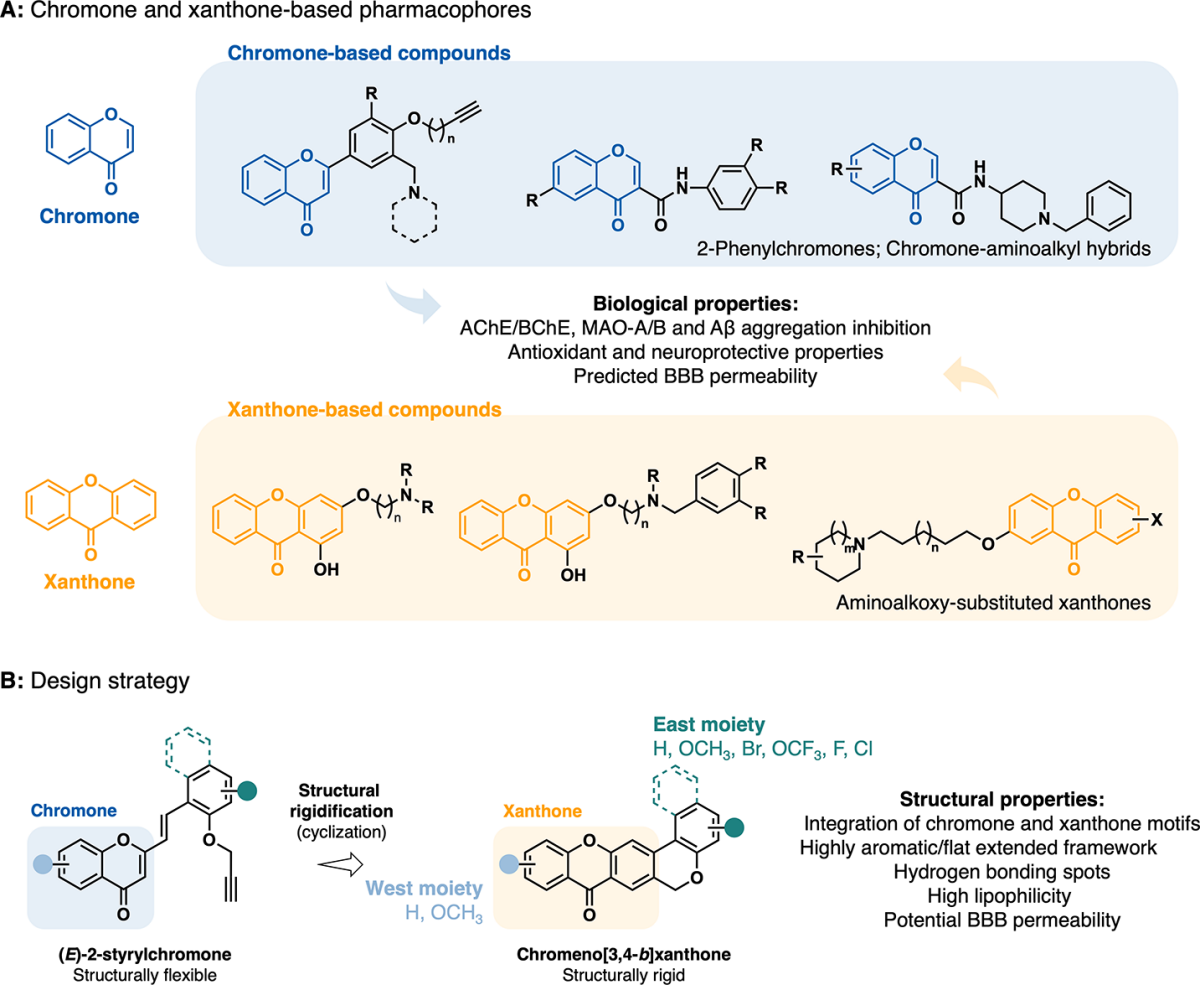
Chromone and xanthone-based pharmacophores and design strategy of the chromeno­[3,4-*b*]­xanthone scaffold. (A) Representative chromone and xanthone derivatives with noted bioactivities related to AD. (B) Design strategy based on the structural rigidification of (*E*)-2-styrylchromone into the chromeno­[3,4-*b*]­xanthone scaffold, highlighting key structural properties.

Therefore, herein we present our extended study on the synthesis of a larger compound library and biological evaluation of chromeno­[3,4-*b*]­xanthones and their structurally flexible precursors (*E*)-2-styrylchromones, profiling a new multifunctional small-molecule for AD.

## Results and Discussion

### Chemistry

Compounds **7a**–**w**, **8a**–**b**, **10**, **11a**–**s** and **12a**–**b** were synthesized following a convergent approach as depicted in Schemes 1–3, in which previously prepared 2-methylchromones and appropriately substituted salicylaldehydes converge toward the desired final compounds. The first step of this strategy involves a base-promoted aldol condensation of 2-methylchromones **4** with aldehydes **5**, **6** or **9**, giving (*E*)-2-styrylchromones **7a-w, 8a-b** and **10** in 20–91%, 32–64% and 82% yields, respectively (Schemes 1 and 2). The 2-methylchromones **4**, along with aldehydes **5**, **6** and **9**, are not commercially available and were synthesized according to previously reported methodologies (see Supporting Information, Schemes S1–S4).

**Scheme 1:**
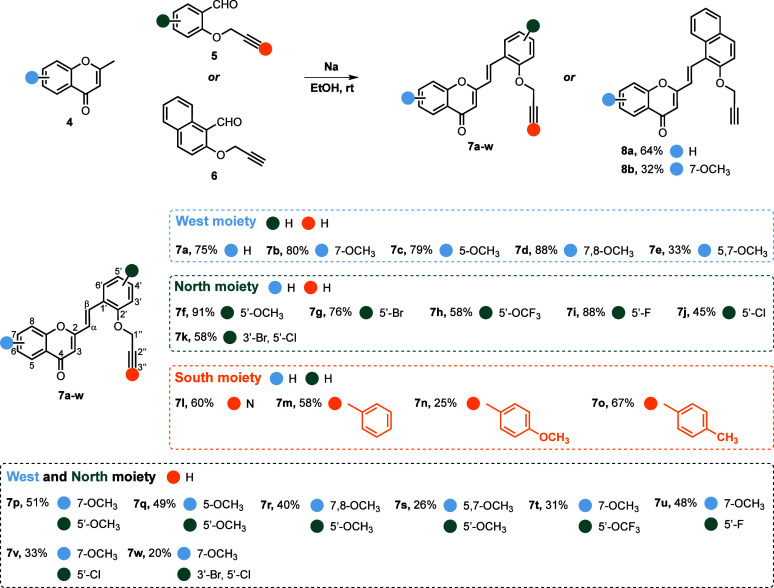
Synthesis of (*E*)-2-styrylchromones **7a**–**w** and **8a**–**b** ^a^

a Conditions: Na, EtOH, rt, 6 h.

**Scheme 2:**
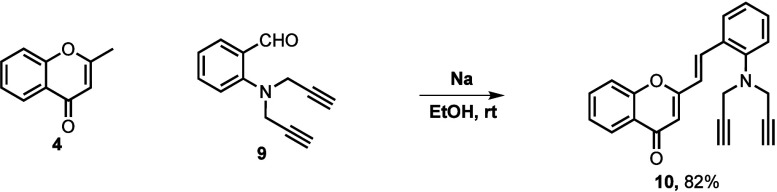
Synthesis of the (*E*)-2-styrylchromone **10** ^a^

a Conditions: Na, EtOH, rt, 6 h.

The second reaction step relies on microwave (MW)-assisted tandem intramolecular Diels–Alder (DA)/aromatization of (*E*)-2-styrylchromones **7a**–**k**, **7p**–**w** and **8a**–**b**, to obtain the target chromeno­[3,4-*b*]­xanthones **11a**–**s** and **12a**–**b** in 20–83% and 28–63% yields, respectively (Scheme 3).

**Scheme 3:**
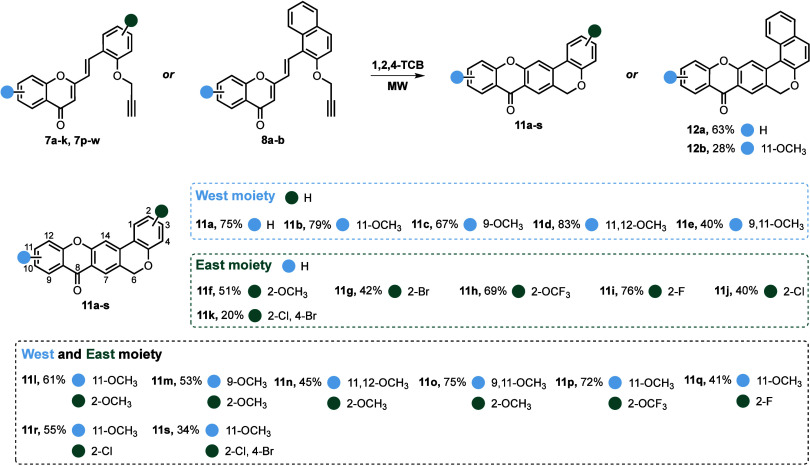
Synthesis of Chromeno­[3,4-*b*]­xanthones **11a**–**s** and **12a**–**b** ^a^

a Conditions: 1,2,4-TCB, MW, 220 °C, 30 min, then chloranil, 80 °C, 30 min.

### Biology

A library of 47 compounds was synthesized and categorized into two series (A and B) based on their structural features. To address distinct subpathologies of AD and assess their multifunctional profiles, both series were evaluated for their anticholinesterase activity (AChE and BChE), antiaggregation properties (Aβ and tau aggregation), and neuroprotective effects at enzymatic and cellular levels.

### Cholinesterase Inhibition Assays

Compound series A and B were initially screened for their cholinesterase activity against *ee*AChE (*Electrophorus electricus* AChE) and *eq*BChE (equine serum butyrylcholinesterase), using an adaptation of the Ellman’s method.^39^ The IC_50_ values of the most active compounds are summarized in Table 1, in comparison with the reference drug donepezil. Overall, series A displayed modest anticholinesterase activity, primarily targeting AChE (IC_50_ 2.4–9.5 μM), with low selectivity indices, indicating limited enzyme preference and potential dual inhibition. Among these, (*E*)-2-styrylchromones **7a**, **7d**, **7o** and **10** showed the most promising activity profiles, with compound **10** also exhibiting notable efficacy against BChE (IC_50_ 5.9 ± 0.4 μM) and dual activity (Table 1). Structural modifications significantly influenced the activity and selectivity of the compounds; for instance, the introduction of methoxy or halogen groups generally reduced potency, but affected enzyme preference differently, as seen in compound **7d**, which displayed a stronger anticholinesterase profile (Table 1). Similarly, replacing the terminal triple bond in **7l** with a nitrile group reduced its activity, while replacing the *O*-propargyl group with an *N,N-*dipropargyl group in **10** enhanced the inhibition of both enzymes, particularly BChE (Table 1). The installation of an additional aryl ring in **7o** further improved AChE inhibition, slightly increasing selectivity toward this enzyme. The superior performance of derivatives **11** and **12** compared to series A compounds highlights the enhanced potential of rigidified scaffolds for dual inhibition of both enzymes, though some compounds of this series also demonstrated moderate selectivity, suggesting structural rigidification also can influence enzyme specificity (Table 1). A closer examination of substitution effects reveals distinct structure–activity relationships (SAR). For derivatives **11d**, **11e** and **11m**, the presence of multiple methoxy groups in both west and east moieties appears to favor selective inhibition of BChE (IC_50_ 7.1–9.4 μM), while reducing efficacy against AChE (Table 1). Notably, the IC_50_ values for these compounds were lower than those of the reference drug, surpassing its inhibitory potential against BChE. This trend suggests that methoxy groups may contribute to selective interactions with BChE active site or steric hindrance at AChE binding pocket. In contrast, derivatives such as **11q**, **11r** and **11s** demonstrated improved dual inhibitory activity, particularly toward AChE, when methoxy groups on the west moiety were paired with halogens on the east side (IC_50_ 1.7–4.3 μM), although they were less potent than the reference drug (Table 1). This synergistic effect likely arises from complementary electronic and steric interactions within the enzyme active site, resulting in balanced inhibitory profiles with SI values closer to 1 (Table 1). Interestingly, derivatives **11h** and **11k** containing halogens exclusively on the east moiety, also exhibited significant anticholinesterase activity, highlighting the crucial role of halogen substitution in enhancing binding affinity and, in some cases, increasing selectivity toward AChE (Table 1). Compared to donepezil, which is highly selective for AChE (SI ∼ 633), most synthesized compounds exhibited lower selectivity, ranging from dual inhibition to moderate enzyme preference. While donepezil’s high selectivity is well aligned with its clinical profile, the broader activity spectrum of some of these compounds may offer advantages in targeting multiple cholinergic pathways, particularly during different stages of AD.

**Table 1:**
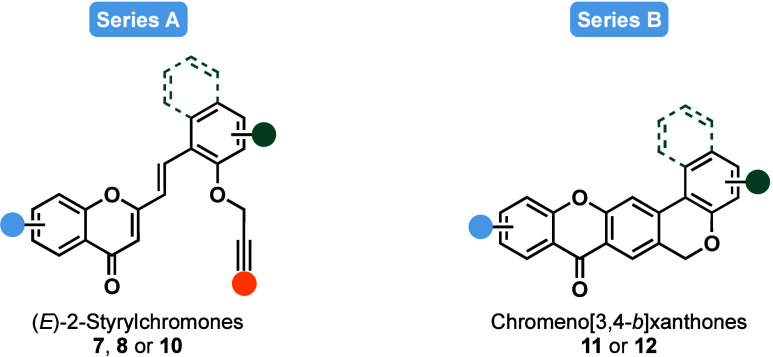
*In Vitro* Inhibitory Activity against *ee*AChE and *eq*BChE, and Aβ_42_ and Tau Protein Aggregation in *E. coli* Cells for Compounds **7**, **8** and **10** (Series A) and **11** and **12** (Series B)

**Compound**	**IC**_**50**_ *ee* **AChE (μM)** ^a^	**IC**_**50**_ *eq* **BChE (μM)** ^a^	**SI** *ee* **AChE** ^b^	**Aβ**_**42**_ **inhibition %** ^a^ **^,^** ^d^	**Tau inhibition %** ^a^ **^,^** ^d^
**Series A**					
**7a**	9.5 ± 0.3	n.a^c^		38.0 ± 6.2	
**7b**	n.a^c^	n.a^c^		23.0 ± 1.8	
**7c**	n.a^c^	n.a^c^		33.0 ± 2.2	
**7d**	2.4 ± 0.4	n.a^c^		28.0 ± 1.7	
**7e**	n.a^c^	n.a^c^		24.0 ± 1.7	
**7f**	n.a^c^	n.a^c^		15.0 ± 2.7	
**7g**	n.a^c^	n.a^c^		25.0 ± 4.6	
**7h**	n.a^c^	n.a^c^		11.0 ± 2.0	
**7i**	n.a^c^	n.a^c^		22.0 ± 4.8	
**7j**	n.a^c^	n.a^c^		11.0 ± 2.3	
**7k**	n.a^c^	n.a^c^		24.0 ± 3.0	
**7l**	n.a^c^	n.a^c^		34.0 ± 5.2	
**7m**	n.a^c^	n.a^c^		28.0 ± 3.8	
**7n**	n.a^c^	n.a^c^		34.0 ± 3.3	
**7o**	2.9 ± 0.5	n.a^c^		37.0 ± 2.0	
**7p**	n.a^c^	n.a^c^		11.0 ± 1.5	
**7q**	n.a^c^	n.a^c^		38.0 ± 6.6	
**7r**	n.a^c^	n.a^c^		40.0 ± 5.6	
**7s**	n.a^c^	n.a^c^		50.0 ± 1.6	
**7t**	n.a^c^	n.a^c^		20.0 ± 2.3	
**7u**	n.a^c^	n.a^c^		28.0 ± 3.5	
**7v**	n.a^c^	n.a^c^		17.0 ± 2.0	
**7w**	n.a^c^	n.a^c^		35.0 ± 3.0	
**8a**	n.a^c^	n.a^c^		47.0 ± 3.9	
**8b**	n.a^c^	n.a^c^		32.0 ± 4.3	
**10**	8.7 ± 0.5	5.9 ± 0.4	0.68	36.0 ± 18.3	
**Series B**					
**11a**	2.1 ± 0.2	n.a^c^		15.0 ± 4.6	
**11b**	3.9 ± 0.9	n.a^c^		22.0 ± 2.7	
**11c**	n.a^c^	n.a^c^		34.0 ± 4.4	
**11d**	n.a^c^	7.1 ± 0.1		40.0 ± 6.4	
**11e**	n.a^c^	9.4 ± 0.2		36.0 ± 4.4	
**11f**	6.9 ± 1.5	6.1 ± 0.5	0.88	36.0 ± 7.8	
**11g**	n.a^c^	n.a^c^		36.0 ± 4.8	
**11h**	4.0 ± 1.2	6.2 ± 0.7	1.55	41.0 ± 6.4	
**11i**	n.a^c^	n.a^c^		47.0 ± 8.4	52.0 ± 6.3
**11j**	n.a^c^	n.a^c^		45.0 ± 7.4	
**11k**	4.2 ± 0.9	6.2 ± 1.0	1.48	46.0 ± 3.7	52.0 ± 7.2
**11l**	4.8 ± 0.5	9.0 ± 0.5	1.88	40.0 ± 5.1	
**11m**	n.a^c^	7.3 ± 0.4		30.0 ± 6.6	
**11n**	n.a^c^	n.a^c^		34.0 ± 4.0	
**11o**	n.a^c^	n.a^c^		33.0 ± 4.3	
**11p**	n.a^c^	n.a^c^		29.0 ± 7.6	
**11q**	4.3 ± 1.5	n.a^c^		50.0 ± 6.7	55.0 ± 5.9
**11r**	2.1 ± 0.9	6.3 ± 0.6	3.00	57.0 ± 5.7	61.3 ± 5.9
**11s**	1.7 ± 1.2	7.0 ± 0.4	4.12	46.0 ± 6.3	58.0 ± 7.1
**12a**	n.a^c^	n.a^c^		43.0 ± 3.9	
**12b**	n.a^c^	n.a^c^		54.0 ± 6.2	57.0 ± 7.0
**Donepezil** ^e^	0.040 ± 0.002	25.32 ± 1.44	∼633		
**DP128** ^f^				78.2 ± 1.9	71.1 ± 1.3

a Data are expressed as the mean ± the standard error of the mean (SEM) of at least three experiments (*n* = 3), each performed in triplicate.

b Selectivity for *ee*AChE is determined as the ratio of *eq*BChE IC_50_/*ee*AChE IC_50_.

c n.a meaning nonactive.

d Screening concentration of 50 μM.

e Data taken from literature.^40^

f Used internal control.^37,41,42^

Overall, the structural rigidification inherent to the chromeno­[3,4-*b*]­xanthone scaffold emerged as a critical determinant of anticholinesterase activity, significantly enhancing the inhibitory potential compared to the more flexible series A compounds. Furthermore, the SAR analysis underscores the importance of substituent positioning, particularly combinations of methoxy and halogen groups, in fine-tuning enzyme specificity and dual inhibitory profiles. These results also highlight that even subtle structural modifications among closely related derivatives can profoundly influence enzyme binding, inhibitory potency, and selectivity, as well as steric interactions, binding orientation, and electronic complementarity within the enzyme gorge, emphasizing the critical role of precise molecular design in optimizing biological activity and target selectivity. Collectively, these findings suggest that the rigidified chromeno­[3,4-*b*]­xanthone derivatives could help mitigate cognitive impairment by targeting cholinesterase-related subpathologies of AD.

### Molecular Docking

Molecular docking studies were performed to evaluate the binding modes of the most promising compounds from each series against AChE and BChE. Tables S1 and S2 in Supporting Information summarize their main interactions and predicted binding poses. As some of the molecular binding modes have been previously reported in the literature, they will not be discussed further here.^25^ Based on the enzymatic assay results and the distinct structural characteristics of each series, we chose compounds **10** and **11r** as representative compounds, exemplifying the flexible and rigid scaffolds, respectively (Figure 4). In series A, derivative **10** was predicted to bind to both enzymes’ catalytic anionic site (CAS) and peripheral anionic site (PAS), entering favorably through its west moiety. A hydrogen bond (HB) was observed between its ether group and the catalytic serine residue, disrupting the usual HB between the latter and the catalytic histidine; π-stacking interactions with aromatic residues (Trp286 of AChE; Asp70 and Tyr332 of BChE) further stabilized its binding (Figure 4). In series B, chromeno­[3,4-*b*]­xanthone **11r** exhibited a unique binding profile, with two predicted binding entrances (via the west or east moiety) into the enzymes’ binding channels. In AChE, the west moiety was favored due to the presence of halogen groups in the east moiety, combined with a methoxy group in the west. This combination facilitated anchoring to Leu130 through hydrophobic, π-stacking and halogen-π interactions (Figure 4). Conversely, in BChE, **11r** preferred the east moiety entrance, where the chloride substituent was predicted to interact directly with Ser198 via a halogen bond (δ-hole). This was complemented by π-stacking interactions with Phe329 and Tyr332, as well as a short HB with Ser72 (Figure 4).

**Figure 4:**
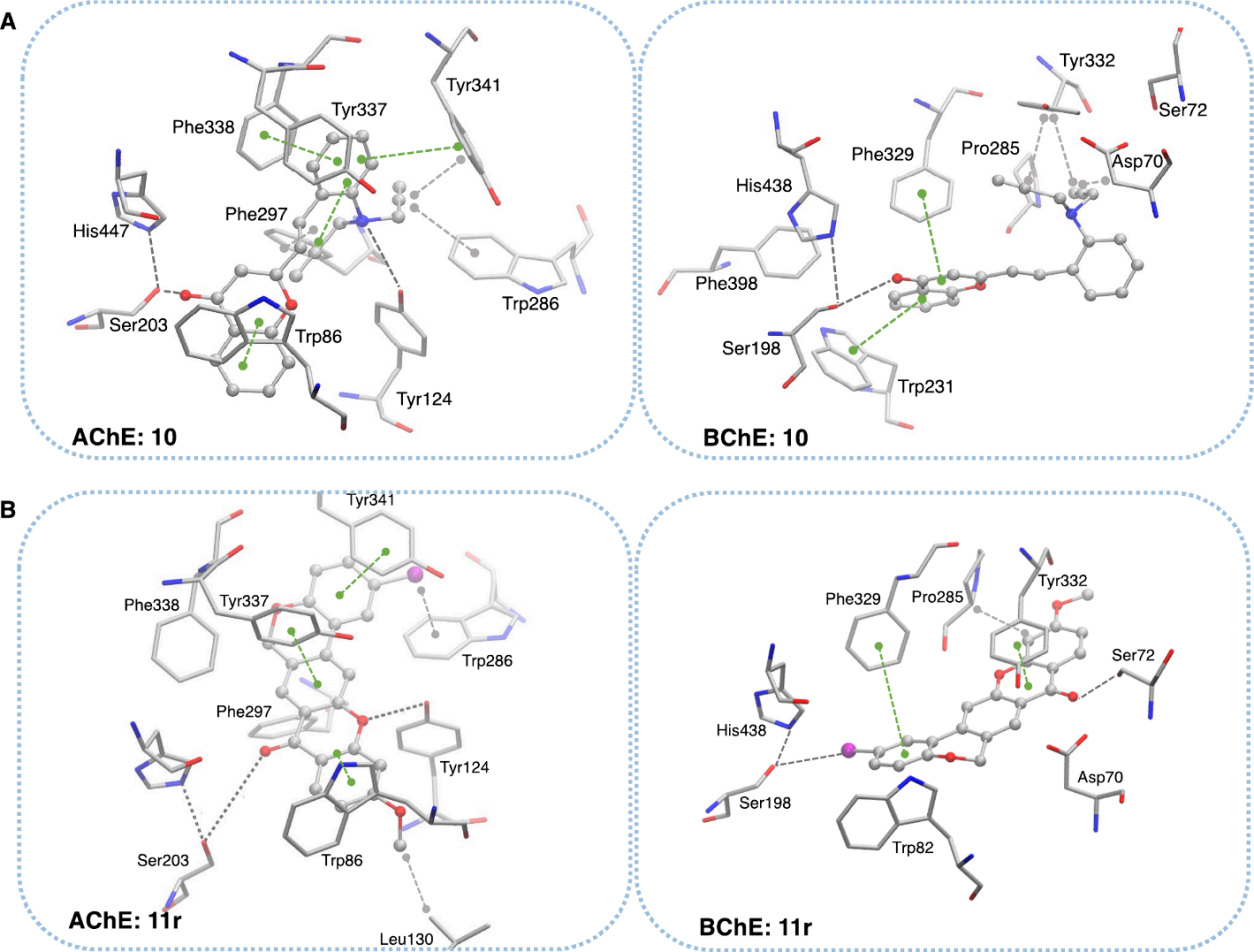
Binding poses of the representative compounds from series A and B on the active sites of AChE (left) and BChE (right): (A) compound **10** and (B) compound **11r**. The compounds are represented with balls-and-sticks and colored by atom type, while the interacting residues are represented in sticks and colored by atom type.

Overall, the docking results suggest that π-stacking interactions with Trp86 and Tyr337 in AChE, and Trp231 in BChE were critical for activity. These computational predictions highlight key structural features that contribute to activity and provide a foundation for further scaffold optimization to enhance potency and multifunctionality.

### Kinetic Analysis

Based on the computational predictions, derivatives **10** (series A) and **11r** (series B) were initially expected to interact with both CAS and PAS of AChE and BChE. However, to better understand the actual mechanism of action of these compounds, kinetic studies were performed (Figure 5). The Lineweaver–Burk plots demonstrated that both derivatives displayed reversible competitive inhibition toward both enzymes, with a series of lines showing the same y-intercept as the enzymes without the compounds (Figure 5A and 5B). These results indicate that the compounds bind specifically to the CAS of both enzymes, preventing the substrate binding, which contrasts with the initial computational predictions.

**Figure 5:**
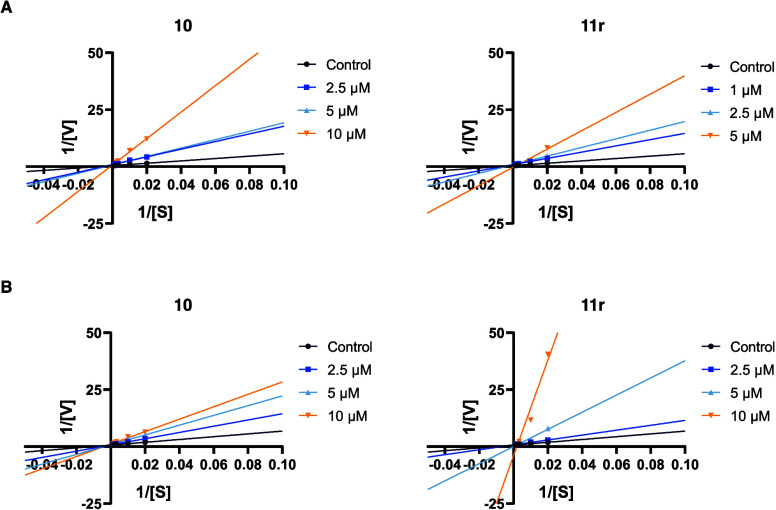
Merged Lineweaver–Burk reciprocal plots of (A) AChE and (B) BChE initial velocity with increasing substrate concentration (50–800 μM) in the absence or presence of the compounds **10** and **11r**. Lines were derived from a weighted least-squares analysis of data points.

### Aβ_42_ and Tau Protein Aggregation Inhibition

The antiaggregating profiles of both series A and B were evaluated using a fluorescence Thioflavin S (ThS) assay *in cellulo*, which monitors ThS fluorescence in the presence of β-sheet-rich structures, such as Aβ and tau protein aggregates.^43^ This method, based on recombinant *Escherichia coli* (*E. coli*) bacteria overexpressing insoluble aggregates, offers a simple and cost-effective approach to model aggregation processes in mammalian systems and has been validated for use in drug discovery studies.^36,42,44^ Compounds were tested at a screening concentration of 50 μM for their ability to inhibit Aβ_42_ aggregation. The results, summarized in Table 1, reveal distinct antiaggregating profiles between series A and B, which are once again influenced by their structural differences.

Compounds from series A exhibited modest antiaggregating activity against Aβ_42_, within inhibition percentages ranging from 11–50% (Table 1). Derivatives **7s** and **8a** showed the best performance within this series, inhibiting Aβ_42_ aggregation by 50% and 47%, respectively (Table 1). Structural modifications, such as the introduction of methoxy or halogen groups, generally decreased antiaggregating efficacy, with the exceptions of **7r** and **7s** (Table 1), suggesting that these substitutions can differentially modulate interactions critical for inhibiting aggregation. Other modifications, including replacing the terminal triple bond with a nitrile group (**7l**), introducing an aryl ring (**7m**–**o**), or substituting the *O*-propargyl group with an *N,N-*dipropargyl group (**10**) did not significantly alter the activity profile.

Although the antiaggregating percentages for series B were overall higher than series A (ranging from 15–57%), the differences in activity strength were not as pronounced (Table 1). Substituents that decreased activity in series A often had the opposite effect in series B. For instance, the introduction of methoxy and halogen groups enhanced the antiaggregating potential of derivatives **11i**, **11k**, **11q**-**s** and **12b**. Among these, **11r** emerged as the most potent, displaying a 57% inhibition of Aβ_42_ aggregation (Table 1). This contrasting behavior, also observed in the cholinesterase inhibition assay, underscores how subtle structural modifications can significantly influence binding interaction and aggregation inhibition.

Given the superior antiaggregating activity of series B against Aβ_42_, their potential to inhibit tau protein aggregation–another key target within the same subpathology–was also evaluated. This strategic extension aimed to validate the chromeno­[3,4-*b*]­xanthone scaffold for related aggregation targets, considering the structural and pathological similarities between Aβ and tau aggregation processes. Preliminary results showed that the selected compounds (**11i**, **11k**, **11q**, **11r**, **11s**, **12b**) displayed a similar antiaggregating profile against tau with inhibition percentages ranging from 52–61% (Table 1). Once again, compound **11r** stood out by exhibiting the highest inhibition of tau protein aggregation (61.3 ± 5.9%), mirroring its performance against Aβ aggregation. Interestingly, these derivatives exhibited a higher propensity to inhibit tau protein aggregation compared to Aβ_42_, suggesting potential target specificity influenced by substituent effects and scaffold conformation. Nevertheless, it is important to interpret the results cautiously, as the inability of certain compounds to cross bacterial membranes, may have underestimated their true inhibitory activity in the assay.^45^

Overall, these results highlight the role of structural rigidity and substituent positioning in the antiaggregating properties of chromeno­[3,4-*b*]­xanthone derivatives. The validation of series B against tau protein aggregation further underscores the scaffold’s versatility, as a potential therapeutic framework. Together, these findings establish a strong foundation for developing multifunctional agents capable of addressing distinct subpathologies of AD, such as Aβ and tau aggregation, across different stages of the disease.

### *In Silico Study*: Influence on Aβ Aggregation

A blind docking approach was performed to identify the binding site and interactions between compound **11r** (strongest inhibitor of the series) and the LS-shaped Aβ_42_ fibril. This specific Aβ fibril (PDB ID: 5oqv) was selected because it is one of the most recent structures available and, unlike other structures in the PDB, it comprises the **full-length Aβ**_**42**_ **peptide** (residues 1–42). Other available fibril structures often represent truncated forms or fragments of the Aβ peptide, making this complete version particularly valuable for accurately studying aggregation and interaction mechanisms. Two main binding sites were observed (Figure 6). Only binding site 1 was considered for this study, as it exhibited the highest number of conformations, and compound **11r** showed significantly better binding energy to this site than to binding site 2. The docking results demonstrated a strong binding free energy of −11.3 kcal/mol for the best docking pose. The ligand is stabilized by hydrophobic interactions with Phe19, Asn27, and Ile31, but polar interactions were also identified, including an HB with Ala30 and a halogen bond with Ser26. These findings suggest that compound **11r** binds solidly to the Aβ_42_ fibril.

**Figure 6:**
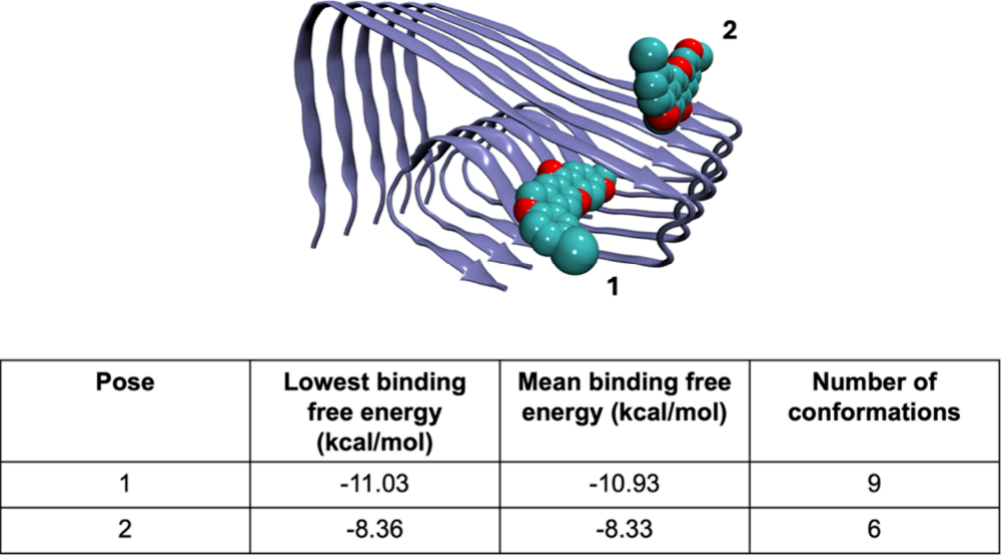
Compound **11r** binding to Aβ_42_ fibril. The image shows two binding sites (1 and 2) identified through molecular docking. The table displays the lowest binding energy, mean binding energy, and cluster counts for each pose.

A molecular dynamics (MD) simulation was performed to further validate the stability of the complex and investigate its binding dynamics and impact on fibril growth. Throughout the simulation, a rearrangement between the ligand and the fibril led to new interactions, including a hydrophobic contact with Val24, an HB with Asn27, and a key π-stacking interaction with residue Phe19 that is crucial for the stability of the ligand-protein binding. Compound **11r** remained bound to the Aβ_42_ fibril throughout the entire simulation time, and although it did not impact the structural stability of the fibril, it occupied a binding site that sterically inhibits the lateral association of an additional Aβ monomer, which should prevent fibril growth (Figure 7). Given the stability of this interaction and the strategic positioning of the ligand at a fibril growth interface, these results suggest that compound **11r** may act as a structural barrier to Aβ elongation.

**Figure 7:**
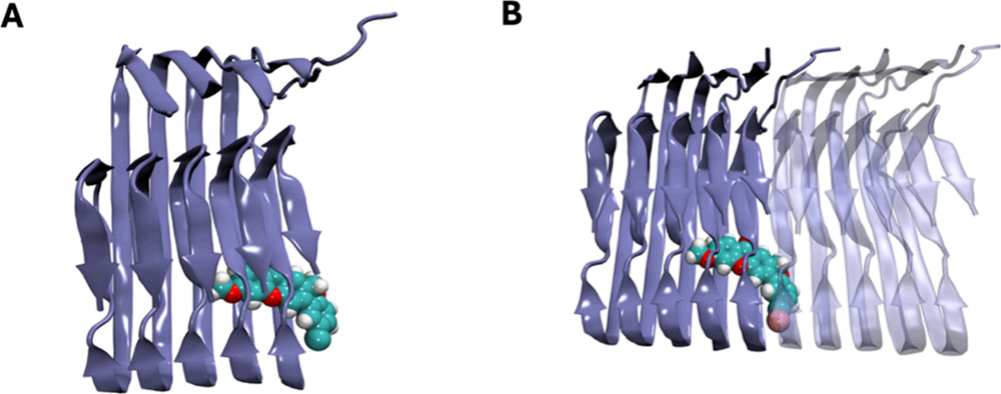
Ligand interaction of compound **11r** with an Aβ_42_ fibril. (**A**) Complex after 100 ns of MD, showing the stabilized binding. (**B**) Overlay with a transparent fibril model illustrates how ligand binding may block further fibril association.

### BBB Permeability Prediction

The BBB is a highly selective, semipermeable barrier that regulates the CNS microenvironment.^46^ While it effectively blocks the entry of blood-borne substances into the brain, it also prevents over 98% of small-molecule drugs and macromolecular therapeutics from accessing the CNS.^46^ Based on the results of the cholinesterase and protein aggregation inhibition assays, five compounds were selected (**10**, **11k** and **11q**–**s**) for testing their ability to penetrate the BBB (Table 2). The selection focused on compounds that inhibited three or more relevant targets by over 50% for each target at their screening concentration, highlighting their multifunctional activity. Compound **10** was also included due to its structural profile. To assess BBB permeability, a parallel artificial membrane permeability assay (PAMPA)-BBB was performed, which predicts passive BBB permeability using a brain lipid porcine membrane.^47^ The *in vitro* effective permeability (*P*_e_) of commercial drugs was determined and compared to reported values for assay validation, demonstrating good correlation between experimental and reference data (Figure S3). According to established BBB permeation criteria, compounds with a permeability higher than 3.93 × 10^–6^ cm s^–1^ were classified as CNS+.^47^ Based on these results, four out of five compounds were predicted to cross the BBB via passive permeation (except **11k**), making them suitable for further evaluation (Table 2).

**Table 2:** Permeability Results *P*_*e*_ (10^–6^cms^–1^) from the PAMPA-BBB Assay for 10 Commercial Drugs (Used in the Experiment Validation) and the Selected Compounds with Their Predicted Penetration into the CNS^a^

Compound	Bibl.^b^	*P*_*e*_ (10^–6^cms^–1^)^c^	Prediction^d^
Atenolol	0.8	0.42 ± 0.19	
Caffeine	1.3	0.53 ± 0.01	
Desipramine	12	15.63 ± 1.78	
Enoxacin	0.9	0.15 ± 0.15	
Hydrocortisone	1.9	0.57 ± 0.06	
Ofloxacin	0.8	0.62 ± 0.78	
Piroxicam	2.5	0.19 ± 0.02	
Promazine	8.8	15.38 ± 0.91	
Testosterone	17	13.99 ± 2.21	
Verapamil	16	15.18 ± 0.85	
**10**		11.62 ± 3.39	CNS+
**11k**		2.30 ± 1.11	CNS±
**11q**		6.04 ± 1.99	CNS+
**11r**		7.12 ± 0.89	CNS+
**11s**		8.70 ± 0.61	CNS+

a PBS:EtOH (70:30) was used as solvent.

b Reference Di et al.^47^

c Data are the mean ± standard deviation (SD) of two independent experiments.

d Ranges of permeability of PAMPA-BBB assays: High BBB permeation predicted (CNS+) *P*_e_ > 3.93; Uncertain BBB permeation (CNS±) 3.93 > *P*_e_ > 1.82; Low BBB permeation predicted (CNS-) *P*_e_ < 1.82.

While PAMPA-BBB effectively predicts passive diffusion, it does not account for active transport mechanisms, such as efflux mediated by ATP-binding cassette (ABC) transporters.^48^ To complement this approach and better understand whether these compounds may be substrates of such transporters, we next employed the calcein-AM-assay. This assay is particularly useful for assessing P-glycoprotein (P-gp), a major efflux transporter at the BBB that actively limits drug accumulation in the brain.^48^ To investigate potential interactions with P-gp, compounds **10**, **11q**-**s** were tested at concentrations of 1 μM, 10 μM, 50 μM and 100 μM, using the calcein-AM-assay in hMEC/D3 cells (Figure 8). The results showed that **11q**–**s** yielded consistently low fluorescence values across all concentrations, indicating only weak–if any–interaction with P-gp and suggesting that they are unlikely to be actively effluxed (Figure 8). Therefore, these compounds are not expected to be P-gp substrates and may have favorable capacity to cross the BBB via passive diffusion, making them promising candidates for CNS drug development. In contrast, compound **10** demonstrated a clear inhibitory effect on P-gp activity, as evidenced by increased intracellular fluorescence, suggesting that it either inhibits P-gp activity or is retained within the cells due to reduced efflux (Figure 8).

**Figure 8:**
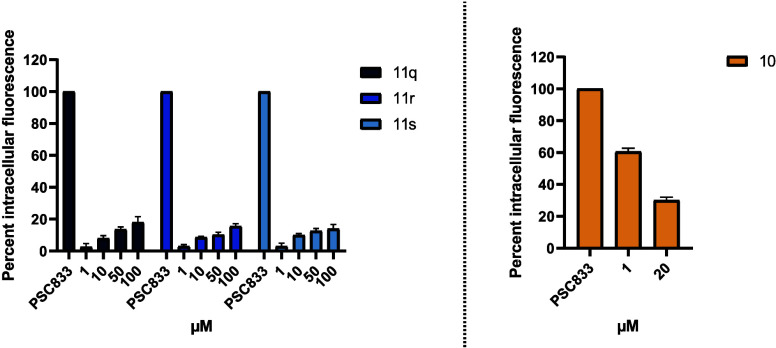
Evaluation of P-gp interaction by compounds **10** and **11q**–**s** using the calcein-AM-assay in hMEC/D3 cells. Intracellular fluorescence was measured following treatment with compounds at concentrations of 1–100 μM. Data are presented as mean ± SEM (*n* = 3).

### Cytotoxicity Assessment

The neurotoxicity of the compounds predicted to cross the BBB was assessed using the 3-[4,5-dimethylthiazol-2-yl]-2,5-diphenyltetrazolium bromide (MTT) viability assay. Human neuroblastoma cells (SH-SY5Y cell line) were treated with increasing concentrations of the compounds (1–100 μM) (Figure 9). In general, most compounds were well tolerated at their lowest concentrations, which corresponded to their effective concentrations toward the targets (Figure 9). However, the chromeno­[3,4-*b*]­xanthone scaffold exhibited neurotoxic effects, with cell viability dropping below 80% at higher concentrations. The hydrophobic and aromatic nature of this scaffold may enhance the compounds’ ability to penetrate cell membranes and exert their therapeutic effects, while minimizing toxicity at the active concentrations used. It is also worth noting that *in vitro* toxicity does not always reflect *in vivo* outcomes, as metabolic and clearance mechanisms present *in vivo* may mitigate the effects observed in cell-based assays.^49,50^

**Figure 9:**
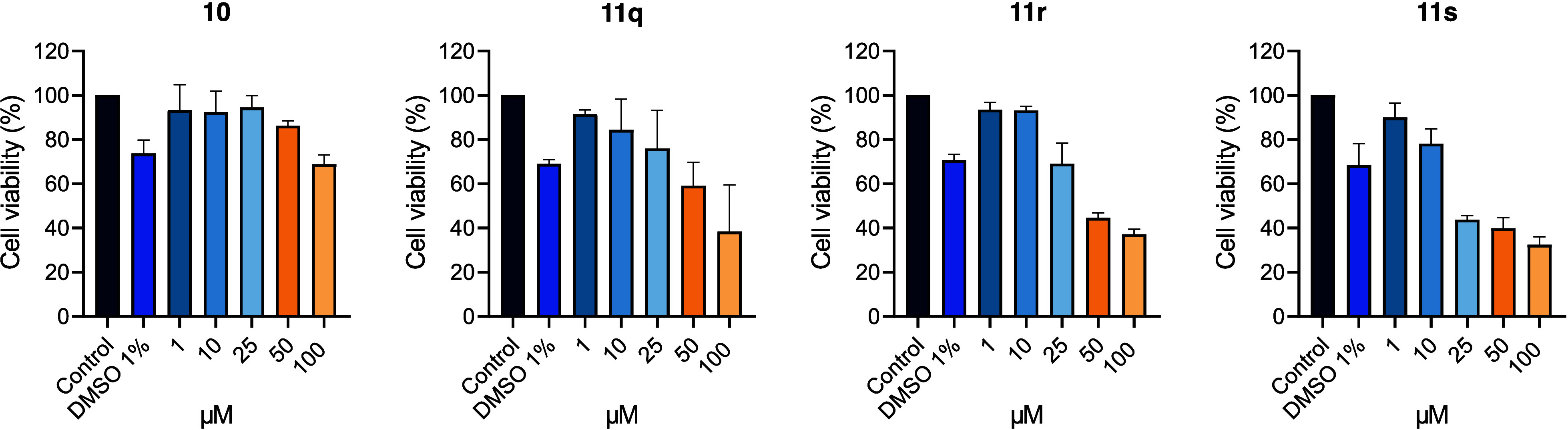
Neurotoxicity effect of compounds **10** and **11q**-**s** and vehicle group (DMSO 1%) in SH-SY5Y cells after 24 h of incubation. The results are expressed as the average of viability of at least 3 experiments ± SEM, comparing to the control (untreated cells).

### Interaction with Aβ_42_ Peptide

We selected compound **11r** for further investigation of its ability to interfere with the Aβ_42_ aggregation process *in vitro*. The decision was based on the structural potential of the chromeno­[3,4-*b*]­xanthone scaffold, the encouraging results for **11r**, and its overall profile in our preliminary studies. While other compounds also demonstrated a multifunctional profile against AD-related subpathologies, **11r** was chosen as a representative candidate to further validate and explore the potential of this scaffold. The morphological changes of Aβ_42_ species were assessed through transmission electron microscopy (TEM), in the presence and absence of **11r**. TEM analysis revealed significant inhibition of the Aβ aggregation process, as evidenced by distinct morphological differences in the amyloid species during the 48 h incubation period with the compound (Figure 10). In the absence of **11r** (Figure 10, top panels), as early as 6 h of incubation at 37 °C, (white arrow), short (white arrowhead) and long fibrils were detected. As incubation progressed, long fibrils were more abundant, while other species became scarce, resulting in bundles of tangled fibrils after 48 h (Figure 10). Mature fibrils showed typical diameters of approximately 10–12 nm. Conversely, in the **11r**-treated samples, mainly amorphous aggregates were detected (blue arrows, Figure 10, bottom panels), and although a few fibrils were visualized, these were significantly shorter (Figure 10, bottom panels). These results demonstrated that **11r** clearly affects the amyloid aggregation process, inhibiting Aβ_42_ fibrillogenesis.

**Figure 10:**
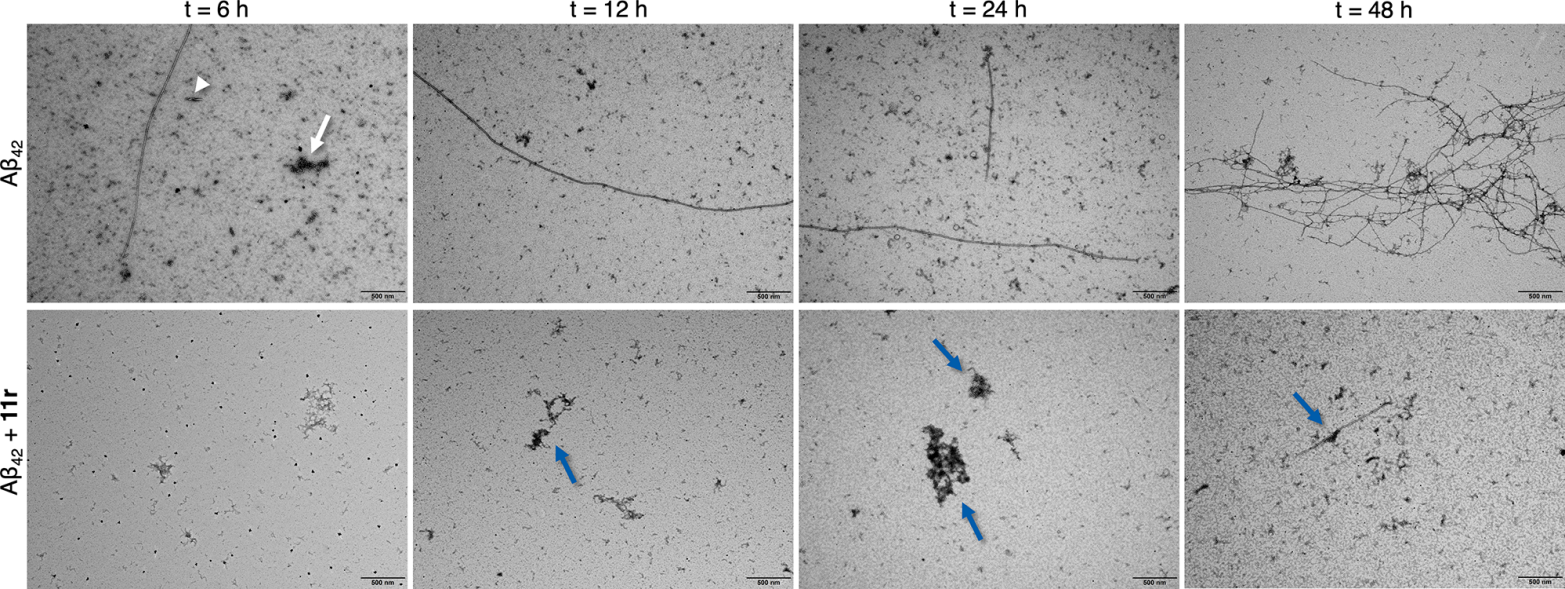
TEM images of Aβ_42_ fibrils growth (10 μM) at four different time-points (6, 12, 24, and 48 h), in the absence or presence of compound **11r** (50 μM). White arrow and arrowhead pinpoint an amorphous aggregate and short fibril, respectively, in the Aβ_42_ preparation, in the absence of **11r**. Blue arrows pinpoint amorphous aggregates and short Aβ_42_ fibrils upon treatment with **11r**. Scale bar = 500 nm.

Building on these results, we investigated the cellular effects and disaggregation potential of compound **11r**, using SH-SY5Y exposed to synthetic Aβ peptide (cells stably expressing the wild-type 695 isoform of amyloid precursor protein (APP695, SHwt). Given the cytotoxicity observed at higher concentrations of **11r** in our cytotoxicity assessment, the concentration used in this experiment was selected to minimize any potential interference from compound-induced toxicity, ensuring that the observed effects on Aβ aggregation were not confounded by cell damage. Therefore, cells were incubated for 48 h with either Aβ_42_ alone or a mixture of Aβ_42_ and **11r**.

Although extracellular Aβ deposit is the major form in the brain of AD patient, some evidence have indicated a potential pathogenic relevance on intracellular Aβ accumulation.^51,52^ Aβ aggregates can interact with various cellular organelles (e.g., lysosomes and mitochondria), disrupting synaptic function and triggering proteasome dysfunction and calcium dyshomeostasis. Given this, we tested the efficacy of our lead compound **11r** in lowering intracellular aggregates.

Following incubation, cells were fixed, stained with Phalloidin and Thioflavin T (ThT), and analyzed by confocal microscopy to evaluate whether coincubation with **11r** reduced Aβ aggregate formation (Figure 11A). Intracellular ThT fluorescence, indicative of the accumulation of Aβ aggregates, was uniformly distributed in the control group, indicating extensive Aβ aggregation (Figure 11A). However, in the presence of **11r**, fluorescence intensity was significantly reduced, demonstrating its ability to disrupt the amyloid aggregation process in this cell model of AD (Figure 11A). Quantitative analysis revealed an approximately 70% reduction in aggregates in cells treated with **11r**, further supporting the therapeutic potential of this compound (Figure 11B). Interestingly, this effect was observed at a lower concentration (25 μM) in the cellular assay compared to the bacterial assay, where the compound inhibited only 57% of the aggregation process at 50 μM. These findings highlight potential differences between the two assay systems, which could be influenced by factors such as membrane permeability, compound uptake or other cellular dynamics.

**Figure 11:**
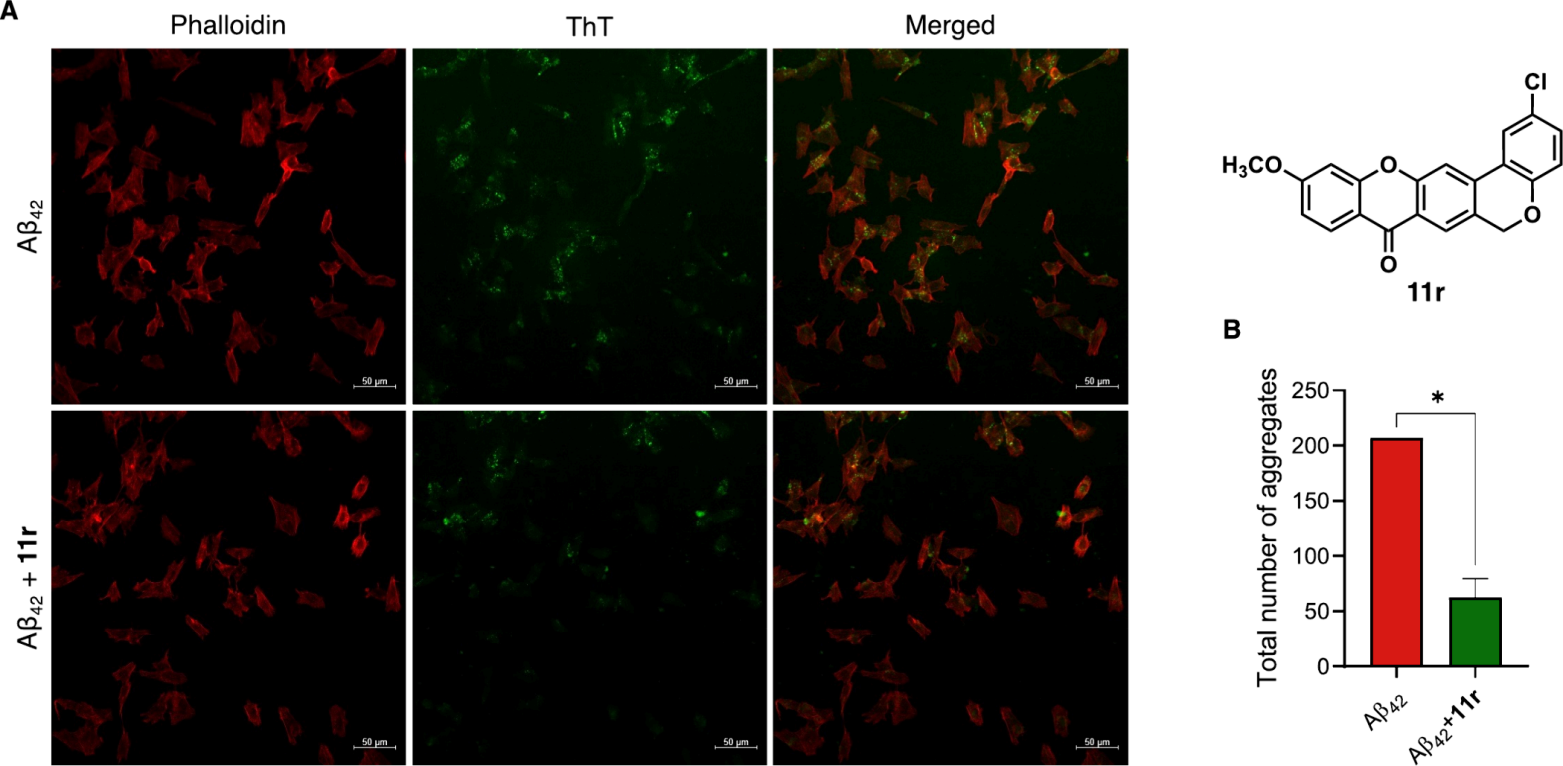
(A) Confocal images of SH-SY5Y cells treated with Aβ_42_ (10 μM) and compound **11r** (25 μM) mixed with Aβ_42_ for 48 h, stained with Phalloidin (red) and ThT (green). (B) Quantitative analysis of Aβ_42_ accumulation in cells treated with Aβ_42_ alone or in combination with compound **11r**. Data are shown as mean ± SEM (*n* = 3). Statistical significance was assessed using an unpaired two-tailed Student’s *t* test. *p < 0.05.

### Theoretical Prediction of ADME Properties

The drug-like profiles of compound **11r** was also investigated, with ADME properties estimated using the SwissADME web tool. As shown in Table 3, **11r** was predicted to exhibit favorable drug-likeness properties, fulfilling Lipinski’s and Veber’s rules, and lacking structural features of pan-assay interference compounds (PAINS). The fact that this compound satisfies these rules suggests it possesses the potential for a favorable pharmacokinetic profile, which is a critical step toward its development as a therapeutic agent. Furthermore, the absence of PAINS-like features further supports its suitability by reducing the likelihood of false positives in assay testing.

**Table 3:** Drug-Likeness Properties of the Most Promising Compound **11r**

Compound	MW^a^	nrotb^b^	nON^c^	nOHNH^d^	tPSA^e^	ilogP^f^	PAINS alerts
**11r**	364.78	1	4	0	48.67	3.62	0

a Molecular weight.

b Number of rotatable bonds.

c Number of hydrogen acceptors.

d Number of hydrogen donors.

e Total polar surface area.

f Octanol/water partition coefficient.

## Conclusions

AD remains one of the most challenging conditions to treat, and despite extensive research, current therapeutic approaches have not provided a lasting solution. Small molecules offer a cost-effective and versatile treatment option, enabling early intervention and long-term patient adherence. Our study expanded the chromeno­[3,4-*b*]­xanthone scaffold, as an alternative small-molecule lead that warrants further investigation in AD drug discovery, due to its potential to target distinct subpathologies at different stages of the disease.

Our results revealed that these compounds exhibit a potent cholinesterase inhibition and effective inhibition of amyloid aggregation at micromolar concentrations. Notably, compounds **10** and **11q**–**s** demonstrated strong inhibitory effects with IC_50_ values ranging from 1.7–9.0 μM for AChE and BChE. Molecular docking studies confirmed that these compounds bind to the active sites of both enzymes via hydrogen bonds and π-stacking interactions, with methoxy and halogen groups further enhancing binding affinity. In addition, the chromeno­[3,4-*b*]­xanthone compounds showed promising results in inhibiting Aβ aggregation and also tau protein aggregation, with inhibition exceeding 50% at 50 μM. Among them, compound **11r** consistently stood out, exhibiting the most well-balanced multifunctional profile across all evaluated targets, including potent dual cholinesterase inhibition and the highest inhibition percentages of both Aβ and tau protein aggregation (57.0 ± 5.7 and 61.3 ± 5.9, respectively). *In silico* calculations demonstrate that compound **11r** binds strongly to the LS-shaped Aβ_42_ fibril, forming key interactions, including a crucial π-stacking with Phe19, and occupies a strategic binding site that inhibits fibril lateral growth, highlighting its potential as an inhibitor of Aβ elongation. TEM analysis confirmed these findings, revealing significant differences in the morphology of Aβ aggregates, particularly during the early stages of aggregation. Further validation in SH-SY5Y cells demonstrated that compound **11r** reduced intracellular Aβ aggregation, supporting its potential as a therapeutic candidate. Moreover, these compounds exhibited favorable drug-like properties, including low cytotoxicity, a predicted ability to cross the BBB, which is crucial for AD treatment. The calcein-AM-assay also indicated a weak interaction with P-gp suggesting that they are unlikely to be actively effluxed from the brain.

In summary, our findings highlight the chromeno­[3,4-*b*]­xanthone scaffold as a promising lead for developing new small-molecule therapies for AD. Unlike traditional MTDL approaches, this strategy offers a more flexible and personalized therapeutic solution. Ongoing efforts aim to optimize this scaffold into theranostic agents capable of providing therapeutic benefits while also supporting diagnostic imaging, potentially enabling real-time monitoring and a more effective disease management.

## Experimental Section

### Chemistry

Melting points were measured with a Büchi B-540 apparatus. Nuclear magnetic resonance (NMR) spectra were recorded with a Bruker Avance 300 (300.13 MHz for ^1^H and 75.47 MHz for ^13^C) spectrometer, unless stated otherwise. Chemical shifts (δ) are reported in ppm and coupling constants (*J*) in Hz; the internal standard was tetramethylsilane (TMS). Unequivocal ^13^C assignments were made with the aid of 2D *g*HSQC and *g*HMBC (delays for one-bond-long-range *J*_*C/H*_ couplings were optimized for 145 and 7 Hz, respectively) experiments. Positive-ion electrospray ionization (ESI) mass spectra were acquired with a quadrupole time-of-flight (QTOF) 2 instrument [dilution of 1 μL] of the sample in chloroform solution (ca. 10–5 mL) in 200 μL of 0.1% trifluoroacetic acid/methanol solution. Nitrogen was used as the nebulizer gas and argon as the collision gas. The needle voltage was set at 3000 V, with the ion source at 80 °C and the desolvation temperature at 150 °C. The cone voltage was 35 V. Other lowand high-resolution mass spectra (EI, 70 eV) were measured with VG Autospec Q and M spectrometers. Preparative thin-layer chromatography (TLC) was performed with Merck silica gel (60 DGF254). All chemicals and solvents used were obtained from commercial sources and used as received or dried using standard procedures. MW-assisted reactions were carried out in a CEM Discover SP apparatus.

### General Procedure for the Synthesis of Compounds **7a-w**, **8a-b** and **10**

The appropriate 2-methylchromone **4** (1 mmol) and *O*- or *N-*propargylbenzaldehyde **5**, **6** or **7** (160 mg, 1 mmol) were added to a mixture of sodium (90 mg, 4 mmol) in EtOH (5 mL). The resulting mixture was stirred at room temperature for 3–6 h (monitored by TLC). After that period, the mixture was poured into water (30 mL) and ice (20 g) and the pH adjusted to 4 with dilute HCl (10%). The precipitate was rinsed with water, collected by filtration, taken in dichloromethane (DCM) and purified by preparative TLC, using DCM as eluent.

#### (*E*)-2-[2-(Prop-2-yn-1-yloxy)­styryl]-4*H*-chromen-4-one (**7a**)

yellow solid; yield 272 mg (75%). Both spectroscopic and analytic data are in accordance with those previously reported.^53^

#### (*E*)-7-Methoxy-2-[2-(prop-2-yn-1-yloxy)­styryl]-4*H*-chromen-4-one (**7b**)

yellow solid; yield 292 mg (80%). Both spectroscopic and analytic data are in accordance with those previously reported.^53^

#### (*E*)-5-Methoxy-2-[2-(prop-2-yn-1-yloxy)­styryl]-4*H*-chromen-4-one (**7c**)

yellow solid; yield 289 mg (79%). Both spectroscopic and analytic data are in accordance with those previously reported.^53^

#### (*E*)-7,8-Dimethoxy-2-[2-(prop-2-yn-1-yloxy)­styryl]-4*H*-chromen-4-one (**7d**)

yellow solid; yield 287 mg (88%). Both spectroscopic and analytic data are in accordance with those previously reported.^25^

#### (*E*)-5,7-Dimethoxy-2-[2-(prop-2-yn-1-yloxy)­styryl]-4*H*-chromen-4-one (**7e**)

yellow solid; yield 109 mg (33%). Both spectroscopic and analytic data are in accordance with those previously reported.^53^

#### (*E*)-2-[5-Methoxy-2-(prop-2-yn-1-yloxy)­styryl]-4*H*-chromen-4-one (**7f**)

yellow solid; yield 304 mg (91%). Both spectroscopic and analytic data are in accordance with those previously reported.^25^

#### (*E*)-2-[5-Bromo-2-(prop-2-yn-1-yloxy)­styryl]-4*H*-chromen-4-one (**7g**)

yellow solid; yield 348 mg (76%). Both spectroscopic and analytic data are in accordance with those previously reported.^25^

#### (*E*)-2-[2-(Prop-2-yn-1-yloxy)-5-(trifluoromethoxy)­styryl]-4*H*-chromen-4-one (**7h**)

yellow solid; yield 271 mg (58%); m.p 156–157 °C. ^1^H NMR (500 MHz, CDCl_3_): δ = 2.61 (t, 1H, H-3”, *J* 2.4 Hz), 4.85 (d, 2H, H-1”, *J* 2.4 Hz), 6.38 (s, 1H, H-3), 6.89 (d, 1H, H-α, *J* 16.2 Hz), 7.09 (d, 1H, H-3′, *J* 9.0 Hz), 7.23 (dd, 1H, H-4’, *J* 9.0, 2.4 Hz), 7.41 (ddd, 1H, H-6, *J* 8.0, 7.1, 1.1 Hz), 7.46 (d, 1H, H-6’, *J* 2.4 Hz), 7.57 (dd, 1H, H-8, *J* 8.6, 1.1 Hz), 7.70 (ddd, 1H, H-7, *J* 8.6, 7.1, 1.7 Hz), 7.85 (d, 1H, H-β, *J* 16.2 Hz), 8.20 (dd, 1H, H-5, *J* 8.0, 1.7 Hz) ppm. ^13^C NMR (125 MHz, CDCl_3_): δ = 56.7 (C-1”), 76.6 (C-3”), 77.7 (C-2”), 111.1 (C-3), 113.8 (C-3′), 118.0 (C-8), 120.5 (q, 5′-O*C*F_3_, *J* 257.0 Hz), 120.9 (C-6’), 122.7 (C-α), 123.2 (C-4’), 124.1 (C-4a), 125.1 (C-6), 125.7 (C-5), 126.0 (C-1’), 130.5 (C-β), 133.8 (C-7), 143.4 (C-5′), 154.2 (C-2’), 156.0 (C-8a), 161.6 (C-2), 178.5 (C-4) ppm. ^19^F NMR (282 MHz, CDCl_3_): δ −54.86 (s) ppm. HRMS (ESI^+^): *m*/*z* [M + H]^+^ calcd for C_21_H_14_O_4_F_3_ 387.0839; found 387.0823.

#### (*E*)-2-[5-Fluoro-2-(prop-2-yn-1-yloxy)­styryl]-4*H*-chromen-4-one (**7i**)

yellow solid; yield 338 mg (88%); m.p 150–152 °C. ^1^H NMR (300 MHz, CDCl_3_): δ = 2.58 (t, 1H, H-3”, *J* 2.4 Hz), 4.81 (d, 2H, H-1”, *J* 2.4 Hz), 6.36 (s, 1H, H-3), 6.85 (d, 1H, H-α, *J* 16.2 Hz), 7.03–7.08 (m, 2H, H-3′, H-4’), 7.32 (dd, 1H, H-6’, *J* 8.8, 2.4 Hz), 7.40 (ddd, 1H, H-6, *J* 8.3, 7.1, 0.9 Hz), 7.56 (dd, 1H, H-8, *J* 8.6, 0.9 Hz), 7.69 (ddd, 1H, H-7, *J* 8.6, 7.1, 1.7 Hz), 7.87 (d, 1H, H-β, *J* 16.2 Hz), 8.20 (dd, 1H, H-5, *J* 8.3, 1.7 Hz) ppm. ^13^C NMR (75 MHz, CDCl_3_): δ = 57.0 (C-1”), 76.3 (C-3”), 78.0 (C-2”), 111.0 (C-3), 114.1 (d, C-6’, *J* 23.7 Hz), 114.5 (d, C-3′, *J* 8.2 Hz), 117.0 (d, C-4’, *J* 23.3 Hz), 118.0 (C-8), 122.3 (C-α), 124.2 (C-4a), 125.1 (C-6), 125.7 (C-5), 126.3 (d, C-1’, *J* 7.7 Hz), 130.8 (C-β), 133.8 (C-7), 152.1 (d, C-2’, *J* 2.3 Hz), 156.0 (C-8a), 157.7 (d, C-5′, *J* 240.7 Hz), 161.7 (C-2), 178.5 (C-4) ppm. ^19^F NMR (282 MHz, CDCl_3_): δ −118.38 – −118.30 (m) ppm. HRMS (ESI^+^): *m*/*z* [M + H]^+^ calcd for C_20_H_14_O_3_F 321.0921; found 321.0911.

#### (*E*)-2-[5-Chloro-2-(prop-2-yn-1-yloxy)­styryl]-4*H*-chromen-4-one (**7j**)

yellow solid; yield 180 mg (45%); 162–164 °C. ^1^H NMR (300 MHz, CDCl_3_): δ = 2.59 (t, 1H, H-3”, *J* 2.4 Hz), 4.83 (d, 2H, H-1”, *J* 2.4 Hz), 6.36 (s, 1H, H-3), 6.88 (d, 1H, H-α, *J* 16.2 Hz), 7.03 (d, 1H, H-3′, *J* 8.8 Hz), 7.31 (dd, 1H, H-4’, *J* 8.8, 2.6 Hz), 7.40 (ddd, 1H, H-6, *J* 8.0, 7.0, 1.7 Hz), 7.54–7.59 (m, 2H, H-8, H-6’), 7.69 (ddd, 1H, H-7, *J* 8.5, 7.0, 1.7 Hz), 7.84 (d, 1H, H-β, *J* 16.2 Hz), 8.20 (dd, 1H, H-5, *J* 8.0, 1.7 Hz) ppm. ^13^C NMR (75 MHz, CDCl_3_): δ = 56.6 (C-1”), 76.5 (C-3”), 77.7 (C-2”), 111.0 (C-3), 114.2 (C-3′), 118.0 (C-8 or C-6’), 122.4 (C-α), 124.2 (C-4a), 125.1 (C-6), 125.7 (C-5), 126.3 (C-1’), 127.1 (C-5′), 127.8 (C-8 or C-6’), 130.2 (C-4’), 130.5 (C-β), 133.8 (C-7), 154.3 (C-2’), 156.0 (C-8a), 161.7 (C-2), 178.6 (C-4) ppm. HRMS (ESI^+^): *m*/*z* [M + H]^+^ calcd for C_20_H_14_O_3_Cl 337.0626; found 337.0614.

#### (*E*)-2-[3-Bromo-5-chloro-2-(prop-2-yn-1-yloxy)­styryl]-4*H*-chromen-4-one (**7k**)

yellow solid; yield 291 mg (58%); m.p 186–189 °C. ^1^H NMR (300 MHz, CDCl_3_): δ = 2.58 (t, 1H, H-3”, *J* 2.4 Hz), 4.77 (d, 2H, H-1”, *J* 2.5 Hz), 6.39 (s, 1H, H-3), 6.86 (d, 1H, H-α, *J* 16.2 Hz), 7.42 (ddd, 1H, H-6, J 8.0, 7.1, 1.1 Hz), 7.55 (dd, 1H, H-8, *J* 8.6, 1.1 Hz), 7.58–7.61 (m, 2H, H-4’, H-6’), 7.71 (ddd, 1H, H-7, *J* 8.6, 7.1, 1.7 Hz), 7.95 (d, 1H, H-β, *J* 16.2 Hz), 8.20 (dd, 1H, H-5, *J* 8.0, 1.7 Hz) ppm. ^13^C NMR (75 MHz, CDCl_3_): δ = 61.6 (C-1”), 77.2 (C-3”), 77.8 (C- 2”), 111.6 (C-3), 117.9 (C-8), 119.2 (C-3′), 123.6 (C-α), 124.1 (C-4a), 125.2 (C-6), 125.8 (C-5), 126.2 (C-4’ or C-6’), 130.3 (C-β), 131.2 (C-5′), 132.5 (C-1’), 133.8 (C-4’ or C-6’), 134.0 (C-7), 152.4 (C-2’), 156.0 (C-8a), 161.0 (C-2), 178.5 (C-4) ppm. HRMS (ESI^+^): *m*/*z* [M + H]^+^ calcd for C_20_H_13_O_3_BrCl 414.9731; found 414.9715.

#### (*E*)-2-{2-[2-(4-oxo-4*H*-chromen-2-yl)­vinyl]­phenoxy}­acetonitrile (**7l**)

yellow solid; yield 182 mg (60%). Both spectroscopic and analytic data are in accordance with those previously reported.^25^

#### (*E*)-2-{2-[(3-Phenylprop-2-yn-1-yl)­oxy]­styryl}-4*H*-chromen-4-one (**7m**)

yellow solid; yield 262 mg (58%); m.p 159–160 °C. ^1^H NMR (300 MHz, CDCl_3_): δ = 5.07 (s, 2H, H-1”), 6.34 (s, 1H, H-3), 6.91 (d, 1H, H-α, *J* 16.2 Hz), 7.07 (dt, 1H, H-5′, *J* 7.6, 1.1 Hz), 7.18 (dd, 1H, H-3′, *J* 8.3, 1.1 Hz), 7.30–7.46 (m, 7H, H-6, H-4’, H-2”’, H-3”’, H-4”’, H-5”’, H-6”’), 7.55 (dd, 1H, H-8, *J* 8.5, 1.2 Hz), 7.62–7.69 (m, 2H, H-7, H-6’), 7.99 (d, 1H, H-β, *J* 16.2 Hz), 8.20 (dd, 1H, H-5, *J* 7.9, 1.7 Hz) ppm. ^13^C NMR (125 MHz, CDCl_3_): δ = 57.4 (C-1”), 83.5 (C-3”), 87.8 (C-2”), 110.5 (C-3), 113.3 (C-3′), 118.0 (C-8), 121.1 (C-α), 121.8 (C-5′), 124.1 (C-4a), 124.8 (C-1’), 124.9 (C-6), 125.7 (C-5), 128.2 (C-6’), 128.4 (C-3”’ and C-5”’), 128.9 (C-4”’), 130.9 (C-1”’, C-4’), 131.8 (C-2”’ and C-6”’), 132.2 (C-β), 133.6 (C-7), 156.1 (C-8a), 156.3 (C-2’), 162.4 (C-2), 178.6 (C-4) ppm. HRMS (ESI^+^): *m*/*z* [M^+^H]^+^ calcd for C_26_H_19_O_3_ 379.1329; found 379.1317.

#### (*E*)-2-(2-{[3-(4-Methoxyphenyl)­prop-2-yn-1-yl]­oxy}­styryl)-4*H*-chromen-4-one (**7n**)

yellow solid; yield 121 mg (25%); m.p 135–138 °C. ^1^H NMR (300 MHz, CDCl_3_): δ = 3.80 (s, 3H, 4”’-OC*H*_3_), 5.05 (s, 2H, H-1”), 6.37 (s, 1H, H-3), 6.82 (d, 2H, H-3”’, H-5”’, *J* 8.9 Hz), 6.91 (d, 1H, H-α, *J* 16.2 Hz), 7.06 (td, 1H, H-5′, *J* 7.5, 1.1 Hz), 7.18 (dd, 1H, H-3′, *J* 8.2, 1.1 Hz), 7.36–7.41 (m, 4H, H-6, H-4’, H-2”’, H-6”’), 7.54 (dd, 1H, H-8, *J* 8.5, 1.1 Hz), 7.61–7.70 (m, 2H, H-7, H-6’), 7.99 (d, 1H, H-β, *J* 16.2 Hz), 8.20 (dd, 1H, H-5, *J* 7.9, 1.7 Hz) ppm. ^13^C NMR (75 MHz, CDCl_3_): δ = 55.3 (4”’-O*C*H_3_), 57.5 (C-1”), 82.2 (C-3”), 87.8 (C-2”), 110.3 (C-3), 113.3 (C-3′), 114.0 (C-3”’ and C-5”’), 114.1 (C-1”’), 118.0 (C-8), 121.0 (C-α), 121.7 (C-5′), 124.1 (C-4a), 124.8 (C-1’), 124.9 (C-6 or C-4’), 125.6 (C-5), 128.2 (C-6’), 130.9 (C-6 or C-4’), 132.3 (C-β), 133.4 (C-2”’ and C-6”’), 133.6 (C-7), 156.1 (C-8a), 156.4 (C-2’), 160.0 (C-4”’), 162.6 (C-2), 178.6 (C-4) ppm. HRMS (ESI^+^): *m*/*z* [M^+^H]^+^ calcd for C_27_H_21_O_4_ 409.1434; found 409.1422.

#### (*E*)-2-(2-{[3-(4-methylphenyl)­prop-2-yn-1-yl]­oxy}­styryl)-4*H*-chromen-4-one (**7o**)

yellow solid; yield 327 mg (67%). Both spectroscopic and analytic data are in accordance with those previously reported.^25^

#### (*E*)-7-Methoxy-2-[5-methoxy-2-(prop-2-yn-1-yloxy)­styryl]-4*H*-chromen-4-one (**7p**)

yellow solid; yield 184 mg (51%). Both spectroscopic and analytic data are in accordance with those previously reported.^25^

#### (*E*)-5-Methoxy-2-[5-methoxy-2-(prop-2-yn-1-yloxy)­styryl]­naphthalen-1­(4*H*)-one one (**7q**)

yellow solid; yield 178 mg (49%). Both spectroscopic and analytic data are in accordance with those previously reported.^25^

#### (*E*)-7,8-Dimethoxy-2-[5-methoxy-2-(prop-2-yn-1-yloxy)­styryl]­chroman-4-one (**7r**)

yellow solid; yield 156 mg (40%). Both spectroscopic and analytic data are in accordance with those previously reported.^25^

#### (*E*)-5,7-Dimethoxy-2-[5-methoxy-2-(prop-2-yn-1-yloxy)­styryl]-4*H*-chromen-4-one (**7s**)

yellow solid; yield 93 mg (26%); m.p 187–189 °C. ^1^H NMR (300 MHz, CDCl_3_): δ = 2.56 (t, 1H, H-3”, *J* 2.4 Hz), 3.83 (s, 3H, 5′-OC*H*_3_), 3.93 (s, 3H, 7-OC*H*_3_), 3.95 (s, 3H, 5-OC*H*_3_), 4.77 (d, 2H, H-1”, *J* 2.4 Hz), 6.20 (s, 1H, H-3), 6.36 (d, 1H, H-6, *J* 2.3 Hz), 6.58 (d, 1H, H-8, *J* 2.3 Hz), 6.77 (d, 1H, H-α, *J* 16.2 Hz), 6.90 (d, 1H, H-4’, *J* 9.0, 3.0 Hz), 7.03 (d, 1H, H-3′, *J* 9.0 Hz), 7.10 (d, 1H, H-6’, *J* 3.0 Hz), 7.78 (d, 1H, H-β, *J* 16.2 Hz) ppm. ^13^C NMR (75 MHz, CDCl_3_): δ = 55.8 (7-O*C*H_3_), 56.4 (5′-O*C*H_3_), 57.2 (5-O*C*H_3_), 57.2 (C-1”), 75.9 (C-3”), 78.6 (C-2”), 92.9 (C-8), 96.0 (C-6), 109.5 (C-4a), 112.3 (C-3), 112.6 (C-6’), 114.8 (C-3′), 116.0 (C-4’), 121.2 (C-α), 125.9 (C-1’), 130.6 (C-β), 150.2 (C-2’), 154.5 (C-5′), 159.5 (C-2), 159.7 (C-8a), 160.9 (C-5), 164.0 (C-7), 177.8 (C-4) ppm. HRMS (ESI^+^): *m*/*z* [M + H]^+^ calcd for C_23_H_21_O_6_ 393.1333; found 393.1318.

#### (*E*)-7-Methoxy-2-[2-(prop-2-yn-1-yloxy)-5-(trifluoromethoxy)­styryl]-4*H*-chromen-4-one (**7t**)

yellow solid; yield 130 mg (31%); m.p 168–169 °C. ^1^H NMR (300 MHz, CDCl_3_): δ = 2.61 (t, 1H, H-3”, *J* 2.4 Hz), 3.95 (s, 3H, 7-OC*H*_3_), 4.85 (d, 2H, H-1”, *J* 2.4 Hz), 6.30 (s, 1H, H-3), 6.87 (d, 1H, H-α, *J* 16.2 Hz), 6.95–6.99 (m, 2H, H-6, H-8), 7.09 (d, 1H, H-3′, *J* 9.0 Hz), 7.22 (dd, 1H, H-4’, *J* 9.0, 2.4 Hz), 7.45 (d, 1H, H-6’, *J* 2.4 Hz), 7.80 (d, 1H, H-β, *J* 16.2 Hz), 8.20 (d, 1H, H-5, *J* 9.5 Hz) ppm. ^13^C NMR (75 MHz, CDCl_3_): δ = 55.9 (7-O*C*H_3_), 56.7 (C-1”), 77.2 (C-3”), 77.7 (C-2”), 100.4 (C-8), 111.2 (C-3), 113.8 (C-3), 114.2 (C-6), 118.1 (C-4a), 120.5 (q, 5′-O*C*F_3_, *J* 257.0 Hz), 120.9 (C-6’), 122.9 (C-α), 123.0 (C-4’), 126.1 (C-1’), 127.1 (C-5), 129.9 (C-β), 143.5 (C-5′), 154.1 (C-2’), 157.8 (C-8a), 161.2 (C-2), 164.3 (C-7), 177.9 (C-4) ppm. ^19^F NMR (282 MHz, CDCl_3_): δ −54.86 (s) ppm. HRMS (ESI^+^): *m*/*z* [M + H]^+^ calcd for C_22_H_16_O_5_F_3_ 417.0944; found 417.0935.

#### (*E*)-2-[5-Fluoro-2-(prop-2-yn-1-yloxy)­styryl]-7-methoxy-4*H*-chromen-4-one (**7u**)

yellow solid; yield 170 mg (48%); m.p 177–178 °C. ^1^H NMR (300 MHz, CDCl_3_): δ = 2.58 (t, 1H, H-3”, *J* 2.4 Hz), 3.95 (s, 3H, 7-OC*H*_3_), 4.82 (d, 2H, H-1”, *J* 2.4 Hz), 6.30 (s, 1H, H-3), 6.83 (d, 1H, H-α, *J* 16.2 Hz), 6.95–6.99 (m, 2H, H-6, H-8), 7.03–7.07 (m, 2H, H-3′, H-4’), 7.31 (dd, 1H, H-6’, *J* 8.9, 2.3 Hz), 7.82 (d, 1H, H-β, *J* 16.2 Hz), 8.10 (d, 1H, H-5, *J* 9.5 Hz) ppm. ^13^C NMR (125 MHz, CDCl_3_): δ = 55.9 (7-O*C*H_3_), 57.0 (C-1”), 76.3 (C-3”), 78.0 (C-2”), 100.4 (C-8), 111.1 (C-3), 114.0 (d, C-6’, *J* 23.6 Hz), 114.2 (C-6), 114.4 (d, C-3′, *J* 8.2 Hz), 116.9 (d, C-4’, *J* 23.6 Hz), 118.0 (C-4a), 122.4 (C-α), 126.3 (d, C-1’, *J* 7.6 Hz), 127.1 (C-5), 130.1 (C-β), 152.0 (C-2’), 157.7 (d, C-5′, *J* 240.7 Hz), 157.8 (C-8a), 161.3 (C-2), 164.2 (C-7), 178.0 (C-4) ppm. ^19^F NMR (282 MHz, CDCl_3_): δ −118.41 – −118.36 (m) ppm. HRMS (ESI^+^): *m*/*z* [M + H]^+^ calcd for C_21_H_16_O_4_F: 351.1027; found 351.1014.

#### (*E*)-2-[5-Chloro-2-(prop-2-yn-1-yloxy)­styryl]-7-methoxy-4*H*-chromen-4-one (**7v**)

yellow solid; yield 124 mg (33%); m.p 188–189 °C. ^1^H NMR (300 MHz, CDCl_3_): δ = 2.59 (t, 1H, H-3”, *J* 2.4 Hz), 3.94 (s, 3H, 7-OC*H*_3_), 4.83 (d, 2H, H-1”, *J* 2.4 Hz), 6.29 (s, 1H, H-3), 6.85 (d, 1H, H-α, *J* 16.2 Hz), 6.95–6.99 (m, 2H, H-6, H-8), 7.02 (d, 1H, H-3′, *J* 8.9 Hz), 7.31 (dd, 1H, H-4’, *J* 8.9, 2.6 Hz), 7.57 (d, 1H, H-6’, *J* 2.6 Hz), 7.79 (d, 1H, H-β, *J* 16.2 Hz), 8.10 (d, 1H, H-5, *J* 9.2 Hz) ppm. ^13^C NMR (75 MHz, CDCl_3_): δ = 55.9 (7-O*C*H_3_), 56.6 (C-1”), 76.5 (C-3”), 77.8 (C-2”), 100.4 (C-6 or C-8), 111.1 (C-3), 114.2 (C-6 or C-8 and C-3′), 118.0 (C-4a), 122.5 (C-α), 126.4 (C-1’), 127.1 (C-5), 127.7 (C-6’), 129.9 (C-β), 130.1 (C-4’), 154.3 (C-2’), 157.8 (C-8a), 161.3 (C-2), 164.2 (C-7), 177.9 (C-4) ppm. HRMS (ESI^+^): *m*/*z* [M + H]^+^ calcd for C_21_H_16_O_4_Cl 367.0732; found 367.0720.

#### (*E*)-2-[3-Bromo-5-chloro-2-(prop-2-yn-1-yloxy)­styryl]-7-methoxy-4*H*-chromen-4-one (**7w**)

yellow solid; yield 90 mg (20%); m.p 196–198 °C. ^1^H NMR (300 MHz, CDCl_3_): δ = 2.57 (t, 1H, H-3”, *J* 2.4 Hz), 3.95 (s, 3H, 7-OC*H*_3_), 4.76 (d, 2H, H-1”, *J* 2.4 Hz), 6.32 (s, 1H, H-3), 6.84 (d, 1H, H-α, *J* 16.2 Hz), 6.95 (d, 1H, H-8, *J* 2.4 Hz), 6.98 (dd, 1H, H-6, *J* 8.8, 2.4 Hz), 7.58 (d, 2H, H-4’, H-6’), 7.89 (d, 1H, H-β, *J* 16.2 Hz), 8.11 (d, 1H, H-5, *J* 8.8 Hz) ppm. ^13^C NMR (75 MHz, CDCl_3_): δ = 55.9 (7-O*C*H_3_), 61.6 (C-1”), 77.2 (C-3”), 77.9 (C-2”), 100.5 (C-8), 111.7 (C-3), 114.2 (C-6), 118.0 (C-4a), 118.9 (C-3′ or C-5′), 123.7 (C-α), 126.2 (C-6’), 127.2 (C-5), 129.7 (C-β), 131.2 (C-3′ or C-5′), 132.5 (C-1’), 133.7 (C-4’), 152.3 (C-2’), 157.8 (C-8a), 160.6 (C-2), 164.4 (C-7), 177.8 (C-4) ppm. HRMS (ESI^+^): *m*/*z* [M + H]^+^ calcd for C_21_H_15_O_4_BrCl 444.9837; found 444.9820.

#### (*E*)-2-{2-[3-(Prop-2-yn-1-yloxy)­naphthalen-2-yl]­vinyl}-4*H*-chromen-4-one (**8a**)

yellow solid; yield 271 mg (64%); m.p 150–152 °C. ^1^H NMR (300 MHz, CDCl_3_): δ = 2.59 (t, 1H, H-3”, *J* 2.4 Hz), 4.93 (d, 2H, H-1”, *J* 2.4 Hz), 6.42 (s, 1H, H-3), 7.16 (d, 1H, H-α, *J* 16.2 Hz), 7.41–7.47 (m, 3H, H-6, H-3′, H-6’), 7.59 (ddd, 1H, H-7’, *J* 8.5, 6.7, 1.4 Hz), 7.62 (d, 1H, H-8, *J* 8.5 Hz), 7.72 (ddd, 1H, H-7, *J* 8.5, 7.0, 1.7 Hz), 7.85 (dd, 1H, H-5′, *J* 8.2, 1.4 Hz), 7.89 (d, 1H, H-4’, *J* 9.1 Hz), 8.21–8.24 (m, 3H, H-β, H-5, H-8’) ppm. ^13^C NMR (75 MHz, CDCl_3_): δ = 57.1 (C-1”), 76.3 (C-3”), 78.3 (C-2”), 110.6 (C-3), 114.4 (C-3′), 118.1 (C-8), 118.8 (C-1’), 123.5 (C-5 or C-8’), 124.5 (C-4a), 125.1 (C-6 and C-6’), 125.7 (C-5 or C-8’), 126.3 (C-α), 127.5 (C-7’), 128.8 (C-5′), 129.7 (C-4a’), 130.1 (C-β), 131.1 (C-4’) 132.6 (C-8a’), 133.8 (C-7), 154.2 (C-2’), 156.2 (C-8a), 162.6 (C-2), 178.7 (C-4) ppm. HRMS (ESI^+^): *m*/*z* [M + H]^+^ calcd for C_24_H_17_O_3_: 353.1172; found 353.1162.

#### (*E*)-7-Methoxy-2-{2-[3-(prop-2-yn-1-yloxy)­naphthalen-2-yl]­vinyl}-4*H*-chromen-4-one (**8b**)

yellow solid; yield 123 mg (32%); m.p 164–167 °C. ^1^H NMR (500 MHz, CDCl_3_): δ = 2.58 (t, 1H, H-3”, *J* 2.4 Hz), 3.97 (s, 3H, 7-OC*H*_3_), 4.93 (d, 2H, H-1”, *J* 2.3 Hz), 6.34 (s, 1H, H-3), 6.99 (dd, 1H, H-6, *J* 8.8, 2.4 Hz), 7.02 (d, 1H, H-8, *J* 2.4 Hz), 7.12 (d, 1H, H-α, *J* 16.2 Hz), 7.41–7.46 (m, 2H, H-3′, H-6’), 7.59 (ddd, 1H, H-7’, *J* 8.6, 6.8, 1.4 Hz), 7.85 (dd, 1H, H-5′, *J* 8.2, 1.4 Hz), 7.88 (d, 1H, H-4’, *J* 9.1 Hz), 8.13–8.18 (m, 2H, H-β, H-5), 8.23 (dd, 1H, H-8’, *J* 8.6, 1.0 Hz) ppm. ^13^C NMR (125 MHz, CDCl_3_): δ = 55.9 (7-O*C*H_3_), 57.1 (C-1”), 76.3 (C-3”), 78.4 (C-2”), 100.4 (C-8), 110.7 (C-3), 114.2 (C-6), 114.4 (C-3′), 118.1 (C-4a), 118.9 (C-1’), 123.5 (C-8’), 124.5 (C-6’), 126.5 (C-α), 127.1 (C-5 or C-β), 127.5 (C-7’), 128.8 (C-5′), 129.4 (C-5 or C-β), 129.7 (C-4a’), 130.9 (C-4’), 132.6 (C-8a’), 154.1 (C-2’), 157.9 (C-8a), 162.1 (C-2), 164.2 (C-7), 178.1 (C-4) ppm. HRMS (ESI^+^): *m*/*z* [M + H]^+^ calcd for C_25_H_19_O_6_ 383.1278; found 383.1264.

#### (*E*)-2-{2-[Di­(prop-2-yn-1-yl)­amino]­styryl}-4*H*-chromen-4-one (**10**)

yellow solid; yield 278 mg (82%). Both spectroscopic and analytic data are in accordance with those previously reported.^25^

#### General Procedure for the Synthesis of Compounds **11a-s** and **12a-b**

The appropriate (*E*)-2’-propargyloxy-2-styrylchromones **7a**–**k** and **7p**–**w** or **8a**–**b** (0.1 mmol) and 1,2,4-TCB (1 mL) were mixed in a closed glass vessel. The resulting mixture was heated under MW radiation at 220 °C for 30 min. After that period, chloranil (10 μmol, 2.5 mg) was added and the reaction mixture heated under MW radiation at 80 °C for additional 30 min. The 1,2,4-TCB was removed from the reaction slurry using silica-gel column chromatography eluted with hexane (5 mL), concentrated under vacuum, and then purified by preparative TLC, using DCM as eluent.

#### 6*H*,8*H*-Chromeno­[3,4-*b*]­xanthen-8-one (**11a**)

yellow solid; yield 23 mg (75%). Both spectroscopic and analytic data are in accordance with those previously reported.^53^

#### 11-Methoxy-6*H*,8*H*-chromeno­[3,4-*b*]­xanthen-8-one (**11b**)

yellow solid; yield 26 mg (79%). Both spectroscopic and analytic data are in accordance with those previously reported.^53^

#### 9-Methoxy-6*H*,8*H*-chromeno­[3,4-*b*]­xanthen-8-one (**11c**)

yellow solid; yield 22 mg (67%). Both spectroscopic and analytic data are in accordance with those previously reported.^53^

#### 11,12-Dimethoxy-6*H*,8*H*-chromeno­[3,4-*b*]­xanthen-8-one (**11d**)

yellow solid; yield 30 mg (83%). Both spectroscopic and analytic data are in accordance with those previously reported.^25^

#### 9,11-Dimethoxy-6*H*,8*H*-chromeno­[3,4-*b*]­xanthen-8-one (**11e**)

yellow solid; yield 16 mg (40%). Both spectroscopic and analytic data are in accordance with those previously reported.^53^

#### 2-Methoxy-6*H*,8*H*-chromeno­[3,4-*b*]­xanthen-8-one (**11f**)

yellow solid; yield 17 mg (51%). Both spectroscopic and analytic data are in accordance with those previously reported.^25^

#### 2-Bromo-6*H*,8*H*-chromeno­[3,4-*b*]­xanthen-8-one (11g)

yellow solid; yield 20 mg (42%). Both spectroscopic and analytic data are in accordance with those previously reported.^25^

#### 2-(Trifluoromethoxy)-6*H*,8*H*-chromeno­[3,4-*b*]­xanthen-8-one (**11h**)

yellow solid; yield 35 mg (69%); m.p 215–217 °C. ^1^H NMR (500 MHz, CDCl_3_): δ = 5.24 (s, 2H, H-6), 7.06 (d, 1H, H-4, *J* 8.9 Hz), 7.20 (dd, 1H, H-3, *J* 8.9, 2.4 Hz), 7.40 (ddd, 1H, H-10, *J* 8.1, 7.1, 1.1 Hz), 7.51 (dd, 1H, H-12, *J* 8.6, 1.1 Hz), 7.64 (d, 1H, H-1, *J* 2.4 Hz), 7.72 (s, 1H, H-14), 7.75 (ddd, 1H, H-11, *J* 8.6, 7.1, 1.7 Hz), 8.13 (s, 1H, H-7), 8.33 (dd, 1H, H-9, *J* 8.1, 1.7 Hz) ppm. ^13^C NMR (125 MHz, CDCl_3_): δ = 68.1 (C-6), 111.4 (C-14), 117.2 (C-1), 118.0 (C-12), 119.3 (C-4), 120.6 (q, 2-O*C*F_3_, *J* 257.0 Hz), 121.3 (C-7a), 121.8 (C-8a), 122.4 (C-14b), 122.9 (C-7), 124.3 (C-3 and C-10), 126.8 (C-9), 127.3 (C-6a), 135.1 (C-11), 135.7 (C-14a), 144.0 (C-2), 154.0 (C-4a), 156.2 (C-12a), 156.4 (C-13a), 176.5 (C-8) ppm. ^19^F NMR (282 MHz, CDCl_3_): δ −54.87 (s) ppm. HRMS (ESI^+^): *m*/*z* [M^+^H]^+^ calcd for C_21_H_12_O_4_F_3_ 385.0682; found 385.0663.

#### 2-Fluoro-6*H*,8*H*-chromeno­[3,4-*b*]­xanthen-8-one (**11i**)

yellow solid; yield 68 mg (76%); m.p 225–227 °C. ^1^H NMR (500 MHz, CDCl_3_): δ = 5.21 (s, 2H, H-6), 6.98–7.09 (m, 2H, H-1, H-4), 7.40 (ddd, 1H, H-10, *J* 8.0, 7.1, 1.1 Hz), 7.46–7.54 (m, 2H, H-3, H-12), 7.70 (s, 1H, H-14), 7.75 (ddd, 1H, H-11, *J* 8.7, 7.1, 1.8 Hz), 8.13 (s, 1H, H-7), 8.34 (dd, 1H, H-9, *J* 8.0, 1.8 Hz) ppm. ^13^C NMR (125 MHz, CDCl_3_): δ = 68.1 (C-6), 110.5 (d, C-3, *J* 24.4 Hz), 111.3 (C-14), 118.0 (C-12), 118.2 (d, C-1, *J* 23.7 Hz), 119.3 (d, C-4, *J* 8.1 Hz), 121.2 (C-7a), 121.9 (C-8a), 122.5 (d, C-14b, *J* 8.1 Hz), 122.8 (C-7), 124.2 (C-10), 126.8 (C-9), 127.6 (C-6a), 135.0 (C-11), 136.2 (C-14a), 151.7 (C-4a), 156.3 (C-12a), 156.4 (C-13a), 158.2 (d, C-2, *J* 240.7 Hz), 176.6 (C-8) ppm. ^19^F NMR (282 MHz, CDCl_3_): δ −117.26 – −117.20 (m) ppm. HRMS (ESI^+^): *m*/*z* [M^+^H]^+^ calcd for C_20_H_12_O_3_F 319.0765; found 319.0765.

#### 2-Chloro-6*H*,8*H*-chromeno­[3,4-*b*]­xanthen-8-one (**11j**)

yellow solid; yield 20 mg (40%); m.p 229–232 °C. ^1^H NMR (300 MHz, CDCl_3_): δ = 5.23 (s, 2H, H-6), 7.00 (d, 1H, H-4, *J* 8.7 Hz), 7.30 (dd, 1H, H-3, *J* 8.7, 2.5 Hz), 7.41 (ddd, 1H, H-10, *J* 8.0, 7.1, 1.5 Hz), 7.52 (dd, 1H, H-12, *J* 8.5, 1.5 Hz), 7.73–7.79 (m, 3H, H-1, H-11, H-14), 8.14 (s, 1H, H-7), 8.35 (dd, 1H, H-9, *J* 8.0, 1.7 Hz) ppm. ^13^C NMR (75 MHz, CDCl_3_): δ = 68.1 (C-6), 111.2 (C-14), 118.0 (C-12), 119.5 (C-4), 121.7 (C-7a), 121.9 (C-8a), 122.8 (C-14b), 122.9 (C-7), 124.1 (C-10), 124.2 (C-1), 126.8 (C-9), 127.3 (C-2), 127.6 (C-6a), 131.2 (C-3), 135.0 (C-11), 135.8 (C-14a), 154.2 (C-4a), 156.3 (C-12a), 156.5 (C-13a), 176.6 (C-8) ppm. HRMS (ESI^+^): *m*/*z* [M^+^H]^+^ calcd for C_20_H_12_O_3_Cl 335.0469; found 335.0455.

#### 4-Bromo-2-chloro-6*H*,8*H*-chromeno­[3,4-*b*]­xanthen-8-one (**11k**)

yellow solid; yield 10 mg (20%); m.p 245–246 °C. ^1^H NMR (300 MHz, CDCl_3_): δ = 5.31 (s, 2H, H-6), 7.40 (ddd, 1H, H-10, *J* 8.0, 7.1, 1.1 Hz), 7.50 (dd, 1H, H-12, *J* 8.6, 1.1 Hz), 7.56 (d, 1H, H-3, *J* 2.4 Hz), 7.70–7.71 (m, 2H, H-1, H-14), 7.75 (ddd, 1H, H-11, *J* 8.6, 7.1, 1.8 Hz), 8.13 (s, 1H, H-7), 8.32 (dd, 1H, H-9, *J* 8.0, 1.8 Hz) ppm. ^13^C NMR (75 MHz, CDCl_3_): δ = 68.6 (C-6), 111.7 (C-14 or C-1), 112.8 (C-4), 118.0 (C-12), 121.6 (C-7a), 121.8 (C-8a), 123.0 (C-7), 123.4 (C-14 or C-1), 123.6 (C-14b), 124.3 (C-10), 126.8 (C-9), 126.9 (C-6a), 127.7 (C-2), 133.9 (C-3), 135.0 (C-14a), 135.1 (C-11), 151.0 (C-4a), 156.2 (C-12a), 156.4 (C-13a), 176.4 (C-8) ppm. HRMS (ESI^+^): *m*/*z* [M^+^H]^+^ calcd for C_20_H_11_O_3_BrCl 412.9575; found 412.9555.

#### 2,11-Dimethoxy-6*H*,8*H*-chromeno­[3,4-*b*]­xanthen-8-one (**11l**)

yellow solid; yield 22 mg (61%). Both spectroscopic and analytic data are in accordance with those previously reported.^25^

#### 2,9-Dimethoxy-6*H*,8*H*-chromeno­[3,4-*b*]­xanthen-8-one (**11m**)

yellow solid; yield 19 mg (53%). Both spectroscopic and analytic data are in accordance with those previously reported.^25^

#### 2,11,12-Trimethoxy-6*H*,8*H*-chromeno­[3,4-*b*]­xanthen-8-one (**11n**)

yellow solid; yield 18 mg (45%). Both spectroscopic and analytic data are in accordance with those previously reported.^25^

#### 2,9,11-Trimethoxy-6*H*,8*H*-chromeno­[3,4-*b*]­xanthen-8-one (**11o**)

yellow solid; yield 38 mg (75%); m.p °C. ^1^H NMR (300 MHz, CDCl_3_): δ = 3.87 (s, 3H, 2-OC*H*_3_), 3.93 (s, 3H, 11-OC*H*_3_), 3.99 (s, 3H, 9-OC*H*_3_), 5.15 (s, 2H, H-6), 6.35 (d, 1H, H-10, *J* 2.4 Hz), 6.51 (d, 1H, H-12, *J* 2.4 Hz), 6.91 (dd, 1H, H-3, *J* 8.9, 2.8 Hz), 6.98 (d, 1H, H-4, *J* 8.9 Hz), 7.60 (s, 1H, H-14), 8.06 (s, 1H, H-7) ppm. ^13^C NMR (75 MHz, CDCl_3_): δ = 55.8 (11-O*C*H_3_), 55.9 (2-O*C*H_3_), 56.4 (9-O*C*H_3_), 68.2 (C-6), 92.9 (C-12), 95.2 (C-10), 107.3 (C-8a), 110.1 (C-14), 117.1 (C-3), 118.7 (C-4), 121.3 (C-14b), 122.1 (C-7a), 122.7 (C-7), 127.8 (C-6a), 136.0 (C-14a), 149.7 (C-4a), 154.9 (C-2), 155.2 (C-13a), 159.9 (C-12a), 162.1 (C-9), 165.0 (C-11), 174.9 (C-8) ppm. HRMS (ESI^+^): *m*/*z* [M^+^H]^+^ calcd for C_23_H_19_O_6_ 391.1176; found 391.1162.

#### 11-Methoxy-2-(trifluoromethoxy)-6*H*,8*H*-chromeno­[3,4-*b*]­xanthen-8-one (**11p**)

yellow solid; yield 36 mg (72%); m.p 198–199 °C. ^1^H NMR (500 MHz, CDCl_3_): δ = 3.95 (s, 3H, 11-OC*H*_3_), 5.22 (s, 2H, H-6), 6.89 (d, 1H, H-12, *J* 2.4 Hz), 6.95 (dd, 1H, H-10, *J* 8.9, 2.4 Hz), 7.05 (d, 1H, H-4, *J* 8.9 Hz), 7.19 (dd, 1H, H-3, *J* 8.9, 2.3 Hz), 7.62 (d, 1H, H-1, *J* 2.3 Hz), 7.66 (s, 1H, H-14), 8.10 (s, 1H, H-7), 8.23 (d, 1H, H-9, *J* 8.9 Hz) ppm. ^13^C NMR (125 MHz, CDCl_3_): δ = 55.9 (11-O*C*H_3_), 68.1 (C-6), 100.3 (C-12), 111.1 (C-14), 113.5 (C-10), 115.8 (C-8a), 117.1 (C-1), 119.2 (C-4), 120.6 (q, 2-O*C*F_3_, *J* 256.6 Hz), 121.4 (C-14b), 122.5 (C-7a), 122.9 (C-7), 124.1 (C-3), 127.2 (C-6a), 128.3 (C-9), 135.1 (C-14a), 144.0 (C-2), 154.0 (C-4a), 156.4 (C-13a), 158.1 (C-12a), 165.3 (C-11), 175.6 (C-8) ppm. ^19^F NMR (282 MHz, CDCl_3_): δ −54.86 (s) ppm. HRMS (ESI^+^): *m*/*z* [M^+^H]^+^ calcd for C_22_H_14_O_5_F_3_ 415.0788; found 415.0773.

#### 2-Fluoro-11-methoxy-6*H*,8*H*-chromeno­[3,4-*b*]­xanthen-8-one (**11q**)

yellow solid; yield 20 mg (41%); m.p 228–230 °C. ^1^H NMR (500 MHz, CDCl_3_): δ = 3.96 (s, 3H, 11-OC*H*_3_), 5.20 (s, 2H, H-6), 6.90 (d, 1H, H-12, *J* 2.3 Hz), 6.96 (dd, 1H, H-10, *J* 8.9, 2.3 Hz), 6.99–7.06 (m, 2H, H-3, H-4), 7.46 (dd, 1H, H-1, *J* 9.0, 2.3 Hz), 7.65 (s, 1H, H-14), 8.11 (s, 1H, H-7), 8.24 (d, 1H, H-9, *J* 8.9 Hz) ppm. ^13^C NMR (125 MHz, CDCl_3_): δ = 55.9 (11-O*C*H_3_), 68.1 (C-6), 100.3 (C-12), 110.4 (d, C-1, *J* 23.8 Hz), 111.0 (C-14), 113.5 (C-10), 115.8 (C-8a), 118.0 (d, C-3, *J* 23.1 Hz), 119.2 (d, C-4, *J* 8.1 Hz), 121.3 (C-7a), 122.5 (d, C-14b, *J* 8.1 Hz), 122.8 (C-7), 127.5 (C-6a), 128.3 (C-9), 135.6 (C-14a), 151.6 (C-4a), 156.4 (C-13a), 158.1 (C-12a), 158.2 (d, C-2, *J* 240.7 Hz), 165.3 (C-11), 175.7 (C-8) ppm. ^19^F NMR (282 MHz, CDCl_3_): δ −117.35 – −117.32 (m) ppm. HRMS (ESI^+^): *m*/*z* [M^+^H]^+^ calcd for C_21_H_14_O_4_F 349.0871; found 349.0872.

#### 2-Chloro-11-methoxy-6*H*,8*H*-chromeno­[3,4-*b*]­xanthen-8-one (**11r**)

yellow solid; yield 28 mg (55%); m.p 275–277 °C. ^1^H NMR (300 MHz, CDCl_3_): δ = 3.96 (s, 3H, 11-OC*H*_3_), 5.21 (s, 2H, H-6), 6.89 (d, 1H, H-12, *J* 2.4 Hz), 6.94–7.00 (m, 2H, H-10, H-4), 7.27–7.30 (m, 1H, H-3, *overlapped with solvent residual signal*), 7.67 (s, 1H, H-14), 8.10 (s, 1H, H-7), 7.74 (d, 1H, H-1, *J* 2.4 Hz), 8.29 (d, 1H, H-9, *J* 8.9 Hz) ppm. ^13^C NMR (75 MHz, CDCl_3_): δ = 55.9 (11-O*C*H_3_), 68.1 (C-6), 100.3 (C-12), 110.9 (C-14), 113.4 (C-10), 115.8 (C-8a), 119.4 (C-4), 121.3 (C-14b), 122.8 (C-7), 122.9 (C-7a), 124.0 (C-1), 127.2 (C-6a), 127.5 (C-2), 128.3 (C-9), 131.0 (C-3), 135.2 (C-14a), 154.1 (C-4a), 156.4 (C-13a), 158.1 (C-12a), 165.2 (C-11), 175.6 (C-8) ppm. HRMS (ESI^+^): *m*/*z* [M^+^H]^+^ calcd for C_21_H_14_O_4_Cl 365.0575; found 365.056.

#### 4-Bromo-2-chloro-11-methoxy-6*H*,8*H*-chromeno­[3,4-*b*]­xanthen-8-one (**11s**)

yellow solid; yield 17 mg (34%); m.p 265–267 °C. ^1^H NMR (300 MHz, CDCl_3_): δ = 3.97 (s, 3H, 11-OC*H*_3_), 5.32 (s, 2H, H-6), 6.90 (d, 1H, H-12, *J* 2.4 Hz), 6.98 (dd, 1H, H-10, *J* 8.9, 2.4 Hz), 7.57 (d, 1H, H-3, *J* 2.4 Hz), 7.70 (s, 1H, H-14), 7.72 (d, 1H, H-1, *J* 2.4 Hz), 8.14 (s, 1H, H-7), 8.25 (d, 1H, H-9, *J* 8.9 Hz) ppm. ^13^C NMR (75 MHz, CDCl_3_): δ = 55.9 (11-O*C*H_3_), 68.6 (C-6), 100.3 (C-12), 111.5 (C-14), 112.8 (C-4), 113.5 (C-10), 115.8 (C-8a), 121.7 (C-14b), 123.0 (C-7), 123.3 (C-1), 123.8 (C-2), 126.8 (C-6a), 127.7 (C-7a), 128.3 (C-9), 133.8 (C-3), 134.5 (C-14a), 151.1 (C-4a), 156.4 (C-13a), 158.1 (C-12a), 165.3 (C-11), 175.5 (C-8) ppm. HRMS (ESI^+^): *m*/*z* [M^+^H]^+^ calcd for C_21_H_13_O_4_BrCl 442.968; found 442.9659.

#### 7*H*,9*H*-Benzo­[6,7]­chromeno­[3,4-*b*]­xanthen-9-one (**12a**)

yellow solid; yield 31 mg (63%); m.p 245–246 °C. ^1^H NMR (300 MHz, CDCl_3_): δ = 5.18 (d, 2H, H-8), 7.25–7.28 (d, 1H, H-6, *overlapped with solvent residual signal*), 7.42 (ddd, 1H, H-12, *J* 8.1, 7.0, 1.1 Hz), 7.46–7.55 (m, 2H, H-3, H-14), 7.66 (ddd, 1H, H-2, *J* 8.5, 6.9, 1.4 Hz), 7.76 (ddd, 1H, H-13, *J* 8.6, 7.0, 1.7 Hz), 7.85 (d, 1H, H-5, *J* 8.9 Hz), 7.90 (d, 1H, H-4, *J* 8.3 Hz), 8.14 (s, 1H, H-16), 8.28 (s, 1H, H-9), 8.38 (dd, 1H, H-11, *J* 8.1, 1.7 Hz), 8.61 (d, 1H, H-1, *J* 8.5 Hz) ppm. ^13^C NMR (75 MHz, CDCl_3_): δ = 68.8 (C-8), 114.8 (C-16), 116.3 (C-8a), 118.0 (C-14), 118.4 (C-6), 120.1 (C-16b), 121.9 (C-10a),122.9 (C-9), 124.1 (C-12), 124.2 (C-1), 124.5 (C-3), 126.7 (C-11), 127.8 (C-2), 129.0 (C-9a), 129.2 (C-4), 130.6 (C-16c), 130.6 (C-4a), 132.3 (C-5), 134.9 (C-13), 136.8 (C-16a), 155.8 (C-6a), 156.3 (C-14a), 156.6 (15a), 176.7 (C-10) ppm. HRMS (ESI^+^): *m*/*z* [M^+^H]^+^ calcd for C_24_H_15_O_3_ 351.1016; found 351.1002.

#### 12-Methoxy-7*H*,9*H*-benzo­[6,7]­chromeno­[3,4-*b*]­xanthen-9-one (**12b**)

yellow solid; yield 13 mg (28%); m.p 249–250 °C. ^1^H NMR (300 MHz, CDCl_3_): δ = 3.94 (s, 3H, 13-OC*H*_3_), 5.15 (d, 2H, H-8), 6.91 (d, 1H, H-14, *J* 2.4 Hz), 6.96 (dd, 1H, H-12, *J* 8.9, 2.4 Hz), 7.24–7.27 (d, 1H, H-6, *overlapped with solvent residual signal*), 7.47 (ddd, 1H, H-3, *J* 8.1, 6.9, 1.1 Hz), 7.63 (ddd, 1H, H-2, *J* 8.6, 6.9, 1.4 Hz), 7.83 (d, 1H, H-5, *J* 8.8 Hz), 7.88 (dd, 1H, H-4, *J* 8.1, 1.4 Hz), 8.07 (s, 1H, H-16), 8.23 (s, 1H, H-9), 8.28 (d, 1H, H-11, *J* 8.9 Hz), 8.59 (d, 1H, H-1, *J* 8.6 Hz) ppm. ^13^C NMR (75 MHz, CDCl_3_): δ = 55.9 (13-O*C*H_3_), 68.8 (C-8), 100.3 (C-14), 113.3 (C-12), 114.6 (C-16), 115.9 (C-10a), 116.3 (C-8a), 118.4 (C-6), 120.2 (C-16b), 122.8 (C-9), 124.2 (C-1), 124.4 (C-3), 127.7 (C-2), 128.3 (C-11), 128.9 (C-9a), 129.2 (C-4), 130.4 (C-16c), 130.6 (C-4a), 132.1 (C-5), 136.2 (C-16a), 155.7 (C-6a), 156.3 (15a), 158.2 (C-14a), 165.1 (C-13), 175.8 (C-10) ppm. HRMS (ESI^+^): *m*/*z* [M^+^H]^+^ calcd for C_25_H_17_O_4_ 381.1121; found 381.1105.

### Biology

#### Cholinesterase Inhibition Assays

The inhibitory activity of the synthesized compounds toward *ee*AChE and eqBChE was evaluated spectrophotometrically through a 96-well microplate modified Ellman’s method.^39^ The solutions of both enzymes were prepared as 0.025 U/mL in phosphate buffer at pH 7, from stock solutions of 5.05 U/mL and 7.50 U/mL, respectively. The stock solutions for the target compounds (0.1 M) were prepared in dimethyl sulfoxide (DMSO). The other assay solutions consisted of 0.0005 M 5,5′-dithiobis­(2-nitrobenzoic acid) (DTNB), 0.0025 M acetylthiocholine iodide (ATChI) or 0.0025 M butyrylthiocholine iodide (BTChI), for the inhibition of *ee*AChE or *eq*BChE, respectively, and were prepared in 0.1 M phosphate buffer, at pH 7. The reaction mixture was prepared with 100 μL of the enzyme (*ee*AChE or *eq*BChE) and 50 μL of the test compound (or DMSO/water, not exceeding 0.2% of DMSO in the wells; *i.e* blank samples). After 5 min of the preincubation period at 37 °C, 50 μL of ATChI or BTChI and 50 μL of DTNB were added to each well, thus initiating the enzymatic reaction. The absorbance values were measured at 415 nm, for a total of 7.5 min, at every 2.5 min, using a microplate reader (Synergy multimode reader; BioTek). All compounds were tested at a screening concentration of 20 μM. The IC_50_ values were determined for the compounds with an inhibitory activity higher than 50%, at seven different concentrations of 12.5, 10, 7.5, 5, 2.5, 1, and 0.5 μM, using GraphPad Prism 8.0.2 (GraphPad Software Inc.) Donepezil was used as the reference compound. All experiments were performed in triplicate.

#### Kinetic Analysis

To assess the inhibition mechanism of the selected compounds toward both cholinesterases, a Lineweaver–Burk plot of 1/V versus 1/[S] was performed using five different concentrations of ATChI or BTChI substrate (50–800 μM), in the presence or absence of the compounds (PBS at pH 7 was used as the negative control). The enzymatic reactions and measurements were performed using the same assay conditions, as described above for the inhibition of both enzymes. All experiments were performed in triplicate. Linear regression analysis was performed with GraphPad Prism 8.0.2 (GraphPad Software Inc.).

#### Molecular Modeling, Molecular Docking and Molecular Dynamics Simulations

The crystal structures of the human acetylcholinesterase (*h*AChE) and butyrylcholinesterase (*h*BChE) enzymes complexed with the inhibitors huprine X and huprine 19 (PDBID 4BDT and 6EQQ, respectively) were retrieved from the Protein DataBank (http://www.rcsb.org).^54,55^ Computational studies were performed with both *h*AChE and *h*BChE because they have >85% sequence identity (100% conservation of the active site), relatively to *ee*AChE and *eq*BChE used in the inhibition assays. These structures and procedures were also used in previous docking studies.^25,56,57,58^ All crystallographic water and ligand molecules were removed, and polar hydrogen atoms were added at the physiological pH. The PROPKA program was used to check the p*K*_*a*_ values of all ionizable residues.^33^ In particular, the histidine from the catalytic triad (His447 and His438 of *h*AChE and *h*BChE) was protonated at the delta nitrogen. The GaussView software was used to build the 3D structures of the compounds.

The AutoDock VINA software was used for molecular docking calculations.^59,60^ The docking box was centered on the inhibitors of both cocrystal structures, and comprised a radius of 20 Å. For each calculation, 20 docking rounds were requested, including the full flexibility of the ligand and some active site residues (AChE: Tyr124, Ser203, Phe295, Tyr337, Phe338 and Tyr341; BChE: Ser198, Trp231, Phe329 and Tyr332). The visual molecular dynamics (VMD 1.9.2) program was used for visualization of the binding modes, analysis, and image rendering.^60^

Molecular docking was also performed to analyze the binding interactions and affinities of the 11r ligand with Aβ_42_. The 3D structure of Aβ_42_ was obtained from the Protein Data Bank (PDB ID: 5OQV)^61^ and prepared using AutoDock Tools (ADT) **version 1.5.6** by removing water molecules, adding polar hydrogens, and assigning Kollman charges. A blind docking approach was employed, where the grid box was set to encompass the entire protein structure, ensuring a comprehensive search for potential binding sites. Docking was performed using default parameters of AutoDock 4.2, and the best docking pose, selected based on binding affinity and interaction analysis, was used for subsequent molecular dynamics simulations. The best docking pose for the protein–ligand complex was used to prepare a system for molecular dynamics simulations using CHARMM-GUI. The CHARMM General Force Field (CGenFF) was used to derive the ligand parameters. The complex was solvated in a TIP3P water box, neutralized, and ionized to a physiological concentration of 0.15 M NaCl. The simulation box had a volume of 85 × 85 × 85 Å^3^. Periodic boundary conditions and Particle Mesh Ewald (PME) electrostatics were applied. All calculations were performed with the CHARMM36m force field using GROMACS 2021.4. Energy minimization was performed using the steepest descent algorithm to remove steric clashes and relax the system. Position restraints of 400 kJ/mol/nm^2^ were applied to the protein backbone. The systems were then equilibrated in two phases. First, a 250 ps NVT equilibration was conducted at 303.15 K using the v-rescale thermostat, and next a 250 ps of NPT equilibration at 1.0 bar using the Parrinello–Rahman barostat. Position restraints of 400 kJ/mol/nm^2^ on the backbone and 40.0 kJ/mol/nm^2^ on side chain atoms were maintained during the equilibration phase. Production MD was performed for 100 ns with a 2 fs time step. The systems were maintained at 303.15 K and 1.0 bar using the v-rescale thermostat and Parrinello–Rahman barostat, respectively. The Verlet cutoff scheme was used with a cutoff distance of 1.2 nm for both van der Waals and electrostatic interactions (PME). Bond lengths involving hydrogen atoms were constrained using the LINCS algorithm. Three replicates were performed. The trajectories were analyzed using VMD (version 1.9.5).

#### Inhibition Assay in *E. coli* Cells Overexpressing Amyloid Proteins

##### Cloning and Expression of Aβ_42_ Peptide

42 Amino acid Aβ_42_ was cloned and overexpressed in *E. coli* BL21 (DE3) competent cells using the pET vector harboring the DNA sequence of Aβ_42_. Due to the addition of the initiation codon ATG preceding genes, the resulting overexpressed peptide contained an additional methionine residue at its *N*-terminus. For the preparation of overnight cultures, a colony of BL21 (DE3) cells carrying the plasmid for expression was inoculated in 10 mL of M9 minimal medium supplemented with 50 μg mL^–1^ kanamycin at 37 °C. Subsequently, a 1:500 dilution of the overnight culture was added to fresh M9 minimal medium containing 50 μg mL^–1^ kanamycin and 25 μM ThS. The bacterial culture was then incubated at 37 °C with agitation at 250 rpm. Upon reaching an OD600 of 0.6, 980 μL of the culture was transferred into 1.5 mL Eppendorf tubes, each containing 10 μL of the compound to be tested dissolved in DMSO and 10 μL of 100 mM isopropyl 1-thio-β-D-galactopyranoside (IPTG) or, alternatively, 10 μL of water for the noninduced controls. The final compound concentration was adjusted to 10 μM. The samples were allowed to grow overnight at 37 °C with agitation at 1400 rpm using a Thermomixer (Eppendorf). Negative controls, representing maximum amyloid presence, utilized free DMSO.

##### Cloning and Expression of Full-Length Tau Protein

For the cloning and overexpression of full-length tau protein, *E. coli* BL21 (DE3) competent cells were transformed first with the pTARA vector containing the RNA polymerase gene of phage T7 (T7RP), controlled by the PBAD promoter. Subsequently, *E. coli* BL21­(DE3) competent cells containing pTARA were transformed with the pRKT42 vector encoding four repeats of tau protein in two inserts. Similar to the procedure for Aβ_42_, overnight cultures were prepared by inoculating colonies of BL21 (DE3) cells bearing the plasmids in M9 medium supplemented with 0.5% glucose, 50 μg mL^–1^ ampicillin, and 12.5 μg mL^–1^ chloramphenicol at 37 °C. Upon induction, a 1:500 dilution of the overnight culture was added to fresh M9 minimal medium containing 0.5% glucose, 50 μg mL^–1^ ampicillin, 12.5 μg mL^–1^ chloramphenicol, and 25 μM ThS. The subsequent steps mirrored those for the Aβ assay, including the addition of the compound to be tested dissolved in DMSO and 10 μL of 25% arabinose or, alternatively, 10 μL of water for the noninduced controls, with a final compound concentration of 10 μM. Negative controls for tau overexpression utilized free DMSO.

##### ThS Steady-State Fluorescence Analysis

ThS (T1892) and all other chemical reagents were purchased from Sigma (St. Louis, MO). Stock solutions of ThS (250 μM) were prepared using doubly distilled water purified via a Milli-Q system (Millipore). For the fluorescence assay, ThS spectra were recorded utilizing an AmincoBowman series 2 luminescence spectrophotometer (Aminco-Bowman AB2, SLM Aminco, Rochester, NY) within the range of 460 to 600 nm at a temperature of 25 °C. The excitation wavelength employed was 445 nm, with slit widths set at 4 nm. The emission at 485 nm, corresponding to the ThS peak observed in the presence of amyloids, was recorded. To standardize ThS fluorescence relative to bacterial concentration in both *in vitro* and cell-based assays, the OD600 was determined using a Shimadzu UV-2401 PC UV–vis spectrophotometer. Fluorescence normalization was executed by setting the ThS fluorescence of bacterial cells expressing the peptide or protein in the absence of the drug as 100%, while that of bacterial cells not expressing the peptide or protein was established as 0%.

#### BBB Permeability Prediction

The prediction of the brain penetration was evaluated using a PAMPA-BBB.^62^ Ten commercial drugs [(3–5 mg of caffeine, enoxacine, hydrocortisone, desipramine, ofloxacine, piroxicam, and testosterone), (12 mg of promazine) and 25 mg of verapamil and atenolol], purchased from Sigma-Aldrich were dissolved in EtOH (1000 μL). Next, 100 μL of these stock solutions were added to a solution of 1400 μL of EtOH and 3500 μL of PBS at pH 7.4, to reach 30% of EtOH concentration in the experiment. These solutions were filtered, using Filter PDVF membrane units (diameter 30 mm, pore size 0.45 μm, from Symta). After this, the acceptor 96-well microplate (Multiscreen, catalogue no. MAMCS9610, from Millipore) was filled with 180 μL of PBS/EtOH (70/30). The donor 96-well plate (Multiscreen IP sterile plate PDVF membrane, pore size is 0.45 μM, catalogue no. MAIPS4510, from Millipore) was coated with 4 μL of porcine brain lipid [(PBL) catalogue no. 141101, from Avanti Polar Lipids] in dodecane (20 mg/mL). After 5 min, 180 μL of each compound solution was added. 1–2 mg of each selected compound was dissolved in 1500 μL of EtOH and 3500 μL of PBS at pH 7.4, filtered, and then added to the donor 96-well plate to determine their ability to cross the brain barrier. After this, the donor plate was carefully put on the acceptor plate to form a “sandwich” and incubated for 2.5 h at 25 °C. During this period, the compounds diffused from the donor plate through the brain lipid membrane into the acceptor plate. Afterward, the donor plate was removed. A 96-well plate UV reader (Thermoscientific, Multiskan spectrum) was used to determine the concentration of compounds and commercial drugs in the acceptor and donor wells. Every sample was analyzed at three to five wavelengths, in three wells, and in two independent runs. Results were given as the mean ± SD [standard deviation], and the average of the two runs is reported. Ten quality control compounds (previously mentioned) of known BBB permeability were included in each experiment to validate the analysis set.

#### Calcein-AM-Assay

For all compounds tested, stock solutions were prepared in DMSO. Dilutions were then made with Krebs-Ringer buffer (KRB). The final concentration of DMSO on hMEC/D3 cells (a BBB model) did not exceed 1%. At this concentration, DMSO did not affect the assay. To perform calcein-AM-MDR-assay (calcein-AM multidrugs resistance) studies, cultured cells were washed 3 times with 37 °C KRB and subsequently incubated with increasing concentrations of the test compounds for 15 min at 37 °C. Calcein-AM (MoBiTec, Göttingen, FRG) was added to a final concentration of 1 μM and incubated for 30 min at 37 °C. Afterward, the cells were immediately washed 3 times with ice-cold KRB and lysed with 1% Triton-X-100. Fluorescence was measured using a Tecan Infinite 200 Pro plate reader with λ excitation = 485 nm and λ emission = 535 nm. Each test compound was measured with n = 3, control and background were measured with n = 3. All fluorescence values were corrected by subtracting the background fluorescence. The increase in cellular fluorescence caused by a test compound was referred to the fluorescence of the control. Thereby, the strong P-gp inhibitor PSC-833 (1 μM) was set as 100%.

#### Cytotoxicity Assessment

The MTT (Sigma-Aldrich, St Louis, MO, USA) was used to assess the viability of the cells in the presence of the selected compounds. The compounds were solubilized in 100% of DMSO to obtain a stock concentration of 10 mM. SH-SY5Y cells were seeded into a 96-well plate at a density of 2 × 10^4^ cells mL^–1^ and cultured with 100 μL of growth medium (DMEM supplemented with 10% FBS), for 24 h at 37 °C, in a 5% CO_2_ incubator. After that period, cells were treated with various concentrations of the selected compounds (100, 50, 25, and 10 μM) or the correspondent solvent percentage (1, 0.5, 0.25 and 0.1%) and incubated for another 24 h, in a 100 μL final volume mixture. For untreated cells and blank control, 100 μL of the growth medium without treatment was added. Compound-containing solutions were sterilized using a 0.2 mm filter. Then, 10 μL of a MTT solution (5 mg mL^–1^ in PBS) was added to each well and incubated for 2 h at growing conditions. Next, the culture media was removed, the formazan crystals formed dissolved with 100 μL of DMSO and incubated for 10 min at room temperature. Finally, the absorbance intensity was measured at 575 nm using a microplate reader (Microplate Reader Infinite 200, Tecan Life Sciences). Each condition was measured in triplicate and experiments repeated three times independently. Untreated cells were used as control and the blank value was subtracted to all conditions.

#### Interaction with Aβ_42_ Peptide

##### Aβ_42_ Peptide Preparation

Synthetic Aβ_42_ (GenScript) was dissolved in hexafluoro-2-propanol (HFIP) (Merck) and left at room temperature (RT) for 48 h. Subsequently, the HFIP was removed using a stream of nitrogen, and the resulting powder was dissolved in dimethyl sulfoxide (DMSO) (Sigma-Aldrich) at a concentration of 2000 μM. For oligomer and fibril formation, Aβ_42_ was diluted in F-12 medium (Alfagene) to a concentration of 10 μM and incubated for 48 h at 37 °C.

##### TEM

The βnalysis of the morphology and fibril formation of samples coincubated with Aβ_42_ (10 μM) (GenScript) and the selected compounds (50 μM) at different time-points (6, 12, 24, and 48 h) at 37 °C, was assessed through TEM. Five μL sample aliquots were adsorbed into carbon-coated collodion film supported on 300-mesh copper grids (Electron Microscopy Sciences, PA, USA) and negatively stained twice with 1% (m/v) uranyl acetate (Electron Microscopy Sciences, PA, USA). The grids were then visualized using a JEOL (Tokyo, Japan) JEM- 400 transmission electron microscope, operated at 80 kV, equipped with an Orious (CA, USA) Sc1000 digital camera, and exhaustively observed.

##### Confocal Microscopy

A stock solution of Aβ_42_ (GenScript) was prepared in DMSO to a concentration of 2000 μM. SH-SY5Y cells stably expressing wild-type APP695 (SHwt) were used. The cells were cultured in Dulbecco’s Modified Eagle Medium (DMEM, Gibco), supplemented with 10% of Fetal Bovine Serum (FBS, Sigma-Aldrich), and 2% of Pen-Strep-Glut (Gibco). The medium was replaced every 2–3 days, and the cells were kept at 37 °C in a humidified environment (95%) with 5% of CO_2_. 2.0 × 10^4^ SH-WT cells were plated in 24-well plates and incubated for 24 h, in 12 ømm glass coverslips. Then, 10 μM of exogenous monomeric Aβ_42_ and 25 μM of the test compound were coincubated in DMEM (without FBS) for 16 h. Cells incubated with Aβ_42_ and 1% DMSO were used as the positive control. After this period, the medium was changed to fresh DMEM medium without FBS, and the cells were incubated for another 24 h. After the incubation period, cells were washed with PBS, fixed in 4% formaldehyde for 30 min, permeabilized with 0.2% Triton X-100 in PBS for 10 min and blocked with 1% BSA for 10 min. The Aβ_42_ aggregates were stained with ThT at 40 μM for 30 min, and the cells’ cytoskeleton was stained with Phalloidin (Alexa Fluor 568, 1:300) for 1 h. Cells were washed three times between each step. Coverslips with cells were mounted with CitiFluor MWL4–88 (Electron Microscopy Sciences). Confocal images were acquired with a Zeiss LSM 880 confocal microscope (Carl Zeiss, Jena, Germany), and a Plan-Apochromat 20*x*/0.8 M27 objective, using 405 and 561 nm laser lines for ThT and Phalloidin excitation, respectively, with a minimum of 20 cells analyzed per condition.

## Supplementary Material



## Notes

Synthesis of intermediates (Schemes S1–S4) and characterization data (HPLC and 1H, 13C, and 19F NMR spectra) of all novel compounds in this publication, procedures and results from molecular modeling, molecular docking and dynamics simulation studies (Figure S1–S2 and Tables S1–S2), BBB permeability assay (Figure S3) (PDF)

## Abbreviations

Aβ: β-amyloid
AChE: Acetylcholinesterase
AD: Alzheimer’s disease
BBB: Blood–brain barrier
BChE: Butyrylcholinesterase
CAS: Catalytic site
CNS: Central nervous system
DA: Diels–Alder
DMTs: Disease-modifying therapies
*E. coli*: *Escherichia coli*
HB: Hydrogen bond
MTDL: Multitarget directed ligand
MW: Microwave
NMR: Nuclear magnetic resonance
PAMPA: Parallel artificial membrane permeability assay
PAS: Peripheral anionic site
SAR: Structure–activity relationship
SH-SY5Y: Human neuroblastoma cell line
TEM: Transmission electron microscopy
ThS: Thioflavin S
ThT: Thioflavin T
TLC: Thin layer chromatography
