# Supplementary material for: Design, Synthesis and Biological Evaluation of Chromeno[3,4‑b]xanthones as Multifunctional Agents for Alzheimer’s Disease
Source: ACS Chem Neurosci. 2025 Aug 6;16(16):3184–203. doi: 10.1021/acschemneuro.5c00425 (PMC12818745; doi:10.1021/acschemneuro.5c00425)
Supplement: Supplementary file 1 [file cn5c00425_si_001.pdf]

Supporting Information (SI)

Design, Synthesis and Biological Evaluation of Chromeno[3,4-*b*]xanthones as Multifunctional Agents for Alzheimer's Disease

Daniela Malafaia[a], Natércia F. Brás[b], Anna Sampietro[c], Inês Quintelas[b], Pedro Ferreira[b], Lúcia Melo[a], Joana Saavedra[d,e], Loreto Martinez-Gonzalez[f], Marisa Pereira[g], Jessica Sarabando[g], Leo König[h], Isabel Cardoso[d,e] Daniela Ribeiro[g], Ana R. Soares[g], Raimon Sabaté[i], Gert Fricker[h], Ana Martinez[f], Pedro A. Fernandes[b], Artur M. S. Silva[a], Hélio M. T. Albuquerque[a,*]

[a]LAQV-REQUIMTE, Department of Chemistry, University of Aveiro, 3810-193 Aveiro, Portugal.

[b]LAQV-REQUIMTE, Department of Chemistry and Biochemistry, University of Porto, 4169-007 Porto, Portugal.

[c]Laboratory of Medicinal Chemistry (CSIC Associated Unit), Faculty of Pharmacy and Food Sciences, and Institute of Biomedicine (IBUB), University of Barcelona, Barcelona, Spain.

[d]Molecular Neurobiology Group, Institute for Health Research and Innovation (i3S), Institute for Molecular and Cellular Biology (IBMC), Porto, Portugal.

[e]Department of Molecular Biology, Abel Salazar Institute of Biomedical Sciences (ICBAS), University of Porto, Porto, Portugal.

[f]Centro de Investigaciones Biologicas, CSIC, Ramiro de Maeztu 9, 28040 Madrid, Spain. Centro de Investigación Biomédica en Red de Enfermedades Neurodegenerativas (CIBERNED), Instituto de Salud Carlos III, Madrid, Spain.

[g]Institute of Biomedicine (iBiMED), Department of Medical Sciences, University of Aveiro, 3810-193 Aveiro, Portugal.

[h]Ruprecht-Karls Universität Institut für Pharmazie und Molekulare Biotechnologie Im Neuenheimer Feld 329, D-69120 Heidelberg.

[i]Department of Pharmacy and Pharmaceutical Technology, Department of Physical Chemistry, School of Pharmacy, iUniversity of Barcelona, Barcelona, Spain; Institute of Nanoscience and Nanotechnology (IN2UB), University of Barcelona, Barcelona, Spain.

*Email: helio.albuquerque@ua.pt

Table of contents

MOLECULAR MODELING AND MOLECULAR DOCKING 3

MOLECULAR DYNAMICS SIMULATION 7

BBB PERMEABILITY 8

CHEMISTRY 9

COMPOUND PURITY AND HPLC TRACES 13

^{1}H, ^{13}C AND ^{19}F NMR SPECTRA 16

REFERENCES 52

Molecular modeling and molecular docking

The docking protocol was validated by the re-docking of huprine X and huprine 19 inside AChE and BChE, respectively. Considering the similar position (Figure S1) and the small root-mean-square-deviation (RMSD) values between both X-ray and top-ranked docking poses, the use of parameters fit for describing the binding mode of the compounds under evaluation was deemed. The binding pocket of AChE is a long and narrow channel extending from the surface to the CAS, composed by the triad Ser203, His447 and Glu334. The channel entrance, also called peripheral anionic site (PAS), is relatively open and consists of the aromatic residues Tyr72, Tyr124 and Trp286. The binding pocket of BChE is quite similar, except larger due to the presence of aromatic residues (Trp231, Phe329 and Phe398) around the catalytic triad (Ser198, His438, and Glu325). The PAS site of BChE is composed by the Asp70, Ser72 and Tyr332 residues.

Tables S1 and S2 summarize the main interactions with AChE and BChE, respectively, as well as the binding modes of the compounds with IC_{50} < 10 μM against at least one of the enzymes.

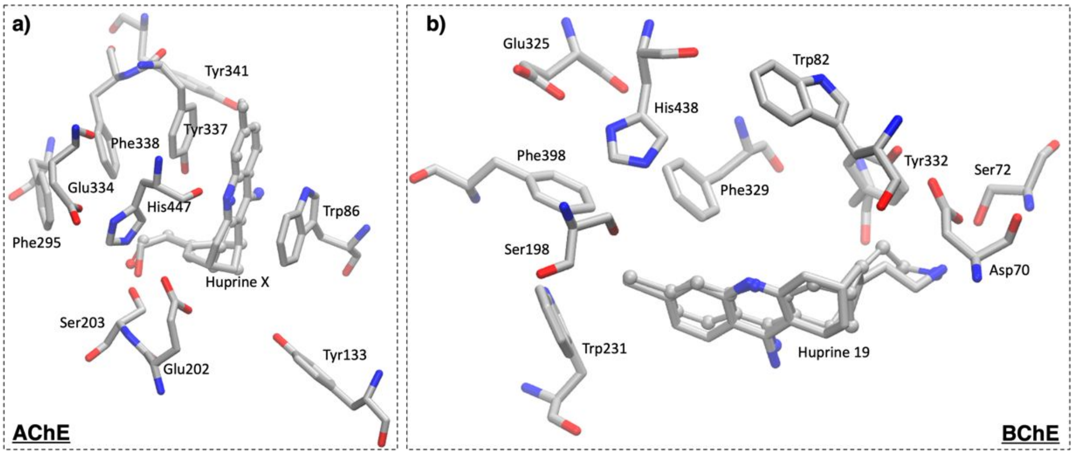

Figure S1. Superposition of the crystallographic ligands (huprine X and huprine 19, depicted in sticks) with the respective top-ranked docking poses (depicted in balls-and-sticks) with the active sites of AChE and BChE. Enzyme residues and ligands are colored by atom type.

Table S1. Entrance mode of the most promising compounds in the AChE active site as well as the main interactions and interacting residues. HB = Hydrogen bond.

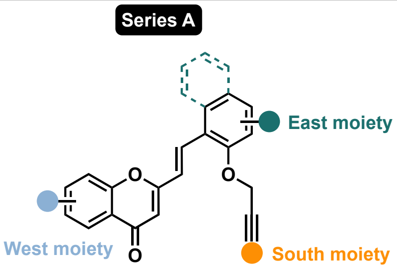

(*E*)-2-Styrylchromones
7, **8** or **10**

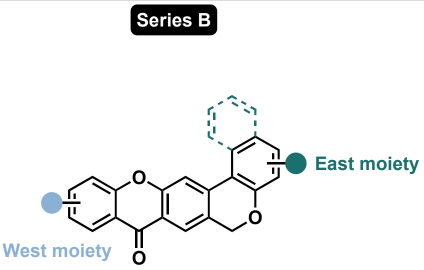

Chromeno[3,4-*b*]xanthones
11 or **12**

Compound	Entrance mode in the active site	Main interactions and interacting residues
**Series A**		
7a	West moiety	HB: Tyr124, Ser203, Tyr337<br>Hydrophobic/CH-π: Trp286, Phe338<br>π-stacking: Trp86, Tyr341
7d	West moiety	HB: Tyr124, Ser125, Ser203<br>Hydrophobic: Leu130<br>Hydrophobic/CH-π: Phe338, Tyr341<br>π-stacking: Trp86, Phe338, Tyr341
7o	West moiety	HB: Tyr124, Ser203<br>π-stacking: Trp86, Tyr124, Trp286, Tyr337, Phe338, Tyr341
10	West moiety	HB: Tyr124, Ser203<br>Hydrophobic/CH-p: Trp286, Phe297, Tyr341<br>π-stacking: Trp86, Tyr337, Phe338, Tyr341
	East moiety	HB: Phe295<br>π-stacking: Tyr72, Trp232, Tyr337, Tyr341
**Series B**		
11a	West moiety	HB: Ser125, Ser203<br>π-stacking: Trp86, Tyr337, Phe338, Tyr341
	East moiety	HB: Ser203, Tyr124, Phe295<br>π-stacking: Trp86, Trp286, Tyr337, Phe338, Tyr341
11b	West moiety	HB: Ser125, Ser203<br>Hydrophobic: Leu130

		π-stacking: Trp86, Tyr337, Phe338
	East moiety	HB: Tyr124, Ser203, Arg296<br>π-stacking: Trp86, Tyr337, Phe338, Tyr341
**11f**	West moiety	HB: Tyr124, Ser203, Phe295, Tyr337<br>Hydrophobic/CH-p: Trp286<br>π-stacking: Trp86, Trp286, Tyr337, Phe338, Tyr341
	East moiety	π-stacking: Trp86, Tyr337, Phe338, Tyr341
**11h**	West moiety	HB: Tyr124, Ser203, Phe295, Tyr337<br>π-stacking: Trp86, Tyr337, Phe338, Tyr341<br>Halogen-π: Trp286<br>Halogen bond (σ hole): Tyr72
**11k**	West moiety	HB: Tyr124, Ser203, Phe295, Tyr337<br>π-stacking: Trp86, Tyr337, Phe338, Tyr341<br>Halogen-π: Trp286<br>Halogen bond (σ hole): Tyr72, Ser293, Tyr341
**11l**	West moiety	Hydrophobic: Leu130<br>π-stacking: Trp86, Tyr337, Phe338, Tyr341
	East moiety	HB: Tyr124, Ser203, Arg296<br>π-stacking: Trp86, Tyr337, Phe338, Tyr341
**11q**	West moiety	HB: Ser125, Ser203<br>Hydrophobic: Pro88<br>π-stacking: Trp86, Phe338, Tyr341<br>Halogen-π: Tyr341
	East moiety	HB: Tyr124<br>π-stacking: Trp86, Tyr337, Tyr341<br>Halogen bond (σ hole): Gly120, Ser125
**11r**	West moiety	HB: Tyr124, Ser203<br>Hydrophobic: Leu130<br>π-stacking: Trp86, Tyr337, Phe338, Tyr341<br>Halogen-π: Trp286
	East moiety	HB: Tyr124<br>π-stacking: Trp86, Tyr337<br>Halogen bond (σ hole): Gly116, Ser121
**11s**	West moiety	HB: Ser203, Phe295

		Hydrophobic: Leu130<br>π-stacking: Trp86, Phe297, Tyr337, Phe338, Tyr341<br>Halogen-π: Trp286<br>halogen bond (σ hole): Tyr72, Ser293, Phe338, Tyr341

Table S2. Entrance mode of the most promising compounds in the BChE active site as well as the main interactions and interacting residues.

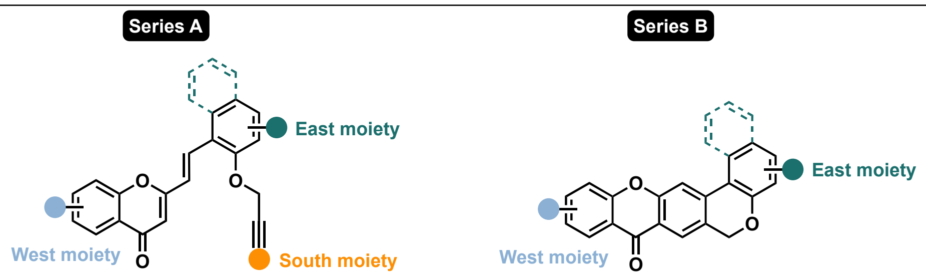

(*E*)-2-Styrylchromones **7**, **8** or **10**

Chromeno[3,4-*b*]xanthones **11** or **12**

Compound	Entrance mode in the active site	Main interactions and interacting residues
	**Series A**	
**10**	West moiety	HB: Ser198<br>Hydrophobic: Pro285, Asp70<br>Hydrophobic/CH-π: Tyr332<br>π-stacking: Trp231, Phe329, Phe398
	East moiety	HB: Asp70<br>π-stacking: Trp82
	**Series B**	
**11d**	East moiety	HB: Ser198, Thr120<br>π-stacking: Trp231, Phe329, Phe398
**11e**	West moiety	HB: Ser198, Thr120<br>Hydrophobic/CH-π: Trp231<br>π-stacking: Phe329
	East moiety	HB: Ser198, Gly116<br>π-stacking: Trp82, Phe329, Tyr332
**11f**	West moiety	HB: Ser198, Thr120<br>π-stacking: Trp86, Trp231, Phe329, Phe398
	East moiety	HB: Ser198

		π-stacking: Trp231, Phe329
**11h**	West moiety	HB: Ser198, Thr120<br>π-stacking: Trp231, Phe329, Phe398
	East moiety	HB: Ser198<br>π-stacking: Trp82, Phe329, Tyr332, Trp430<br>Halogen-π: Trp82<br>Halogen bond (σ hole): Trp82, Gly115, Tyr128
**11k**	West moiety	HB: Ser198<br>π-stacking: Trp82, Phe329<br>Halogen bond (σ hole): Ser72, S292
	East moiety	HB: Ser72<br>π-stacking: Phe329, Tyr332<br>Halogen bond (σ hole): Ser198, Trp82
**11l**	East moiety	HB: Ser198, Gly116<br>Hydrophobic: Ala325<br>π-stacking: Trp82, Phe329, Tyr332
**11m**	West moiety	HB: Ser198, Asn289<br>π-stacking: Trp231, Phe329
	East moiety	HB: Ser198, Gly116<br>π-stacking: Trp82, Phe329, Tyr332
**11r**	East moiety	HB: Ser72<br>Hydrophobic: Pro285<br>π-stacking: Trp82, Phe329, Tyr332<br>Halogen-π: Ser198
	West moiety	Hydrophobic: Met442<br>π-stacking: Trp82, Phe329
**11s**	East moiety	HB: Ser72<br>π-stacking: Phe329, Tyr332<br>Halogen-π: Trp82<br>Halogen bond (σ hole): Ser195

Molecular dynamics simulation

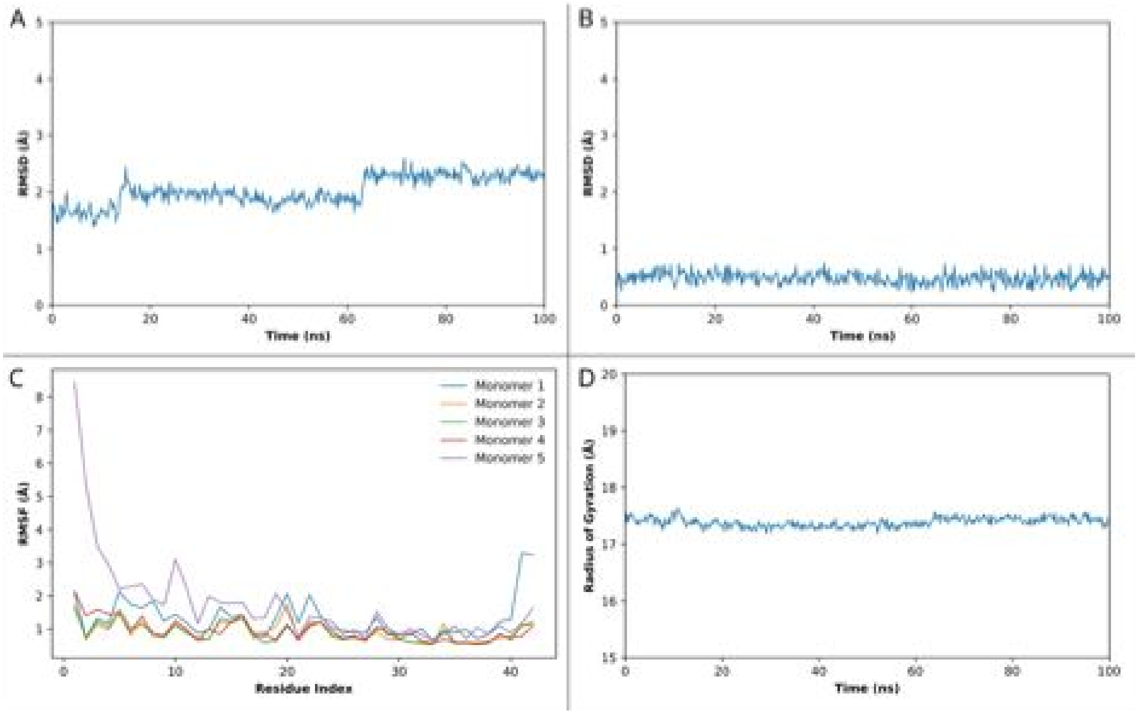

Figure S2. Time evolution of structural metrics during the 100 ns production run. (A) Backbone RMSD of the five subunits of the A$\beta_{(1–42)}$ fibril and (B) RMSD of compound **11r** atoms, both computed relative to the minimized starting structure. The fibril backbone RMSD remains below 2.5 Å, with a modest increase around 65 ns that correlates with enhanced flexibility of the N-terminal segment of monomer 5. Owing to the staggered arrangement of the monomers, N-terminal regions typically contact the subsequent monomer; in monomer 5 this region is exposed to the solvent, explaining the higher mobility. (C) Per residue RMSF profiles for each monomer highlight the increased fluctuations at the N-terminus of monomer 5, whereas the rest of the fibril shows RMSF values mostly <2 Å. (D) The radius of gyration of the fibril remains essentially constant throughout the simulation.

BBB permeability

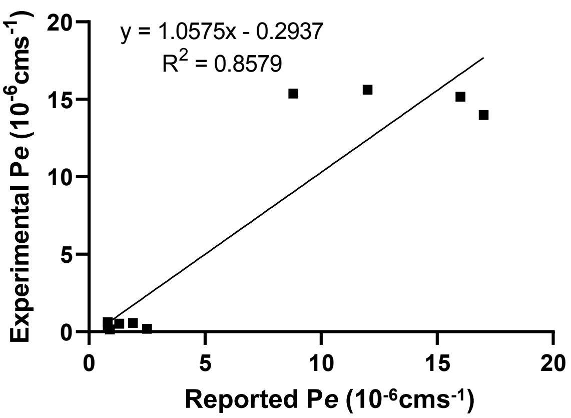

Figure S3. Linear correlation between experimental and reported permeability of commercial drugs using the PAMPA-BBB assay.

Chemistry

General procedure for the synthesis of compounds 4a-e. (i) To a solution of an appropriate 2'-hydroxyacetophetone (24 mmol) in acetone (100 mL) was added potassium carbonate (1.5 equiv for the methylation of each hydroxyl group) and dimethyl sulfate (1.1 equiv for the methylation of each hydroxy group). The resulting mixture was refluxed for 15 min. After that period, the mixture was rinsed with acetone (50 mL) by filtration, evaporated under reduced pressure and purified by silica-gel column chromatography using DCM as eluent. (ii) and (iii) To a solution of the appropriate 2'-hydroxyacetophenone (7.3 mmol) in EtOAc (60 mL) was added sodium (1.0 g, 43.8 mmol). The mixture was stirred at room temperature for 4 h. After that period, the mixture was poured into water (50 mL) and ice (50 g) and the pH adjusted to 4 with dilute HCl (10%). The aqueous phase was extracted with dichloromethane (3 x 100 mL), dried over anhydrous Na_2SO_4 and concentrated to obtain the β-diketone crude. The resulting β-diketone crude was further dissolved in DMSO (10 mL) and *p*-toluenesulfonic acid (*p*-TSA) (0.6 g, 3.65 mmol) was added. The mixture was stirred at 100 °C for 1 h. After that period, the mixture was poured into water (50 mL) and ice (50 g) and the aqueous phase was extracted with EtOAc (3 x 50 mL), dried over anhydrous Na_2SO_4 and concentrated to a crude oil. The residue was then purified by silica-gel column chromatography using DCM as eluent.

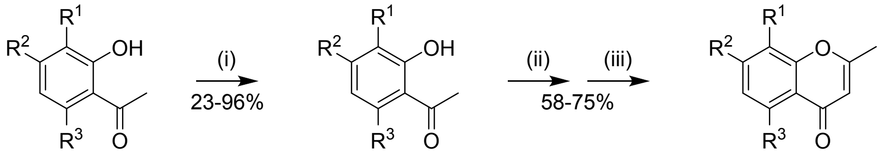

4a-e

4a: $R^1 = R^2 = R^3 = H$
4b: $R^1 = R^3 = H$; $R^2 = OCH_3$
4c: $R^1 = R^2 = H$; $R^3 = OCH_3$
4d: $R^1 = R^2 = OCH_3$; $R^3 = H$
4e: $R^1 = H$; $R^2 = R^3 = OCH_3$

Scheme S1. Synthesis of starting 2-methylchromones **4a-e**. Reagents and conditions: (i) K_2CO_3, Me_2SO_4, acetone, reflux, 15 min; (ii) Na, EtOAc, rt, 4 h; (iii) *p*-TSA, DMSO, 100 °C, 1 h.

2-Methyl-4*H*-chromen-4-one (4a): yield 849 mg (73%). Both spectroscopic and analytic data are in accordance with those previously reported.[1]

7-Methoxy-2-methyl-4*H*-chromen-4-one (4b): yield 380 mg (33%). Both spectroscopic and analytic data are in accordance with those previously reported.[1]

5-Methoxy-2-methyl-4*H*-chromen-4-one (4c): yield 787 mg (69%). Both spectroscopic and analytic data are in accordance with those previously reported.[1]

7,8-Dimethoxy-2-methyl-4*H*-chromen-4-one (4d): yield 1.30 g (quantitative). Both spectroscopic and analytic data are in accordance with those previously reported.[1]

5,7-Dimethoxy-2-methyl-4*H*-chromen-4-one (4e): yield 760 mg (69%). Both spectroscopic and analytic data are in accordance with those previously reported.[2]

General procedure for the synthesis of *O*-propargylsalicylaldehydes 5a-h and 6. To a solution of the appropriate salicylaldehyde (8.2 mmol) in acetonitrile (60 mL) was added K_2CO_3 (1.7 g, 12.3 mmol) and propargyl bromide (0.3 mL, 4.1 mmol) for aldehydes **5a-g** and **6** or chloroacetonitrile (0.5 mL, 4.1 mmol) for aldehyde **5h**. The resulting mixture was refluxed for 2 h. After that period, the mixture was rinsed with acetone (50 mL) by filtration and evaporated under reduced pressure.

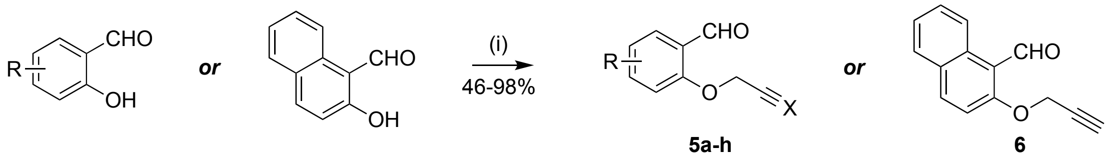

5a-h **6**

5a: R = H, X = H
5b: R = 5-OCH_3, X = H
5c: R = 5-Br, X = H
5d: R = 5-OCF_3, X = H
5e: R = 5-F, X = H
5f: R = 5-Cl, X = H
5g: R = 3-Br, 5-Cl, X = H
5h: R = H, X = N

Scheme S2. Synthesis of starting *O*-propargylsalicylaldehydes **5a-h** and **6**. Reagents and conditions: (i) Propargyl bromide or Chloroacetonitrile, K_2CO_3, MeCN, reflux, 2 h.

2-(Prop-2-yn-1-yloxy)benzaldehyde (5a): yield 1.00 g (76%). Both spectroscopic and analytic data are in accordance with those previously reported.[3]

5-Methoxy-2-(prop-2-yn-1-yloxy)benzaldehyde (5b): yield 874 mg (70%). Both spectroscopic and analytic data are in accordance with those previously reported.[4]

5-Bromo-2-(prop-2-yn-1-yloxy)benzaldehyde (5c): yield 951 mg (80%). Both spectroscopic and analytic data are in accordance with those previously reported.[5]

2-(Prop-2-yn-1-yloxy)-5-(trifluoromethoxy)benzaldehyde (5d): orange oil; yield 1.00 g (84%). ^{1}H NMR (300 MHz, $CDCl_3$): δ = 2.61 (t, 1H, H-3', *J* 2.4 Hz), 4.85 (d, 2H, H-1', *J* 2.4 Hz), 7.17 (d, 1H, H-3, *J* 9.1 Hz), 7.42 (dd, 1H, H-4, *J* 9.1, 2.2 Hz), 7.70 (d, 1H, H-6, *J* 2.2 Hz), 10.42 (s, 1H, C*H*O) ppm. ^{13}C NMR (75 MHz, $CDCl_3$): δ = 56.8 (C-1'), 77.0 (C-3'), 77.5 (C-2'), 114.8 (C-3), 120.8 (C-6), 122.1 (q, 5-O*C*F_3, *J* 257.6 Hz), 126.1 (C-1), 128.3 (C-4), 143.8 (C-5), 158.0 (C-2), 188.2 (*C*HO) ppm. ^{19}F NMR (282 MHz, $CDCl_3$): δ -55.09 (s) ppm.

5-Fluoro-2-(prop-2-yn-1-yloxy)benzaldehyde (5e): yield 1.10 g (87%). Both spectroscopic and analytic data are in accordance with those previously reported.[6]

5-Chloro-2-(prop-2-yn-1-yloxy)benzaldehyde (5f): yield 1.28 g (quantitative). Both spectroscopic and analytic data are in accordance with those previously reported.[6]

3-Bromo-5-chloro-2-(prop-2-yn-1-yloxy)benzaldehyde (5g): yield 1.09 g (95%). Both spectroscopic and analytic data are in accordance with those previously reported.[7]

2-(2-Formylphenoxy)acetonitrile (5h): yield 808 mg (61%). Both spectroscopic and analytic data are in accordance with those previously reported.[8]

2-(Prop-2-yn-1-yloxy)-1-naphthaldehyde (6): yield 867 mg (71%). Both spectroscopic and analytic data are in accordance with those previously reported.[6]

General procedure for the synthesis of aryl-*O*-propargylsalicylaldehydes 5i-k. To a solution of the appropriate 2-(prop-2-yn-1-yloxy)benzaldehyde **5a** (0.4 g, 2 mmol) in dry THF, (8 mL) iodobenzene (2.2 equiv), piperidine (4.0 equiv), $Pd(PPh_3)_2Cl_2$ (0.06 equiv) and CuI (0.12 equiv) were added. The reaction mixture was stirred at room temperature under N_2 atmosphere for 24 h. After that period, the solvent was evaporated under reduced pressure and the residue purified by preparative thin layer chromatography (TLC), using Hex/DCM (2:1) as eluent.

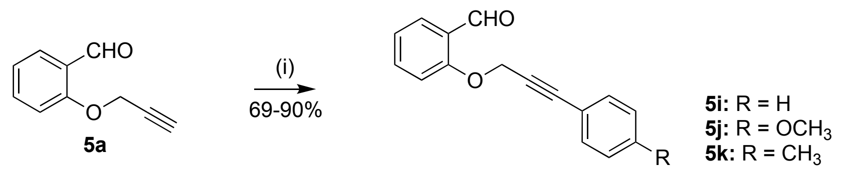

Scheme S3. Synthesis of the starting aryl-*O*-propargylsalicylaldehydes **5i-k**. Reagents and conditions: (i) Ar-I, Piperidine, $Pd(PPh_3)_2Cl_2$, CuI, THF, rt, 24 h.

2-[(3-Phenylprop-2-yn-1-yl)oxy]benzaldehyde (5i): yield 170 mg (58%). Both spectroscopic and analytic data are in accordance with those previously reported.[9]

1-(2-{[3-(4-Methoxyphenyl)prop-2-yn-1-yl]oxy}phenyl)ethan-1-one (5j): yield 240 mg (75%). Both spectroscopic and analytic data are in accordance with those previously reported.[9]

2-{[3-(4-Methylphenyl)prop-2-yn-1-yl]oxy}benzaldehyde (5k): yield 269 mg (83%). Both spectroscopic and analytic data are in accordance with those previously reported.[9]

General procedure for the synthesis of *N*-propargyl-2-aminobenzaldehyde 9. To a solution of (2-aminophenyl)methanol (40 mmol) in ACN (100 mL) was added K_2CO_3 (11.89 g, 100 mmol) and propargyl bromide (8.6 mL, 100 mmol). The resulting mixture was refluxed for 2 h. After that period, the mixture was poured into water (100 mL) and ice (100 g) and the pH adjusted to 4 with dilute HCl (10%). The precipitate was washed with water (50 mL) and recovered by filtration. To a solution of the resulting alcohol (4.00 g, 20 mmol) in EtOAc (30 mL) was added activated MnO_2

(8.70 g, 100 mmol) and the mixture refluxed for 4 h. The reaction mixture was allowed to cool to room temperature, filtered through a pad of celite and concentrated under vacuo. The crude residue was then purified by silica-gel column chromatography, using DCM as eluent.

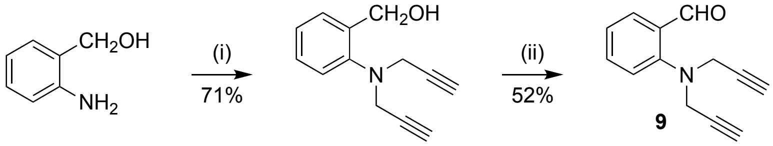

Scheme S4. Synthesis of starting *N*-propargyl-2-aminobenzaldehyde **9**. Reagents and conditions: (i) Propargyl bromide, K_2CO_3, MeCN, reflux, 2 h; (ii) Mn(IV)O_2, EtOAc, reflux, 4 h.

2-[Di(prop-2-yn-1-yl)amino]benzaldehyde (9): yield 2.10 g (52%). Both spectroscopic and analytic data are in accordance with those previously reported.[8]

Compound Purity and HPLC traces

Method: compounds purity was determined using a Waters Spherisorb S5 ODS2, LC Column 250 x 4.6 mm and Thermo Scientific UltiMate 3000 HPLC System. HPLC parameters were the following: ACN/H_2O/trifluoroacetic acid 45/55/0.05%; flow rate: 1 mL/min; elution type: isocratic; detection UV-Vis Abs at 250 nm. The samples were dissolved in ACN/H_2O/trifluoroacetic acid 45/55/0.05%.

Compound 10

Chromatogram

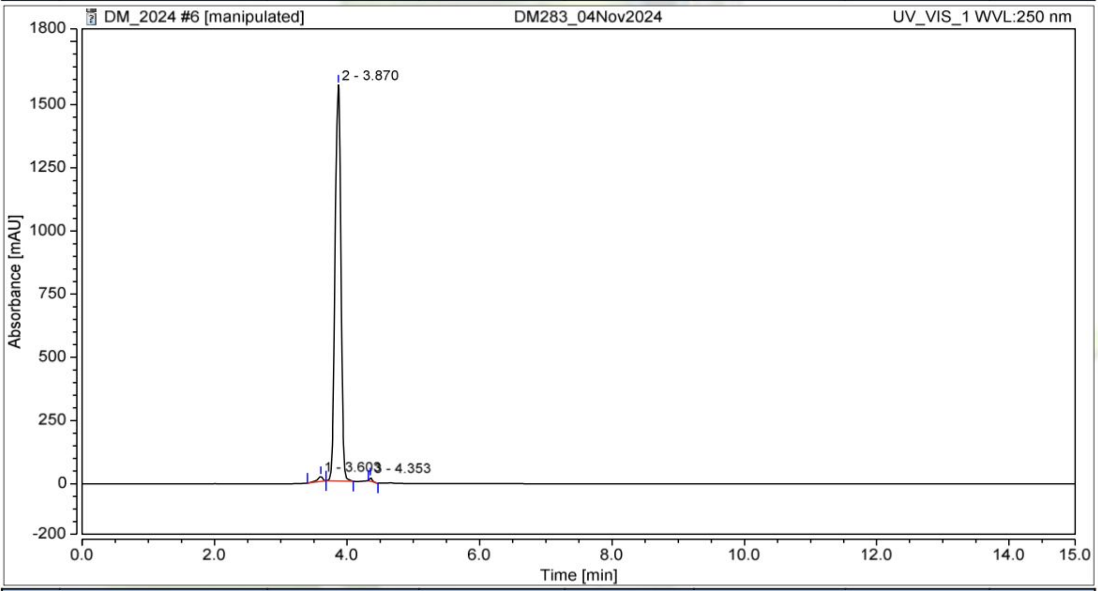

Integration Results

No.	Peak Name	Retention Time min	Area mAU*min	Height mAU	Relative Area %	Relative Height %	Amount n.a.
1		3.603	1.830	19.375	1.10	1.21	n.a.
2		3.870	163.667	1567.379	98.56	97.91	n.a.
3		4.353	0.557	14.043	0.34	0.88	n.a.
**Total:**			**166.054**	**1600.797**	**100.00**	**100.00**	

Compound 11q

Chromatogram

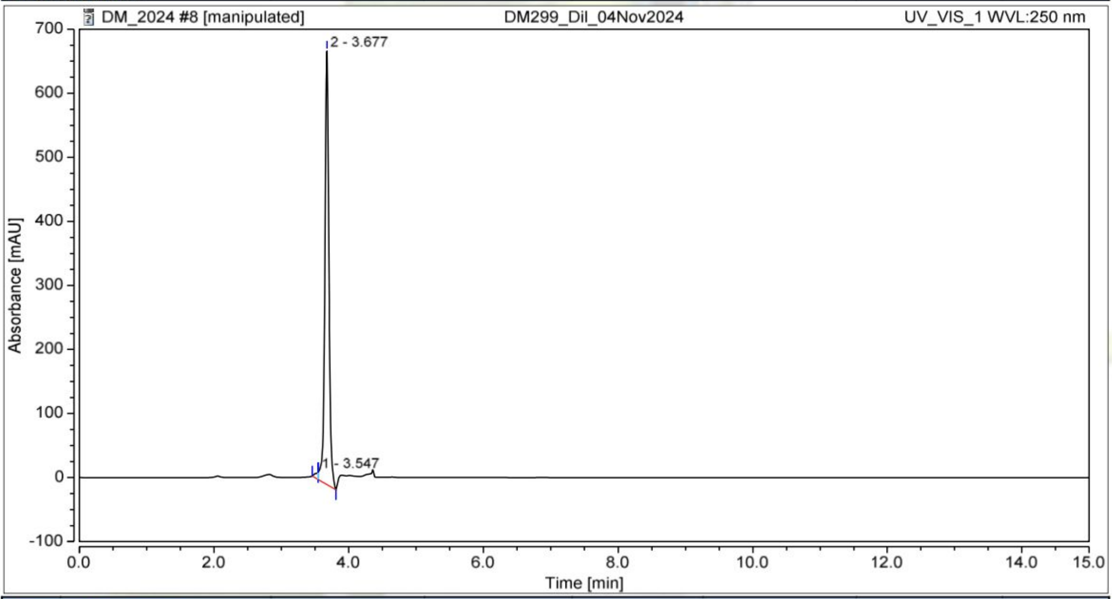

Integration Results

No.	Peak Name	Retention Time min	Area mAU*min	Height mAU	Relative Area %	Relative Height %	Amount n.a.
1		3.547	0.528	10.695	1.12	1.56	n.a.
2		3.677	46.449	676.486	98.88	98.44	n.a.
**Total:**			**46.977**	**687.181**	**100.00**	**100.00**	

Compound 11r

Chromatogram

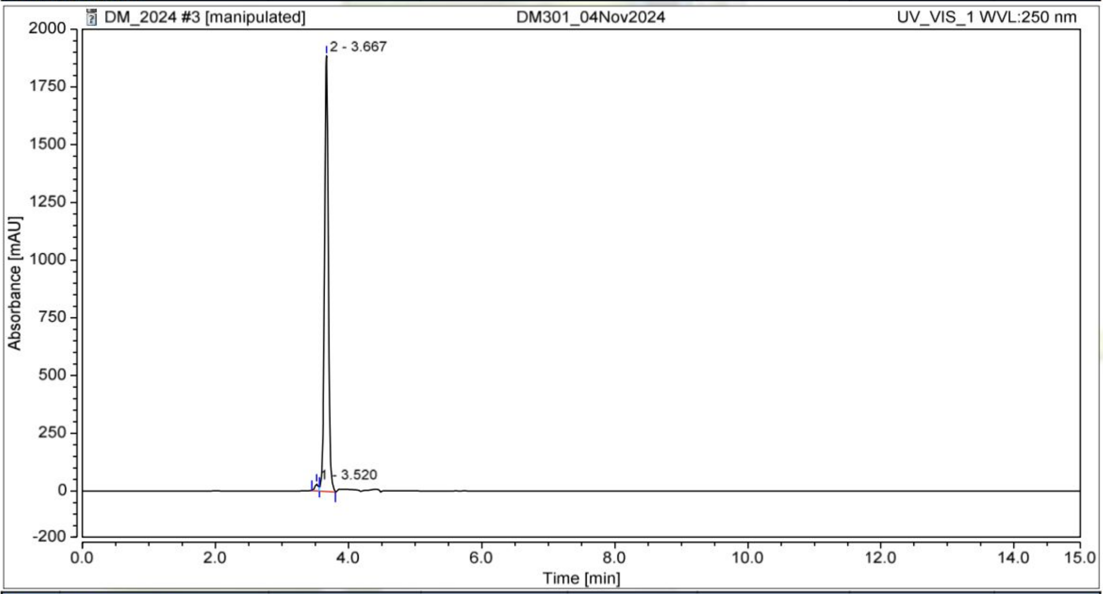

Integration Results

No.	Peak Name	Retention Time min	Area mAU*min	Height mAU	Relative Area %	Relative Height %	Amount n.a.
1		3.520	1.809	29.177	1.40	1.52	n.a.
2		3.667	127.570	1886.459	98.60	98.48	n.a.
**Total:**			**129.378**	**1915.635**	**100.00**	**100.00**	

Compound 11s

Chromatogram

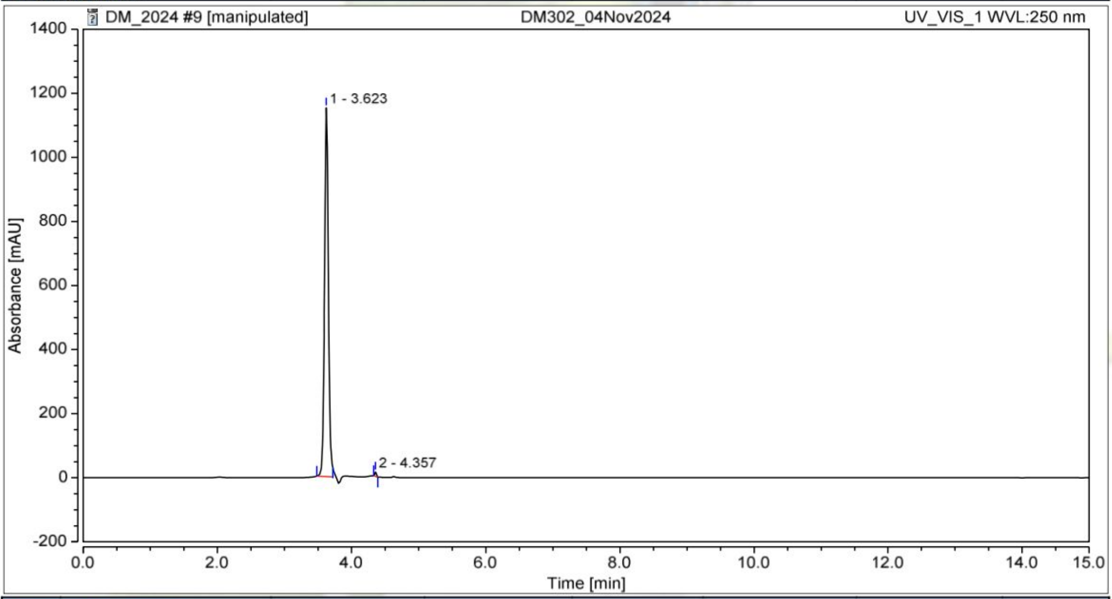

Integration Results

No.	Peak Name	Retention Time min	Area mAU*min	Height mAU	Relative Area %	Relative Height %	Amount n.a.
1		3.623	75.722	1150.817	99.54	98.86	n.a.
2		4.357	0.352	13.320	0.46	1.14	n.a.
**Total:**			**76.074**	**1164.137**	**100.00**	**100.00**	

^{1}H, ^{13}C and ^{19}F NMR spectra

Compound 5d

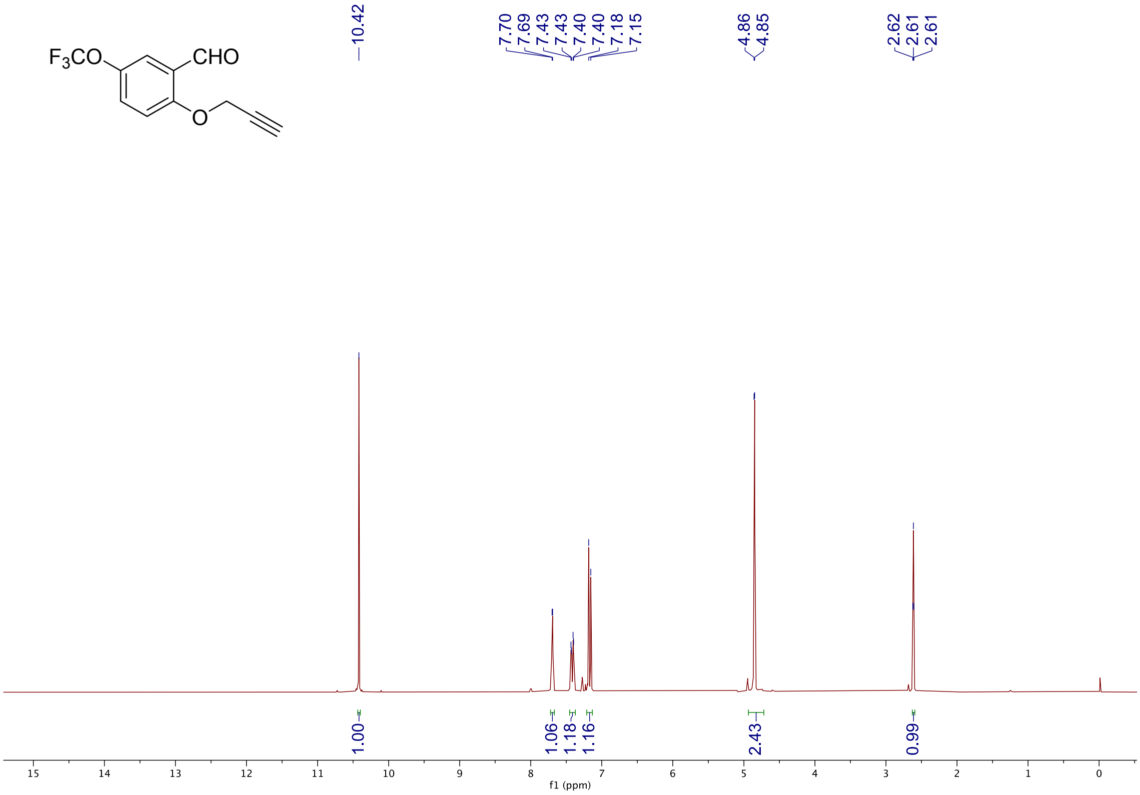

Figure S4. ^{1}H NMR spectrum of *O*-propargylsalicylaldehyde **5d** (300 MHz, $CDCl_3$).

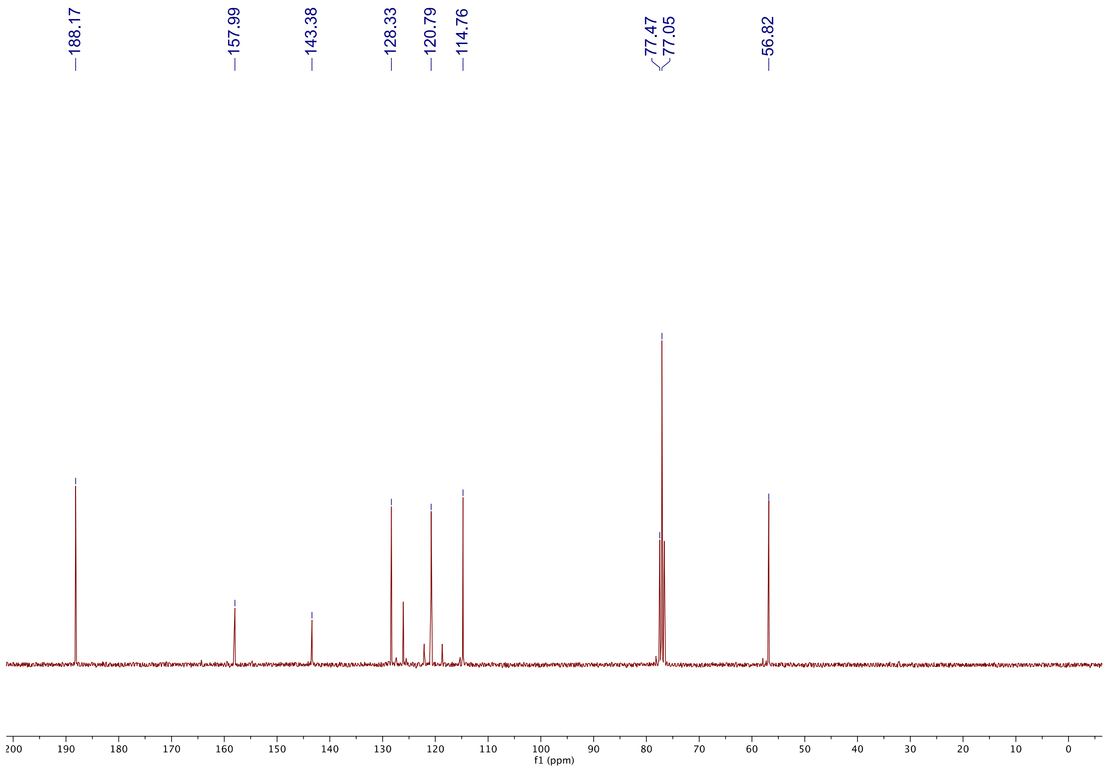

Figure S5. ^{13}C NMR spectrum of *O*-propargylsalicylaldehyde **5d** (75 MHz, $CDCl_3$).

Compound 7h

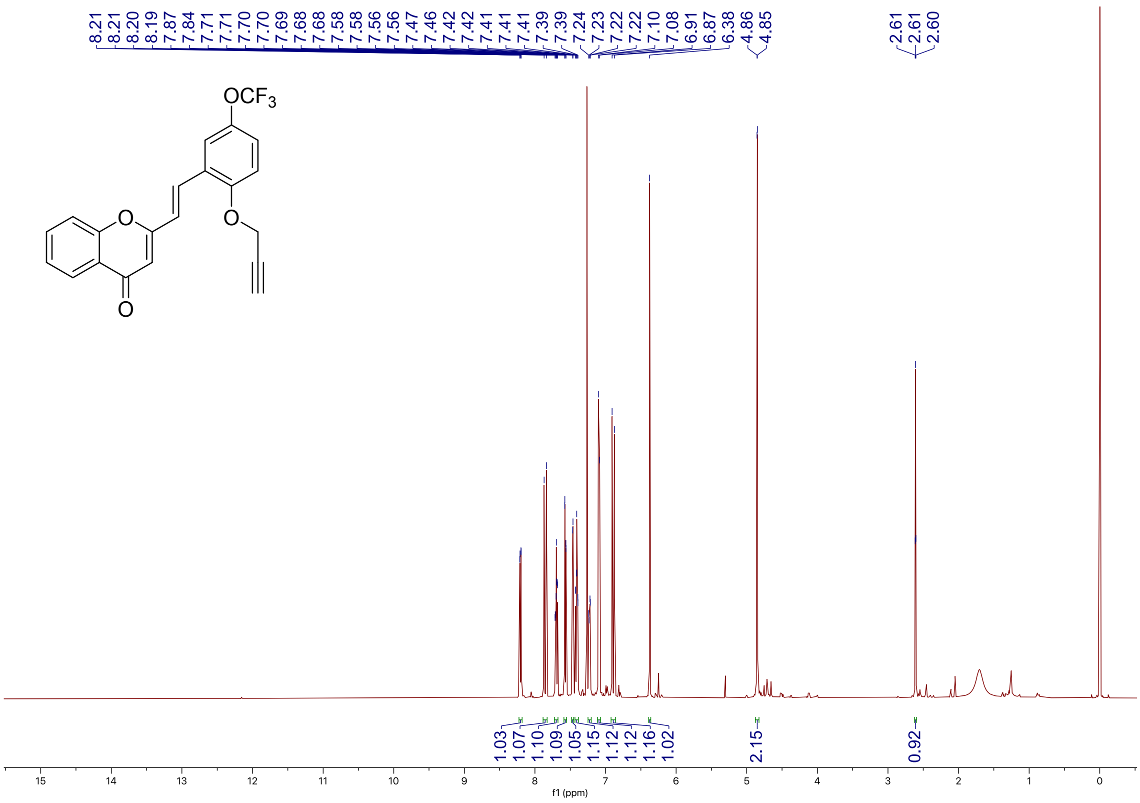

Figure S6. ^{1}H NMR spectrum of (*E*)-2-styrylchromone **7h** (500 MHz, $CDCl_3$).

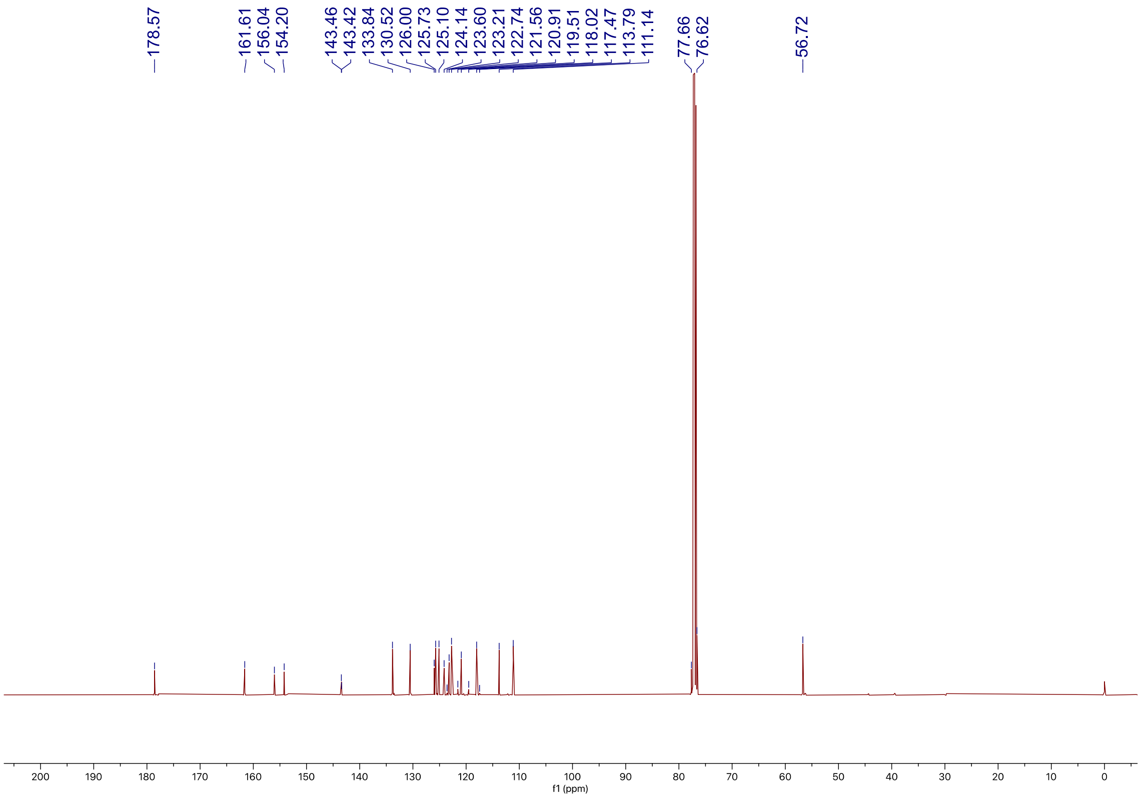

Figure S7. ^{13}C NMR spectrum of (*E*)-2-styrylchromone **7h** (125 MHz, $CDCl_3$).

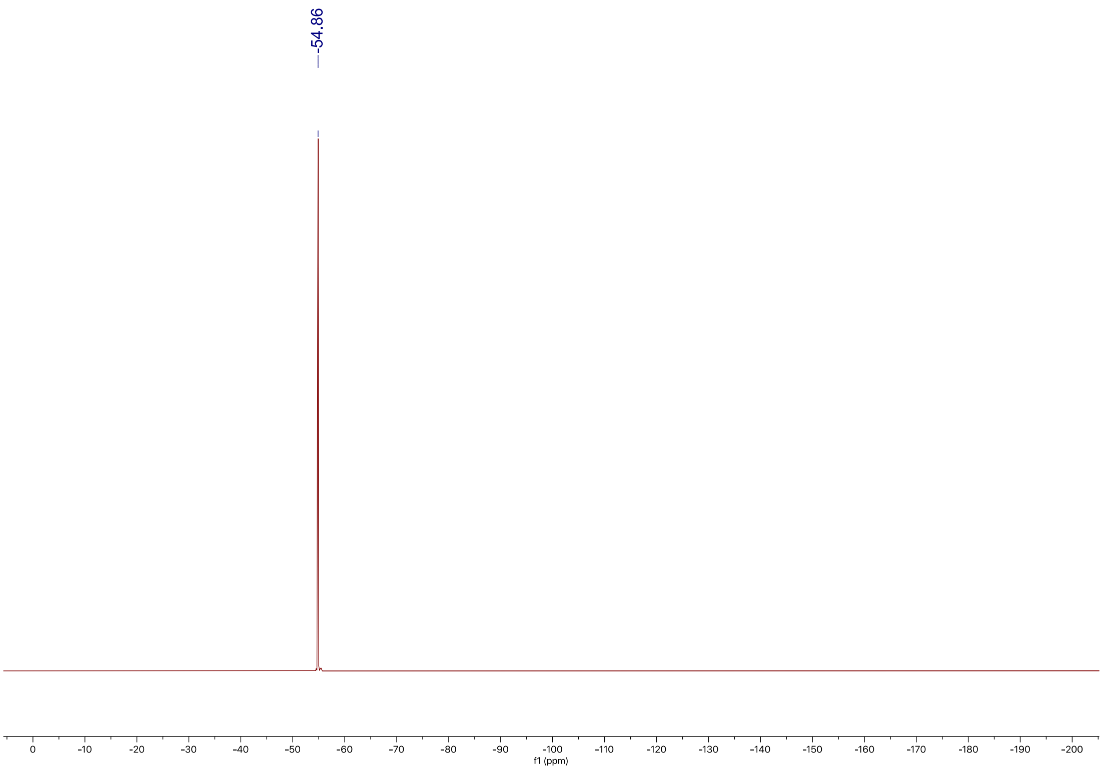

Figure S8. ^{19}F NMR spectrum of (*E*)-2-styrylchromone **7h** (282 MHz, $CDCl_3$).

Compound 7i

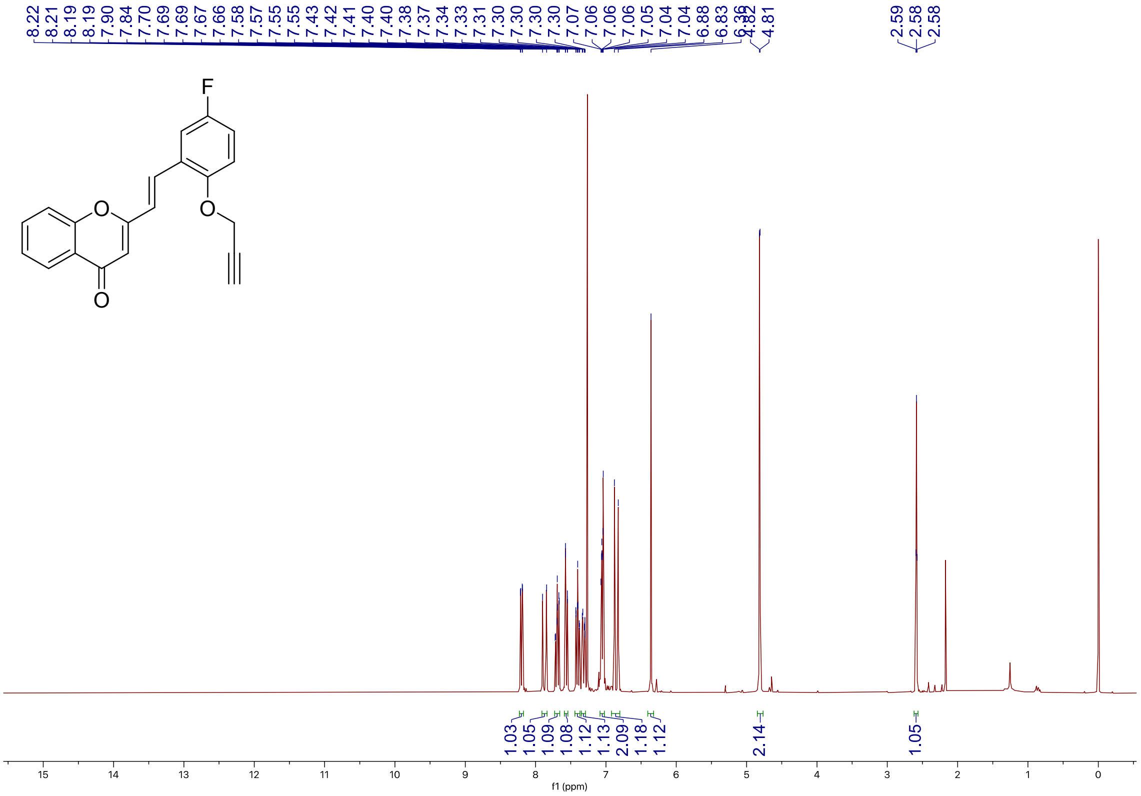

Figure S9. ^{1}H NMR spectrum of (*E*)-2-styrylchromone **7i** (300 MHz, $CDCl_3$).

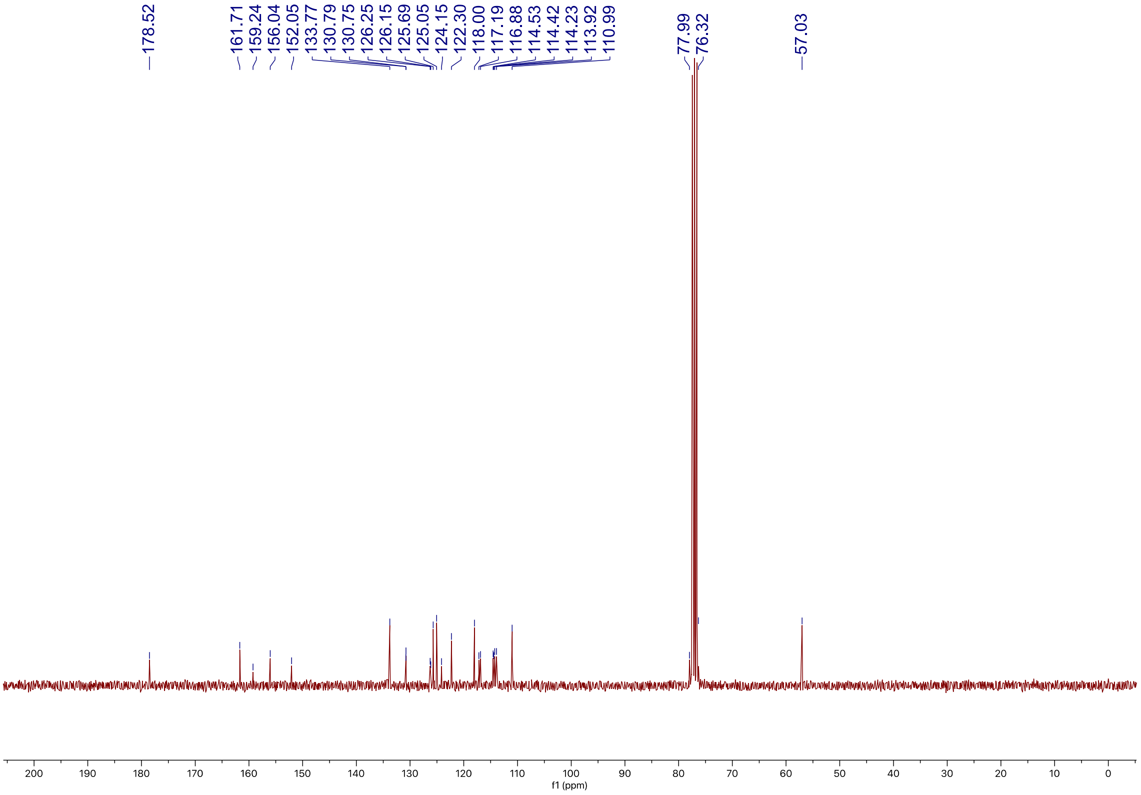

Figure S10. ^{13}C NMR spectrum of (*E*)-2-styrylchromone **7i** (75 MHz, $CDCl_3$).

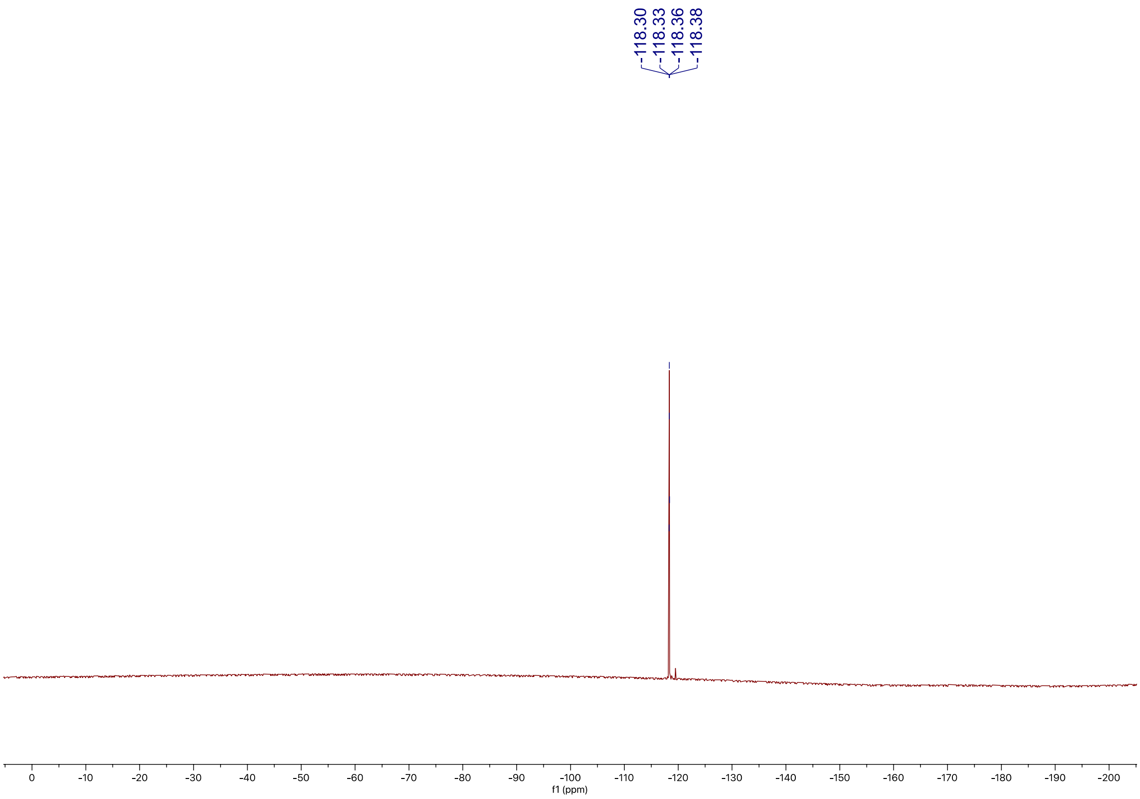

Figure S11. ^{19}F NMR spectrum of (*E*)-2-styrylchromone **7i** (282 MHz, $CDCl_3$).

Compound 7j

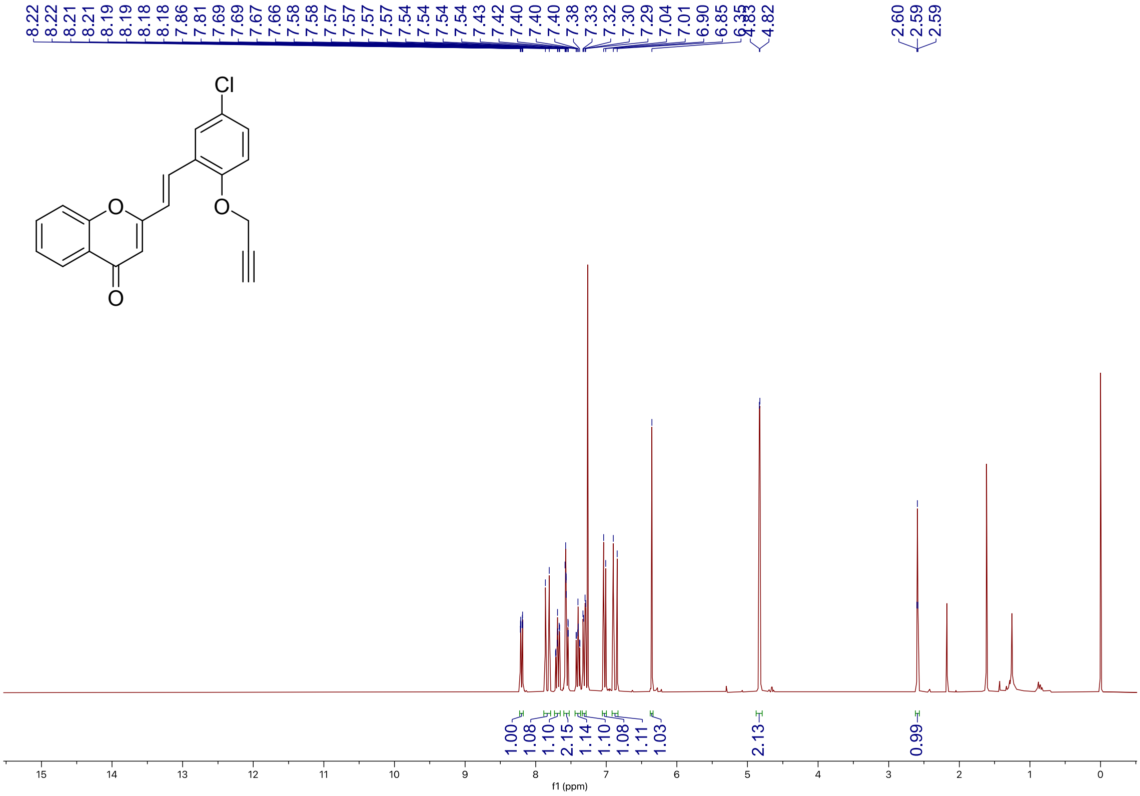

Figure S12. ^{1}H NMR spectrum of (*E*)-2-styrylchromone **7j** (300 MHz, $CDCl_3$).

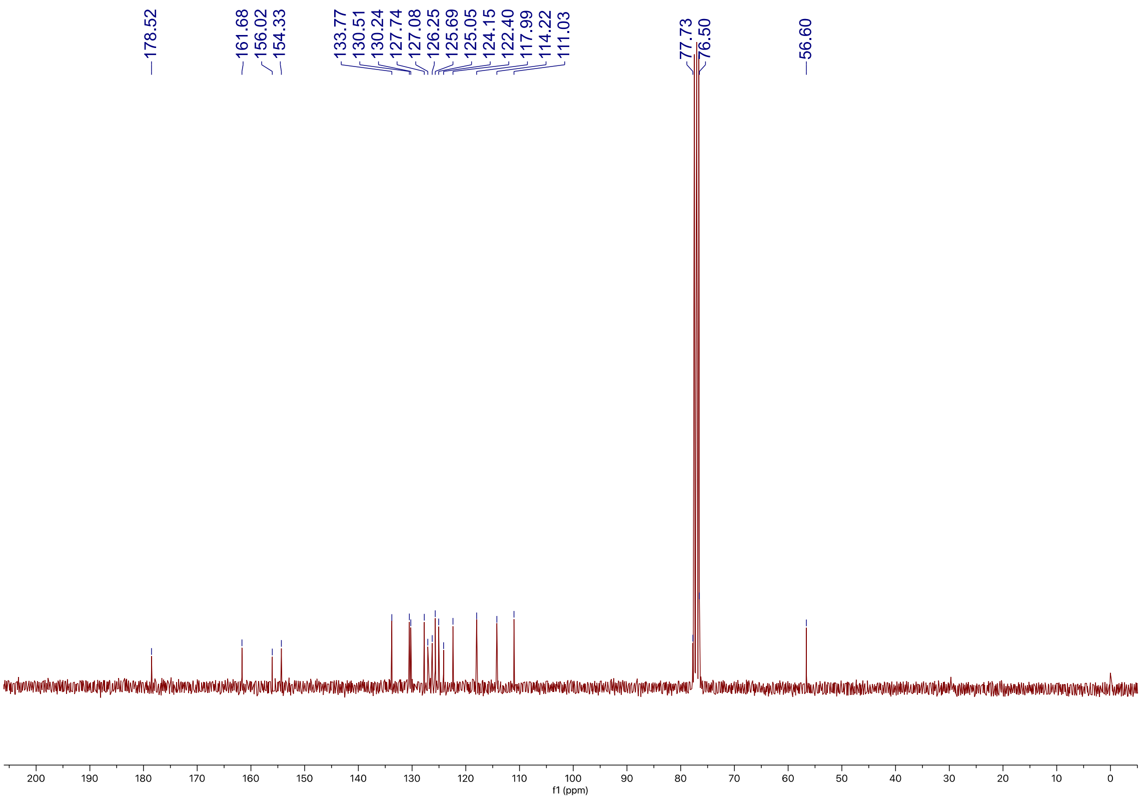

Figure S13. ^{13}C NMR spectrum of (*E*)-2-styrylchromone **7j** (75 MHz, $CDCl_3$).

Compound 7k

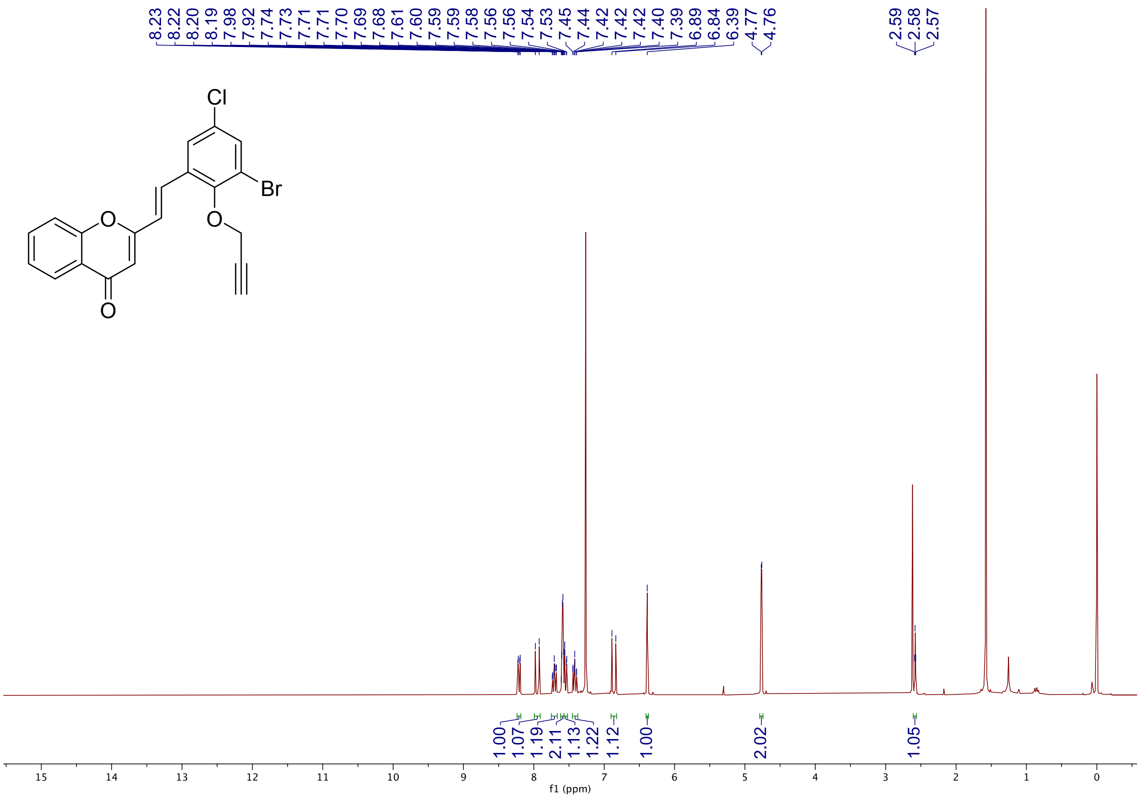

Figure S14. ^{1}H NMR spectrum of (*E*)-2-styrylchromone **7k** (300 MHz, $CDCl_3$).

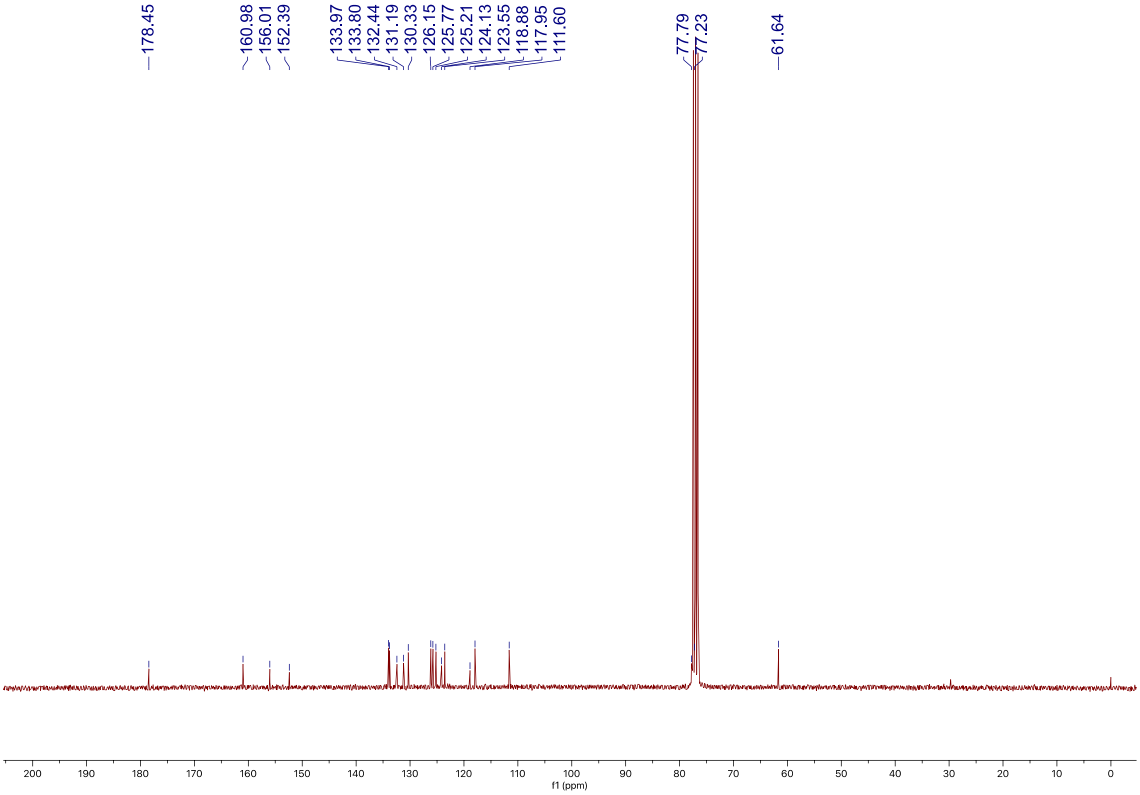

Figure S15. ^{13}C NMR spectrum of (*E*)-2-styrylchromone **7k** (75 MHz, $CDCl_3$).

Compound 7m

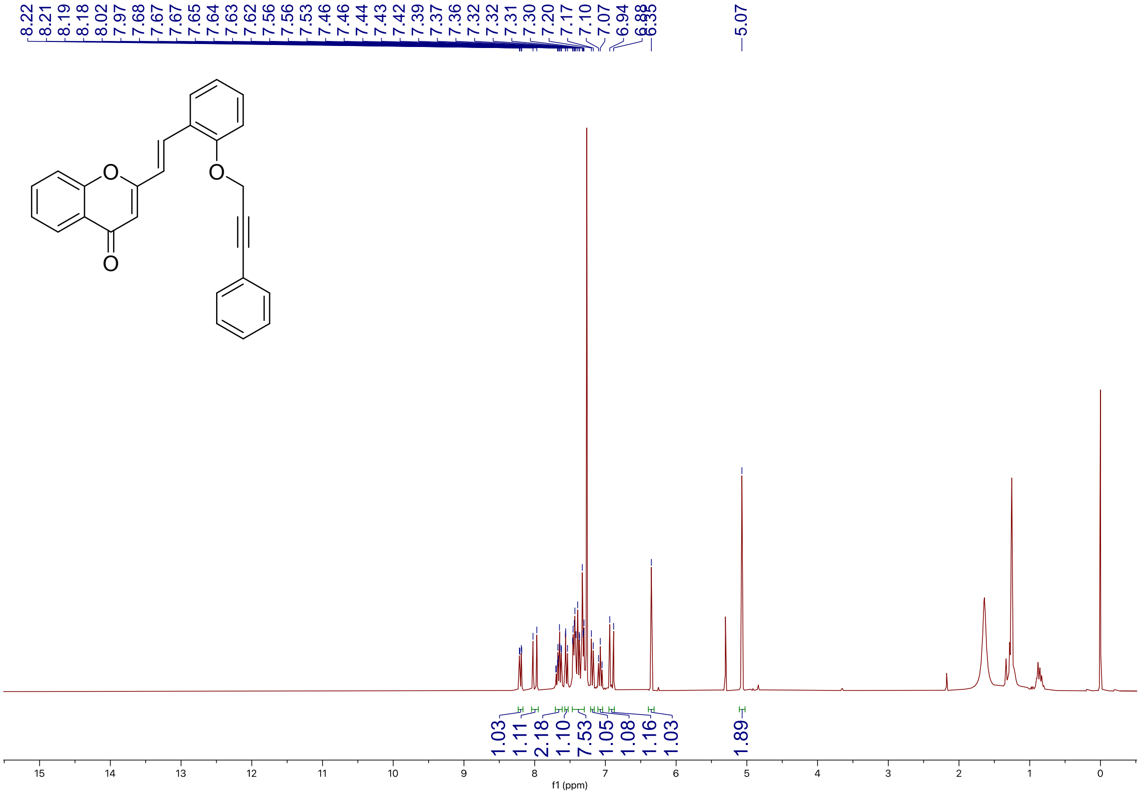

Figure S16. ^{1}H NMR spectrum of (*E*)-2-styrylchromone **7m** (300 MHz, $CDCl_3$).

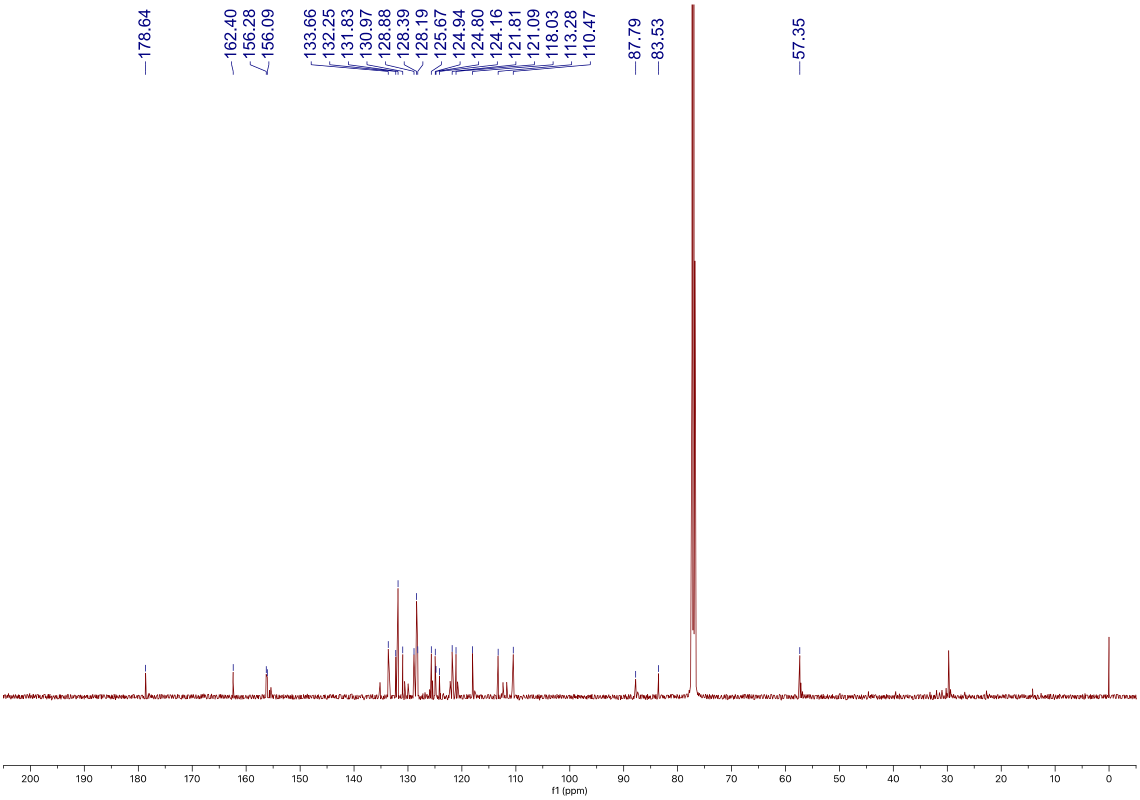

Figure S17. ^{13}C NMR spectrum of (*E*)-2-styrylchromone **7m** (125 MHz, $CDCl_3$).

Compound 7n

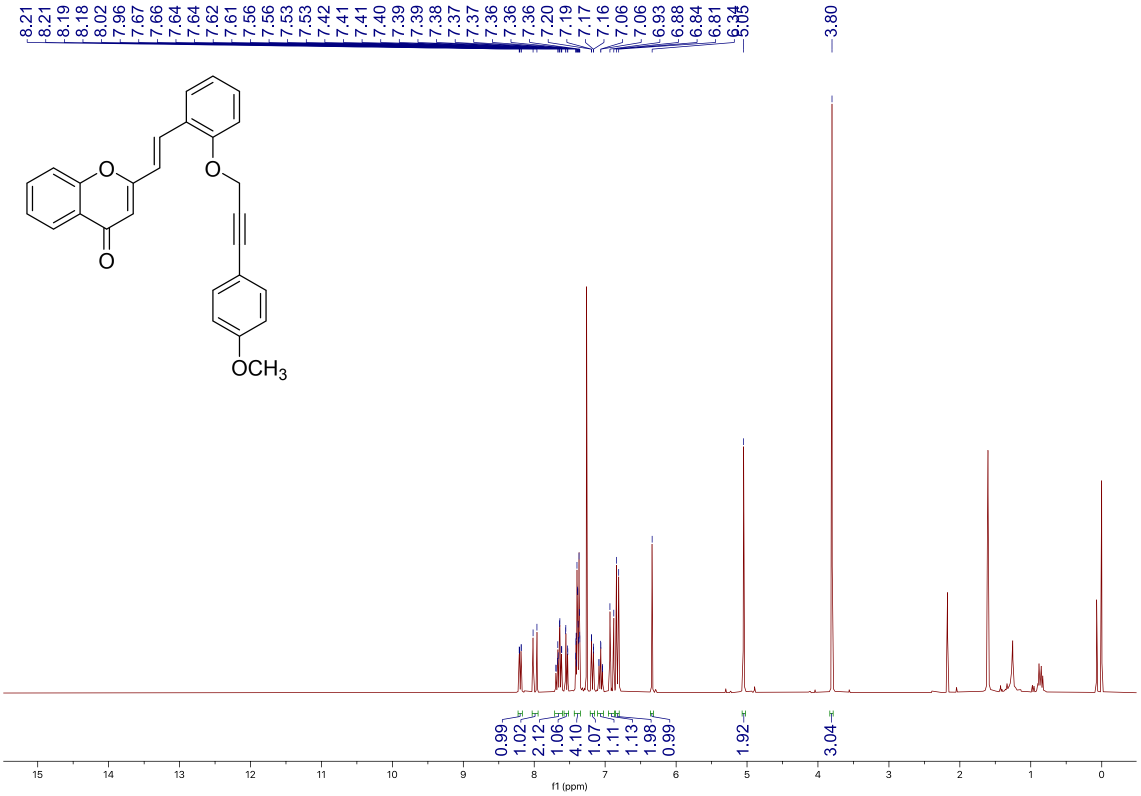

Figure S18. ^{1}H NMR spectrum of (*E*)-2-styrylchromone **7n** (300 MHz, $CDCl_3$).

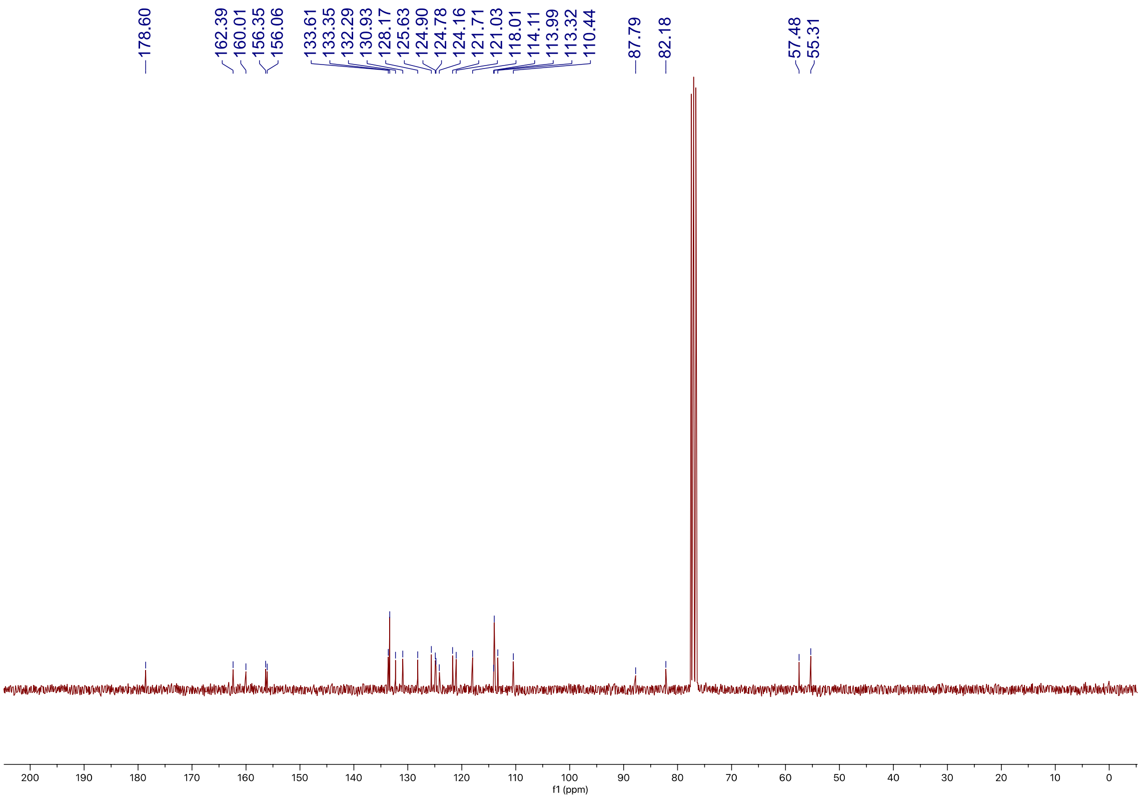

Figure S19. ^{13}C NMR spectrum of (*E*)-2-styrylchromone **7n** (75 MHz, $CDCl_3$).

Compound 7s

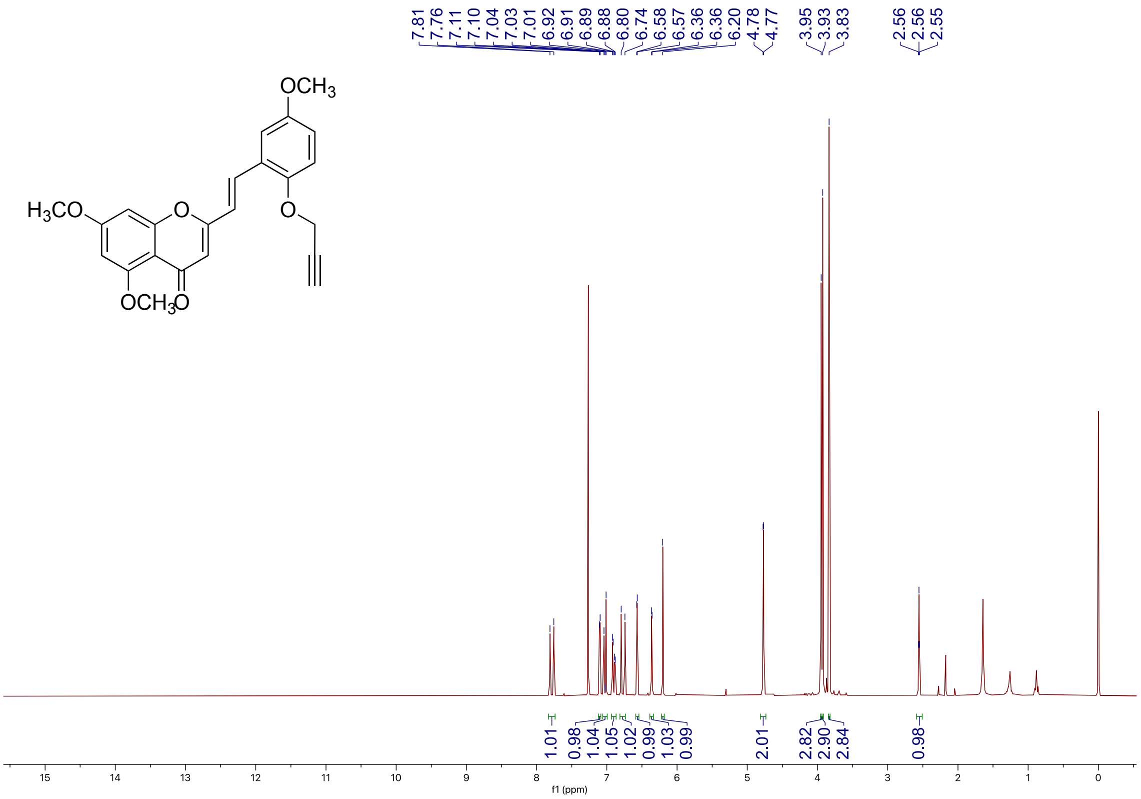

Figure S20. ^{1}H NMR spectrum of (*E*)-2-styrylchromone **7s** (300 MHz, $CDCl_3$).

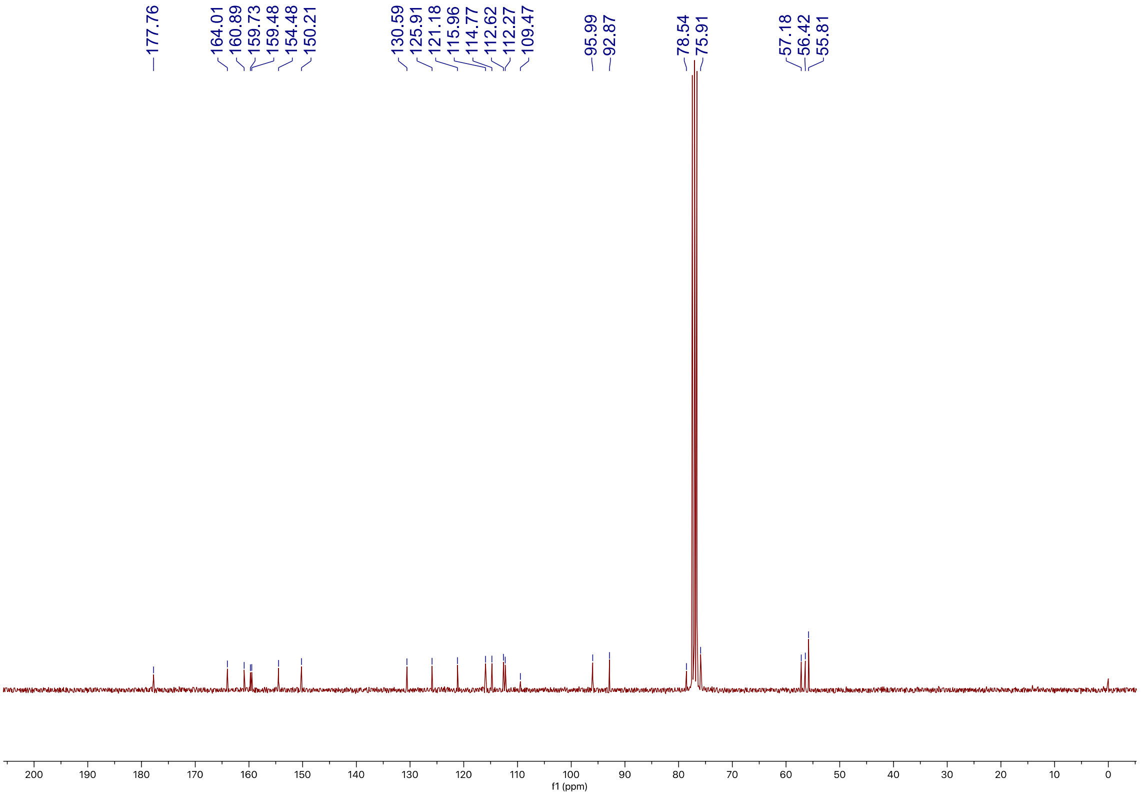

Figure S21. ^{13}C NMR spectrum of (*E*)-2-styrylchromone **7s** (75 MHz, $CDCl_3$).

Compound 7t

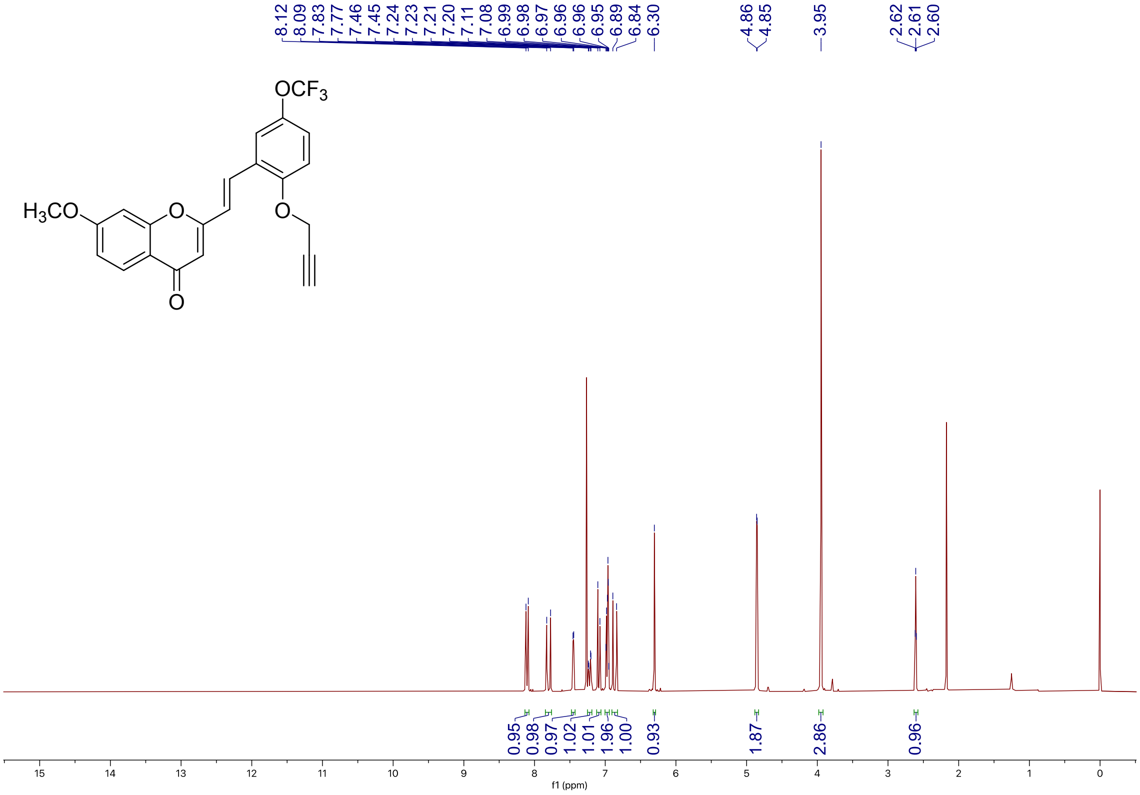

Figure S22. ^{1}H NMR spectrum of (*E*)-2-styrylchromone **7t** (300 MHz, $CDCl_3$).

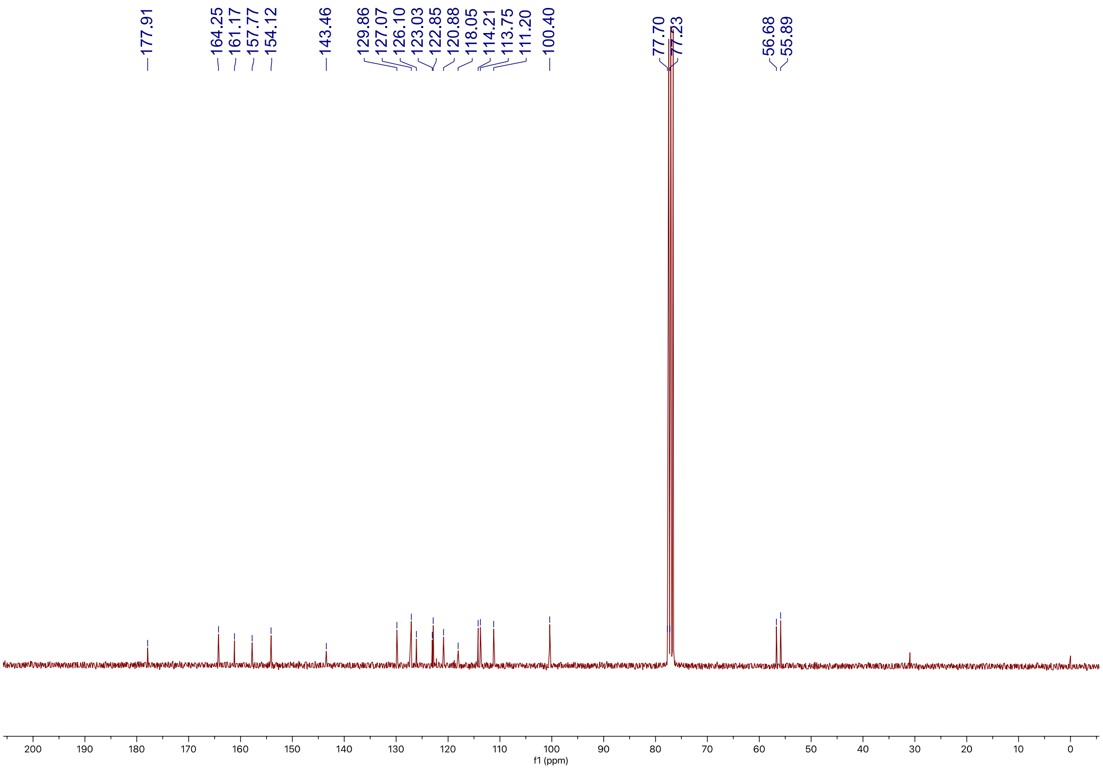

Figure S23. ^{13}C NMR spectrum of (*E*)-2-styrylchromone **7t** (75 MHz, $CDCl_3$).

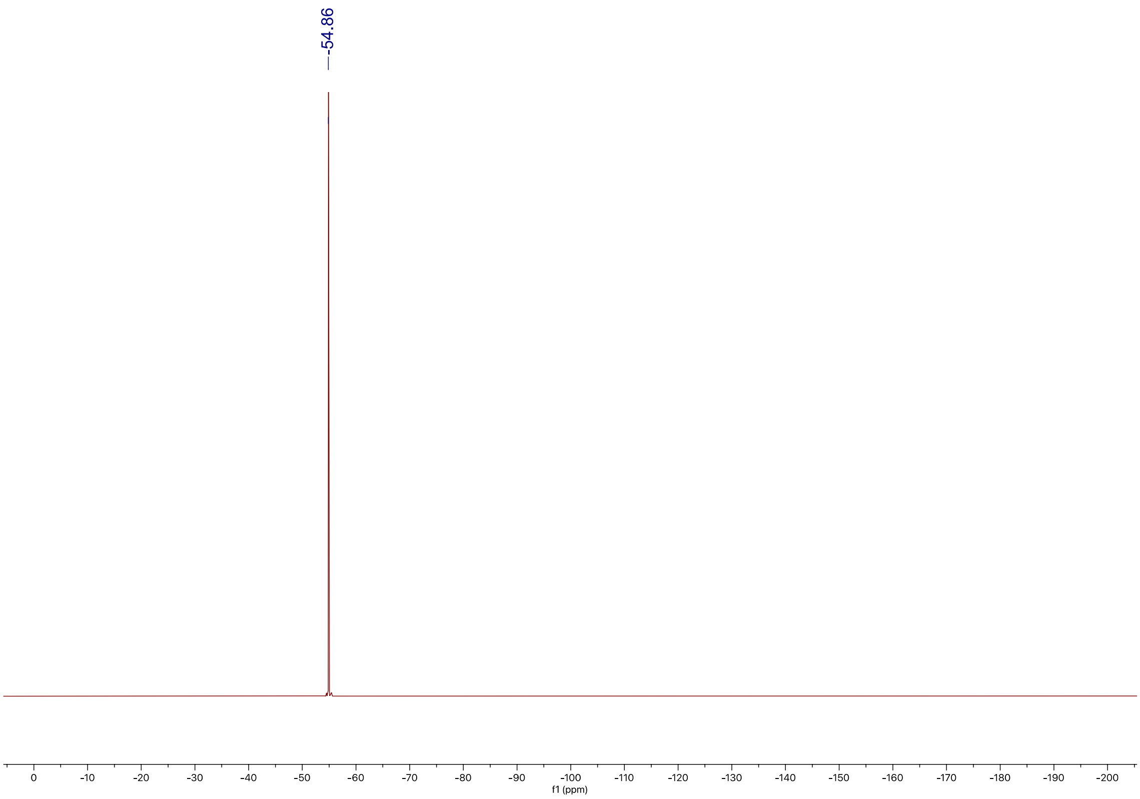

Figure S24. ^{19}F NMR spectrum of (*E*)-2-styrylchromone **7t** (282 MHz, $CDCl_3$).

Compound 7u

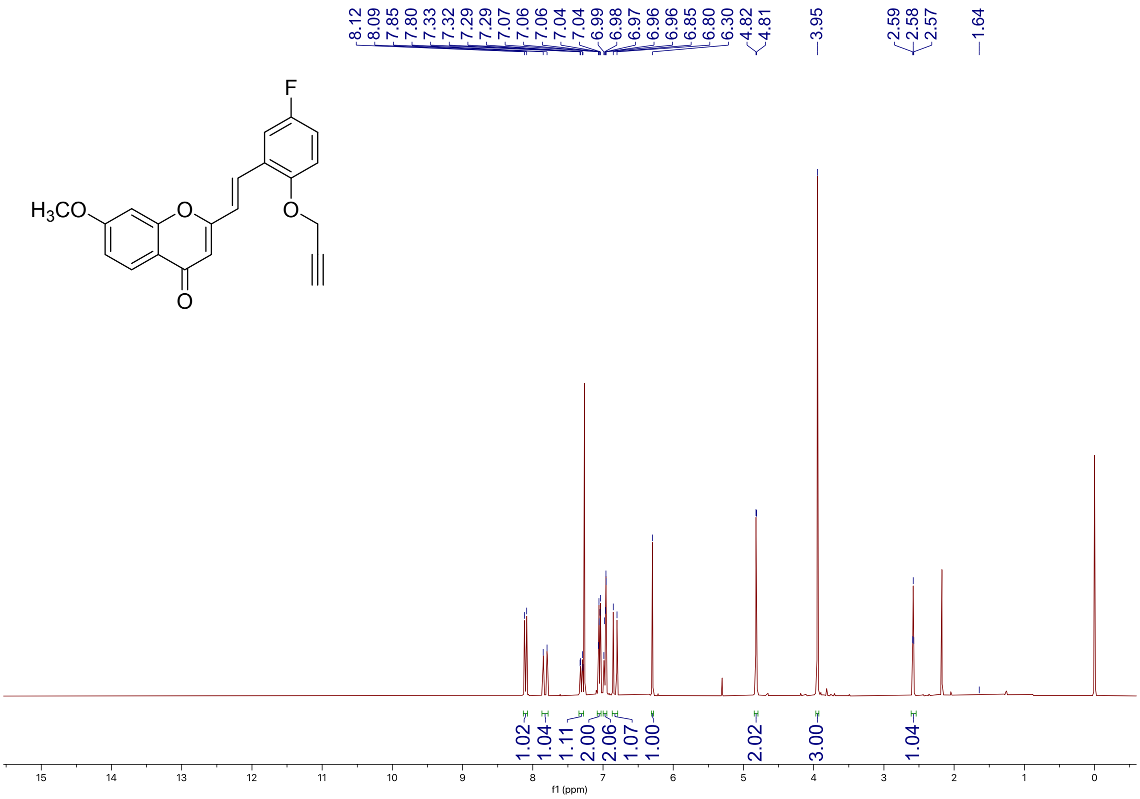

Figure S25. ^{1}H NMR spectrum of (*E*)-2-styrylchromone **7u** (300 MHz, $CDCl_3$).

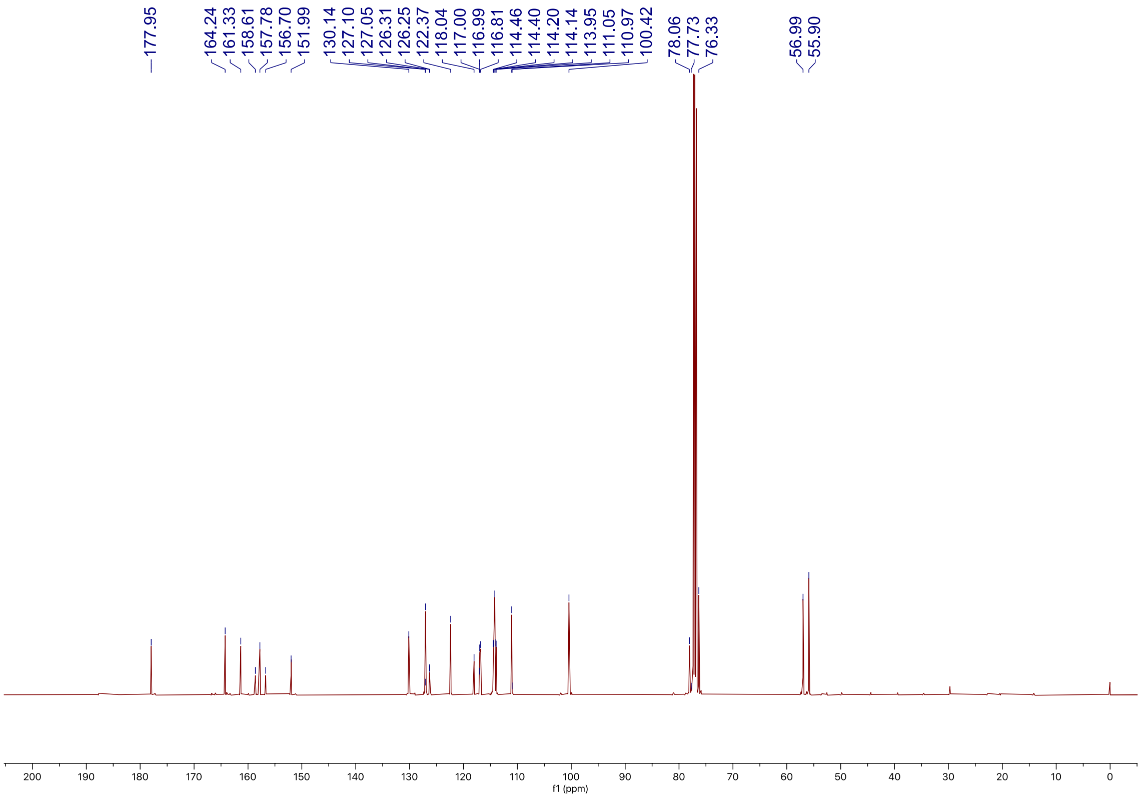

Figure S26. ^{13}C NMR spectrum of (*E*)-2-styrylchromone **7u** (125 MHz, $CDCl_3$).

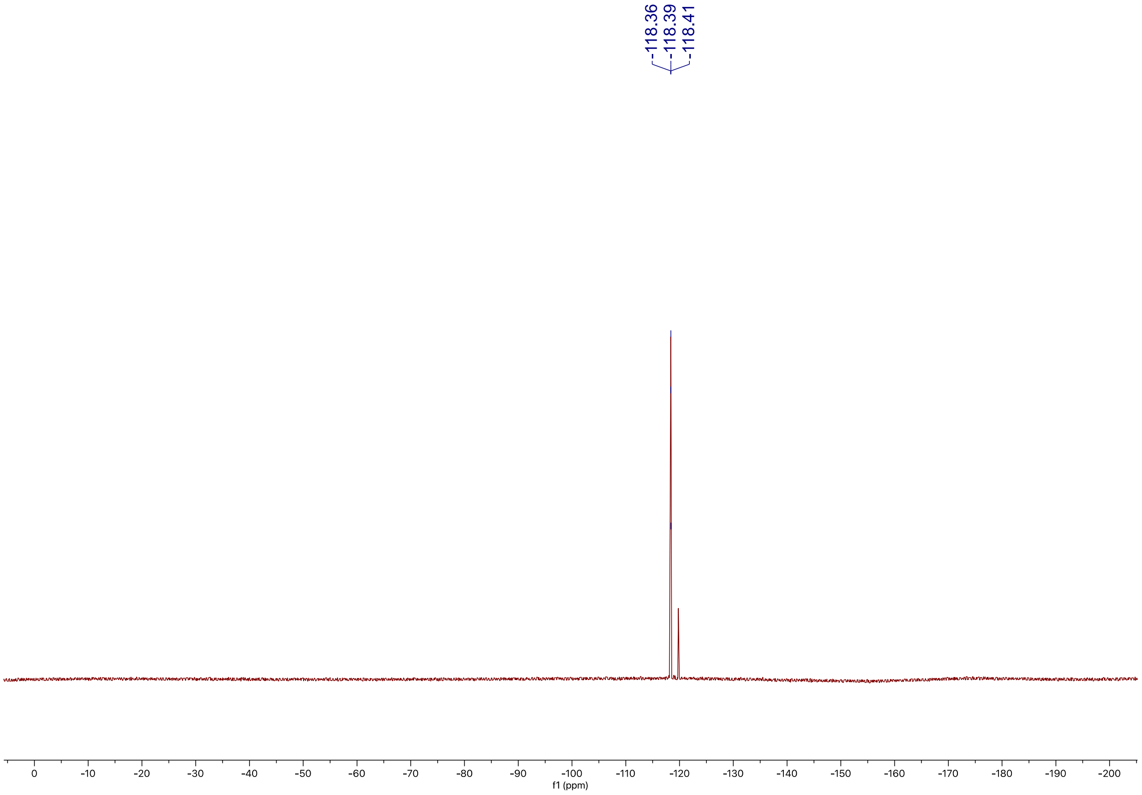

Figure S27. ^{19}F NMR spectrum of (*E*)-2-styrylchromone **7u** (282 MHz, $CDCl_3$).

Compound 7v

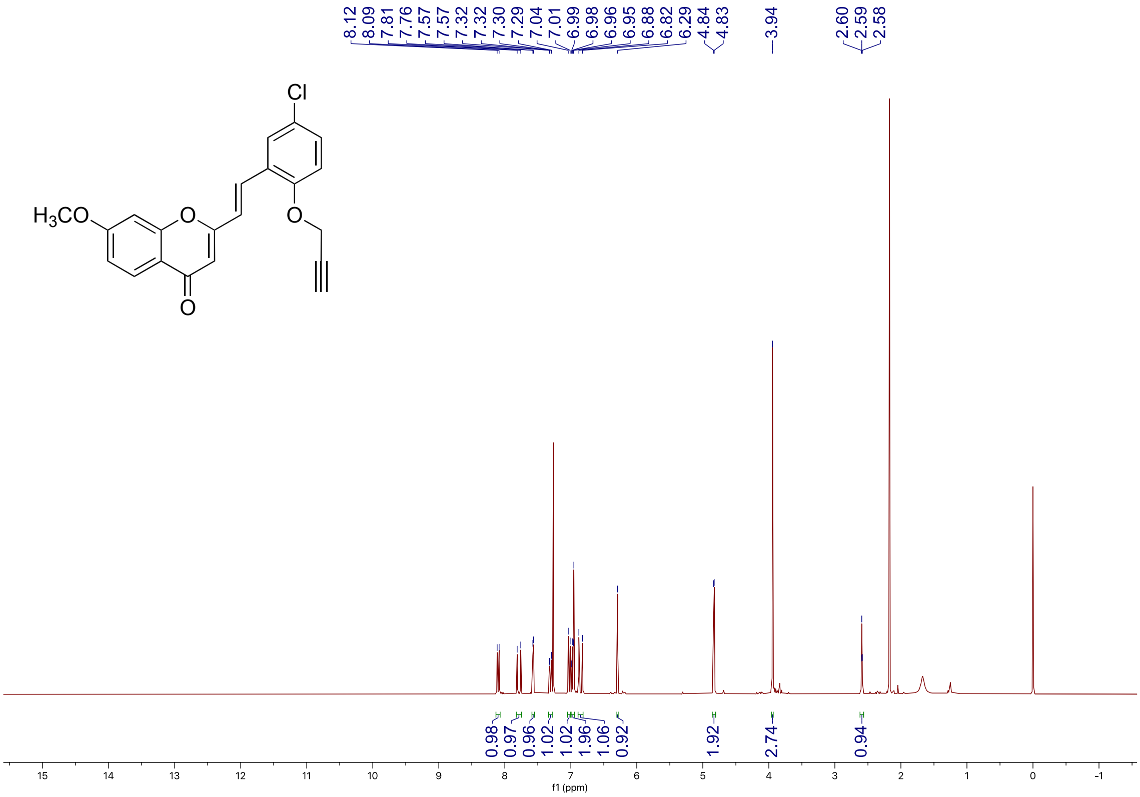

Figure S28. ^{1}H NMR spectrum of (*E*)-2-styrylchromone **7v** (300 MHz, $CDCl_3$).

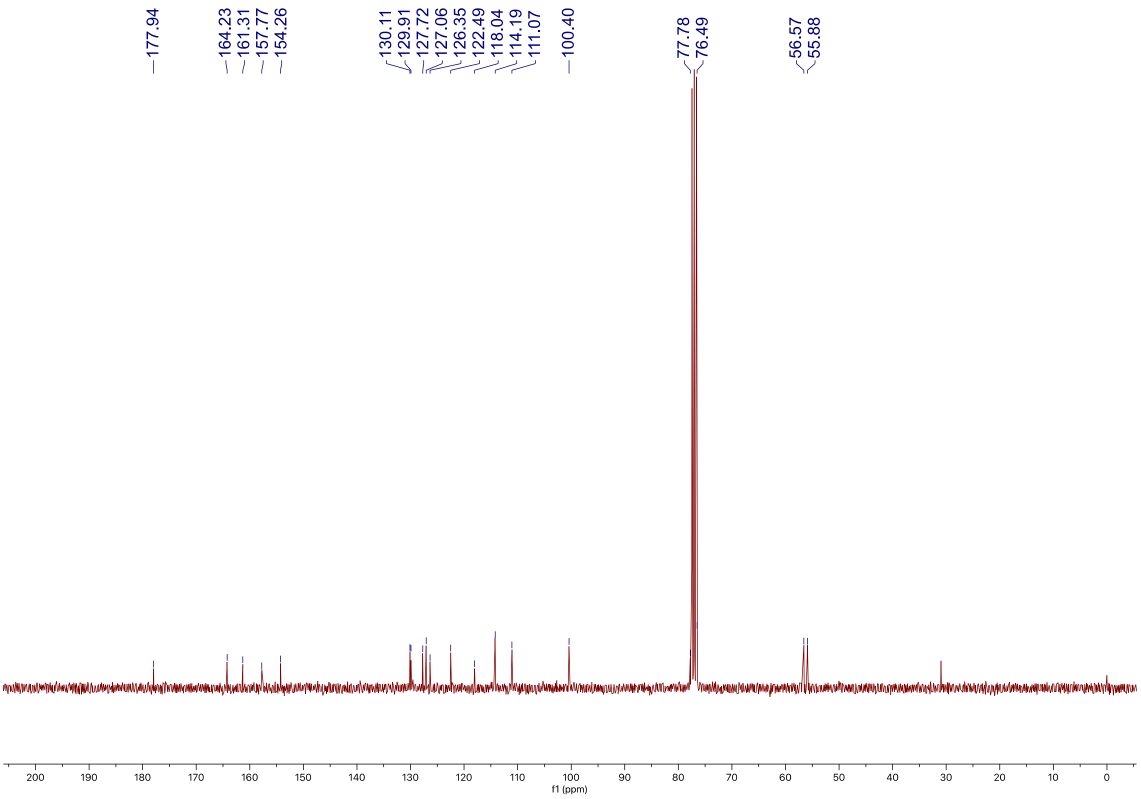

Figure S29. ^{13}C NMR spectrum of (*E*)-2-styrylchromone **7v** (75 MHz, $CDCl_3$).

Compound 7w

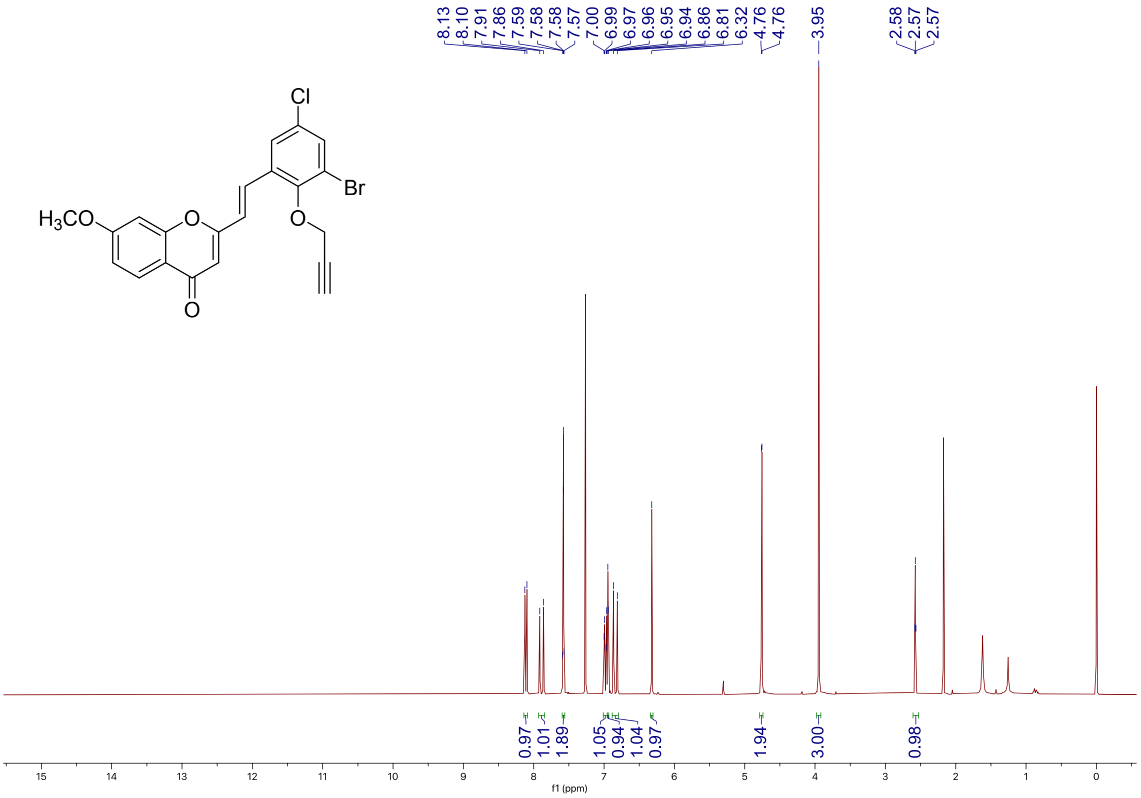

Figure S30. ^{1}H NMR spectrum of (*E*)-2-styrylchromone **7w** (300 MHz, $CDCl_3$).

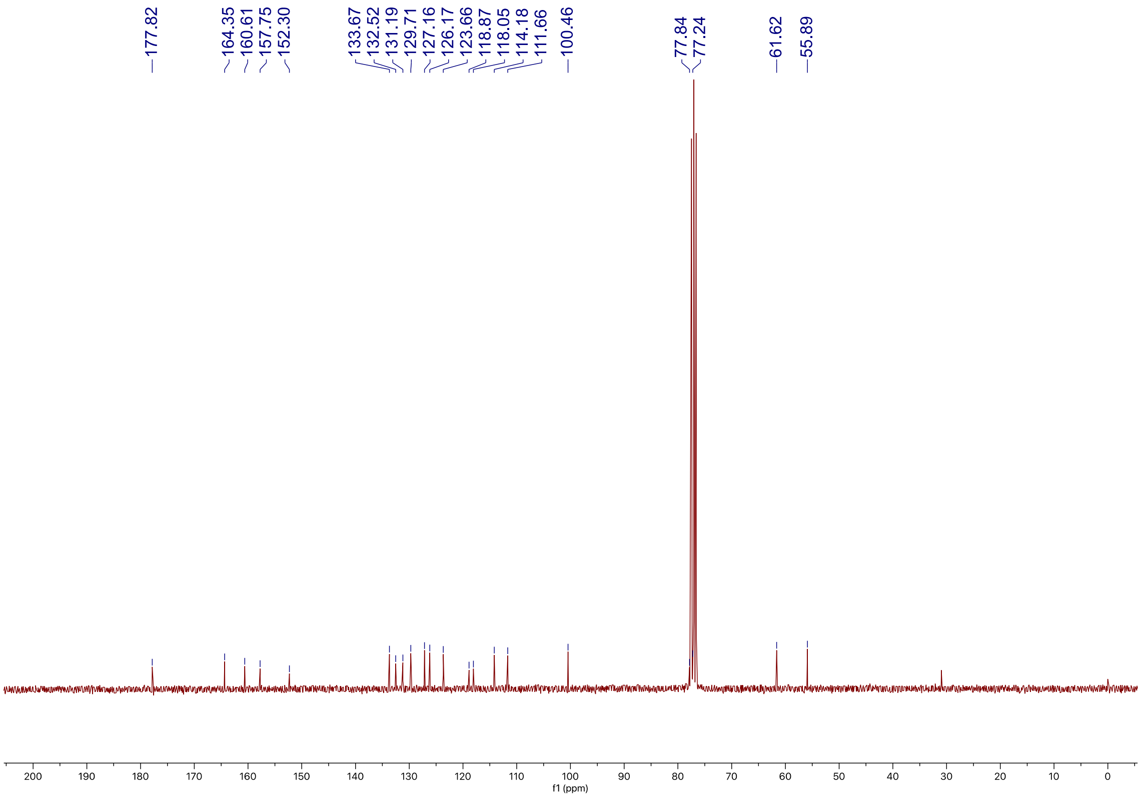

Figure S31. ^{13}C NMR spectrum of (*E*)-2-styrylchromone **7w** (75 MHz, $CDCl_3$).

Compound 8a

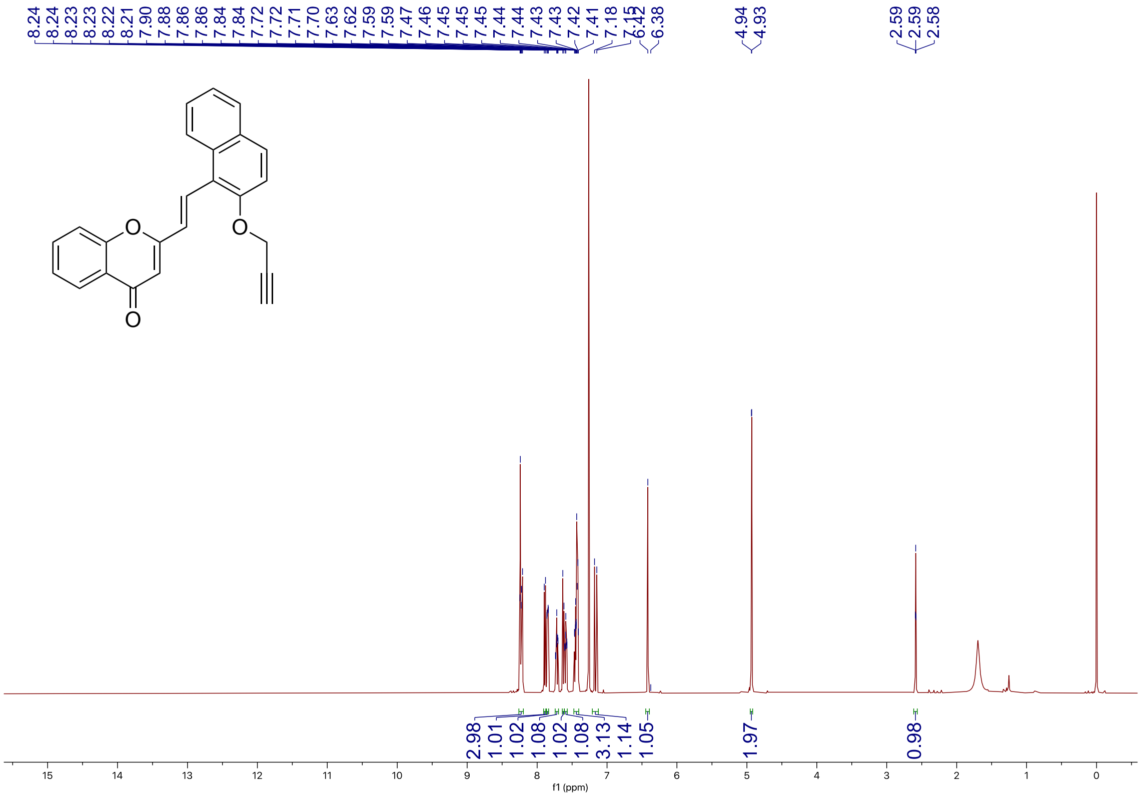

Figure S32. ^{1}H NMR spectrum of (*E*)-2-styrylchromone **8a** (300 MHz, $CDCl_3$).

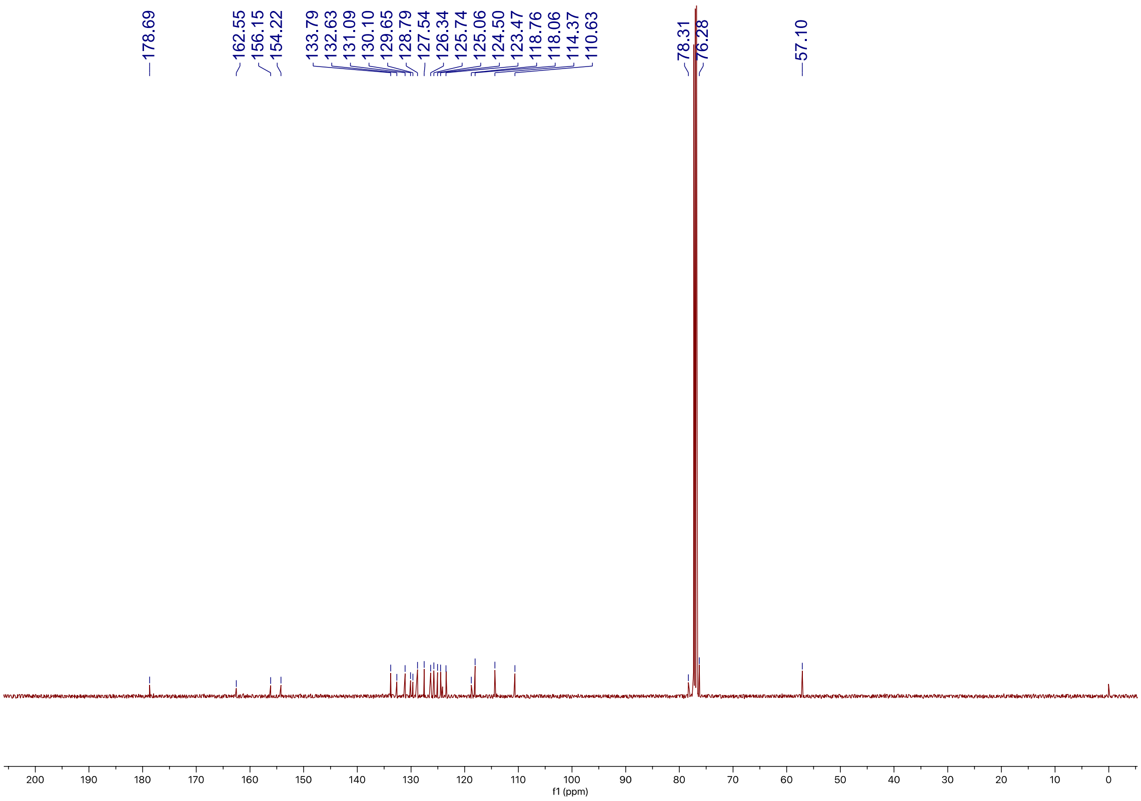

Figure S33. ^{13}C NMR spectrum of (*E*)-2-styrylchromone **8a** (75 MHz, $CDCl_3$).

Compound 8b

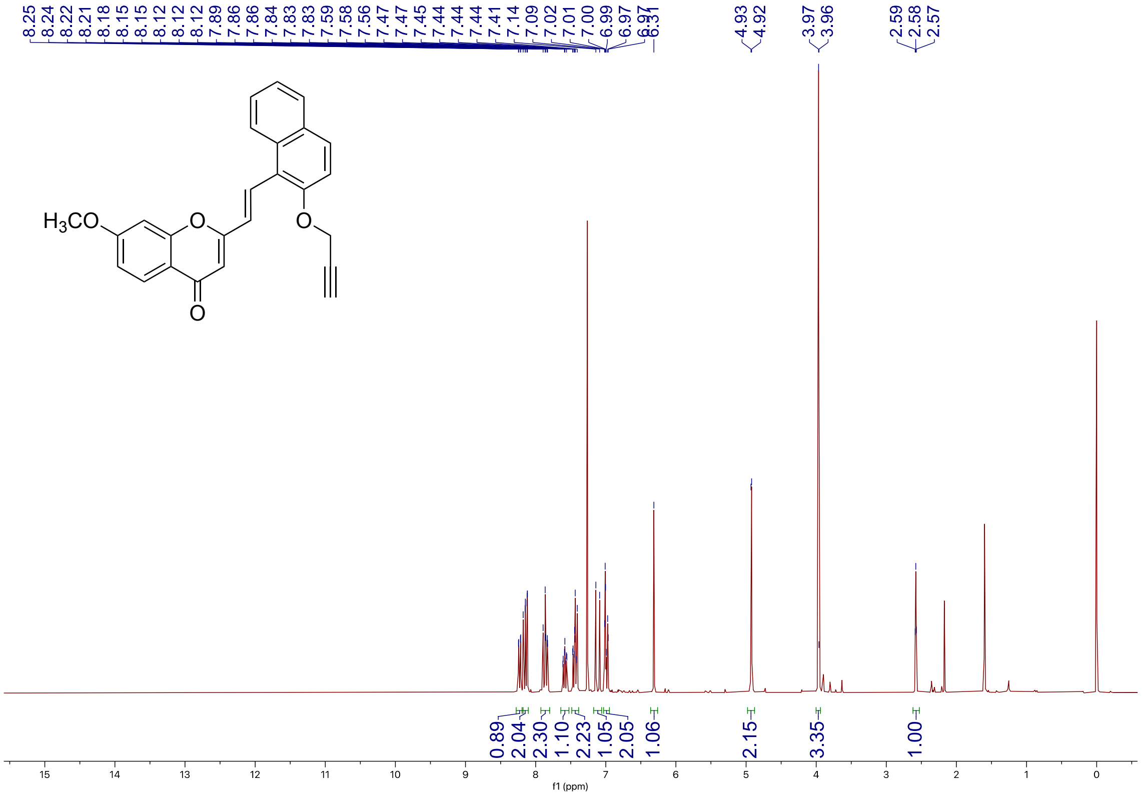

Figure S34. ^{1}H NMR spectrum of (*E*)-2-styrylchromone **8b** (500 MHz, $CDCl_3$).

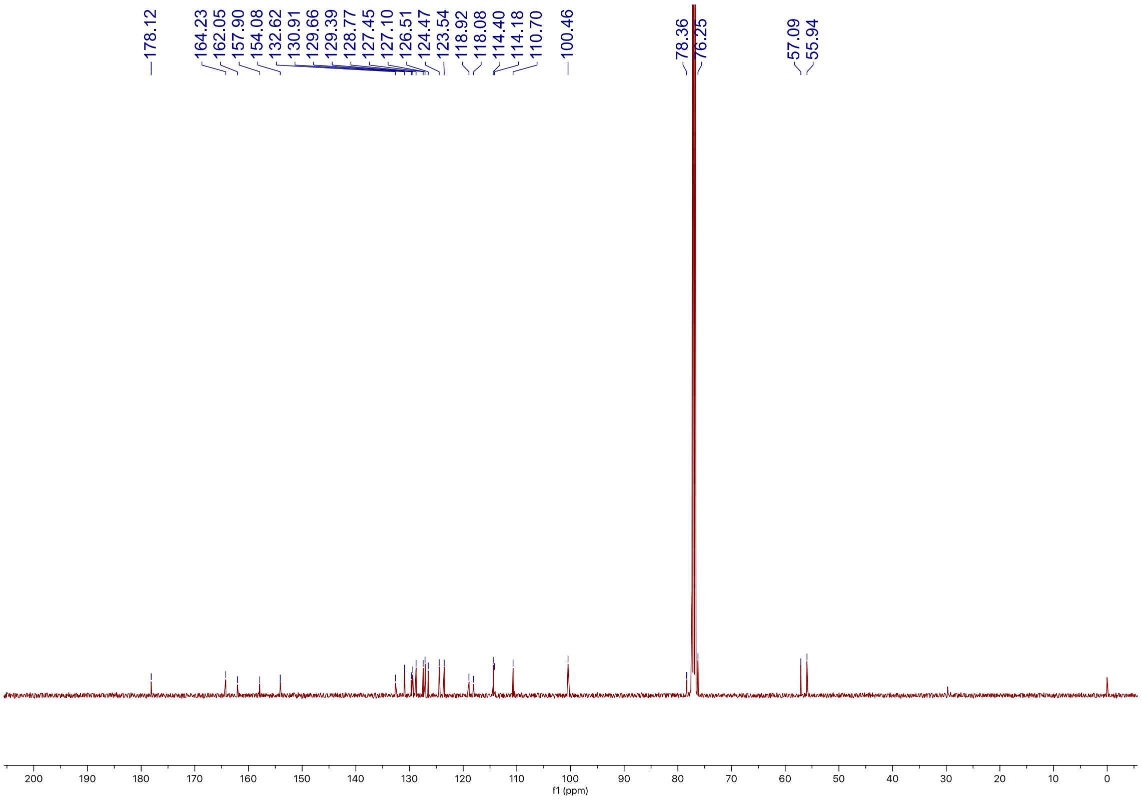

Figure S35. ^{13}C NMR spectrum of (*E*)-2-styrylchromone **8b** (125 MHz, $CDCl_3$).

Compound 11h

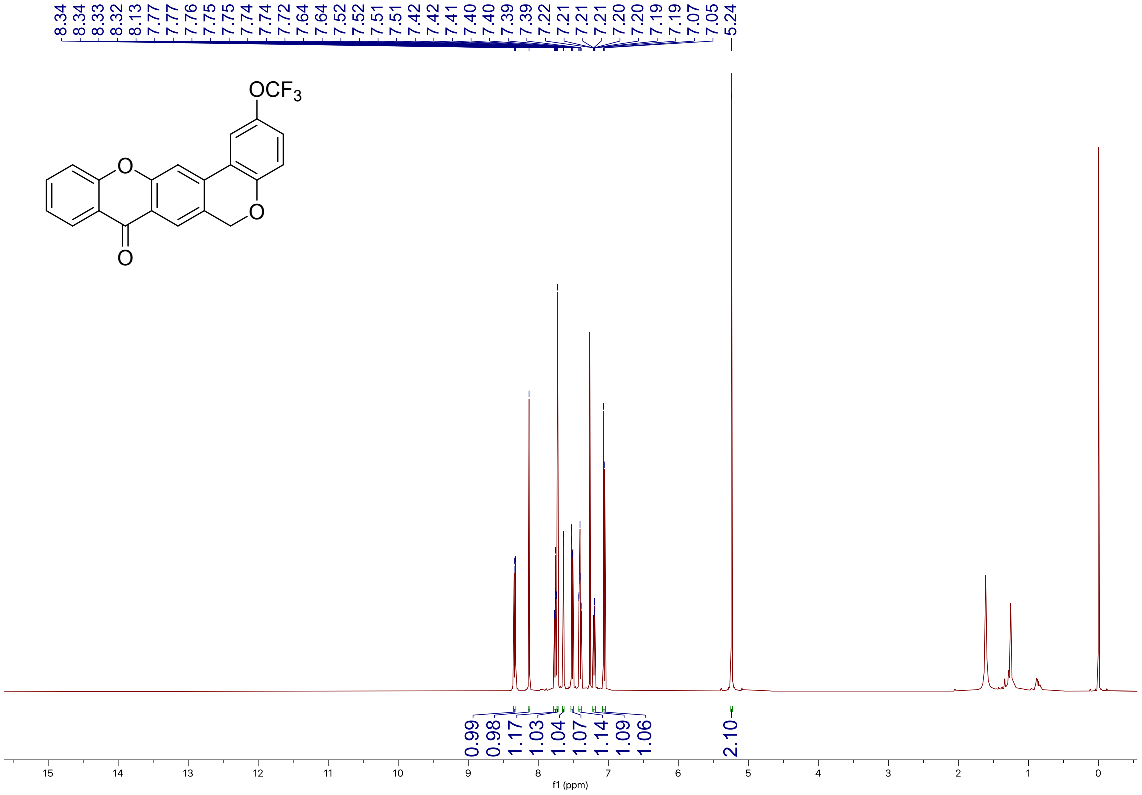

Figure S36. ^{1}H NMR spectrum of chromeno[3,4-*b*]xanthone **11h** (500 MHz, $CDCl_3$).

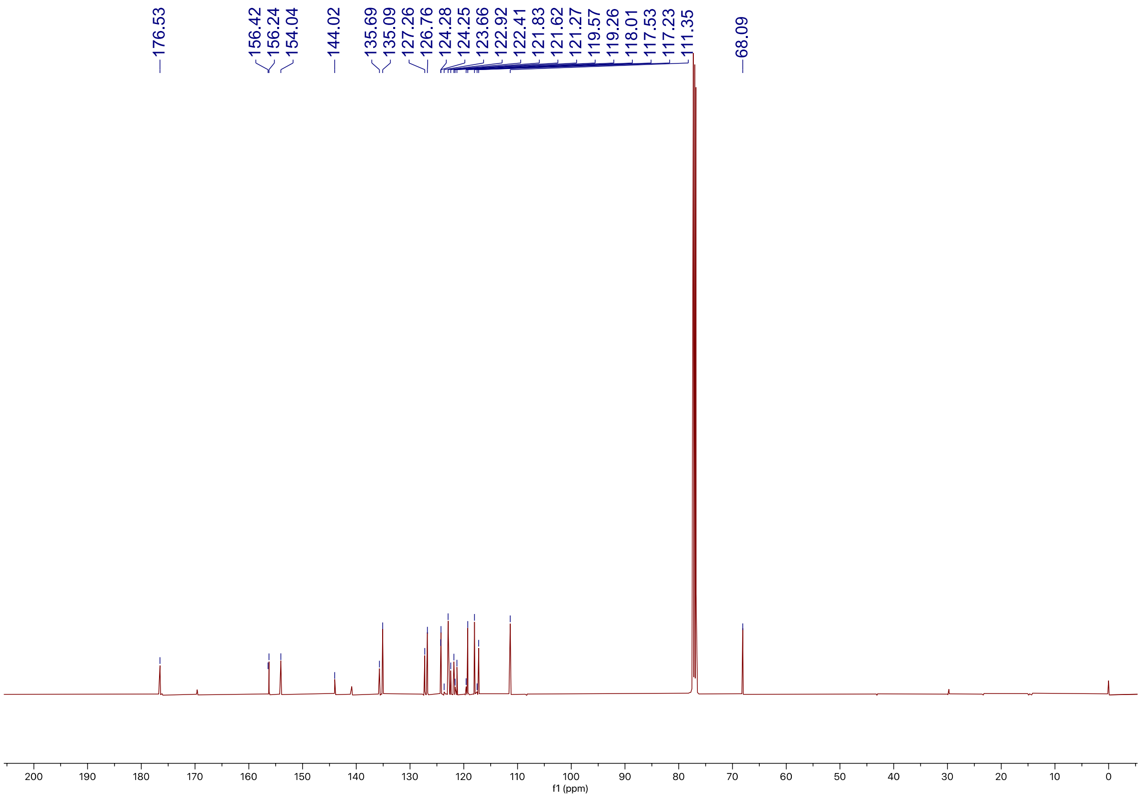

Figure S37. ^{13}C NMR spectrum of chromeno[3,4-*b*]xanthone **11h** (125 MHz, $CDCl_3$).

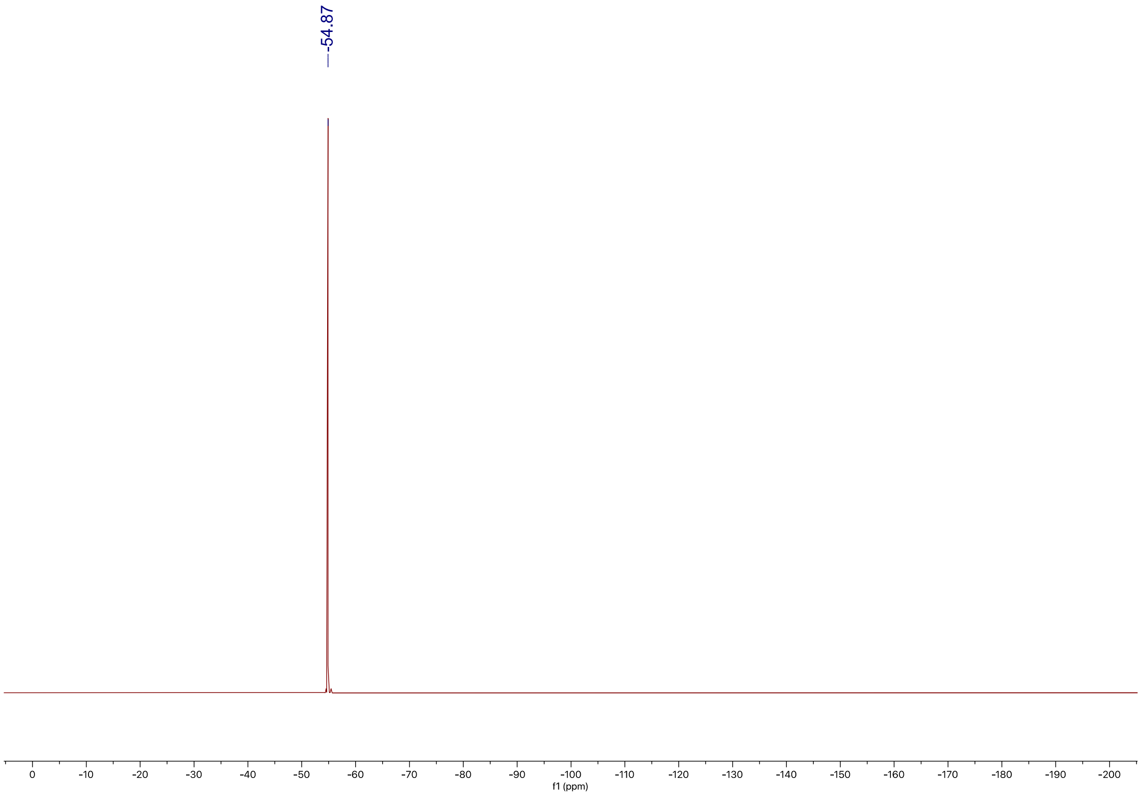

Figure S38. ^{19}F NMR spectrum of (*E*)-2-styrylchromone **11h** (282 MHz, $CDCl_3$).

Compound 11i

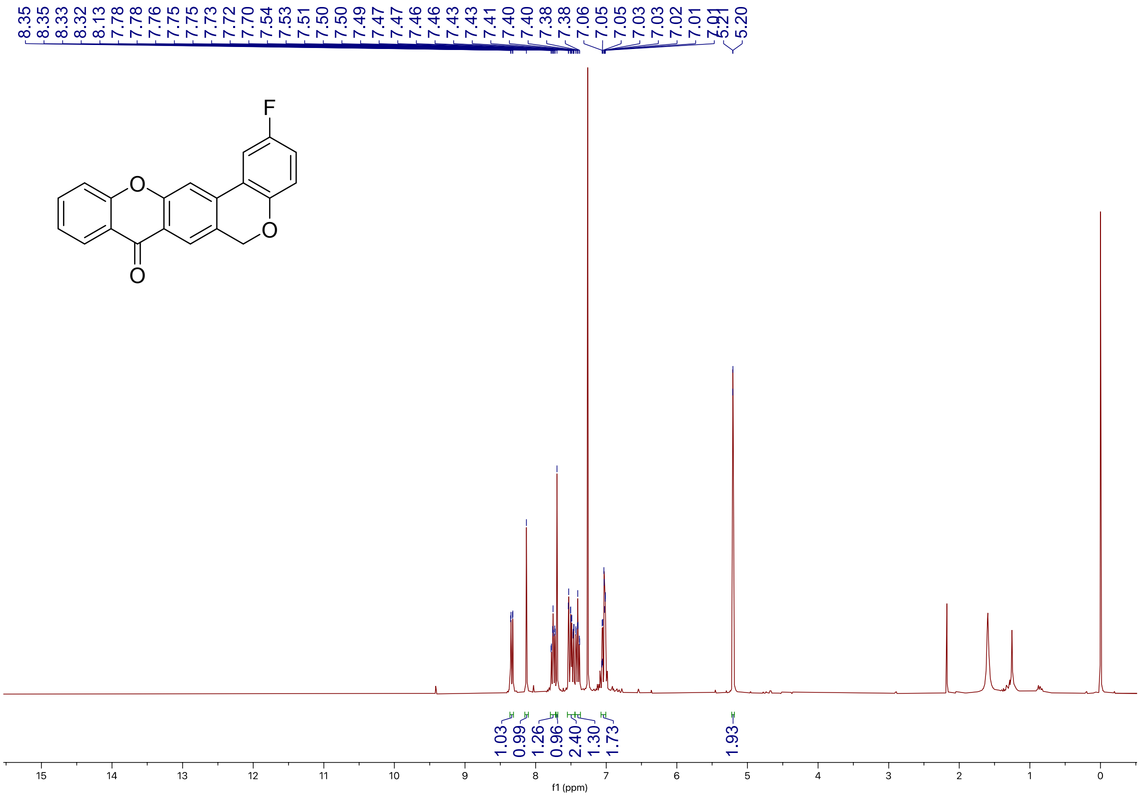

Figure S39. ^{1}H NMR spectrum of chromeno[3,4-*b*]xanthone **11i** (500 MHz, $CDCl_3$).

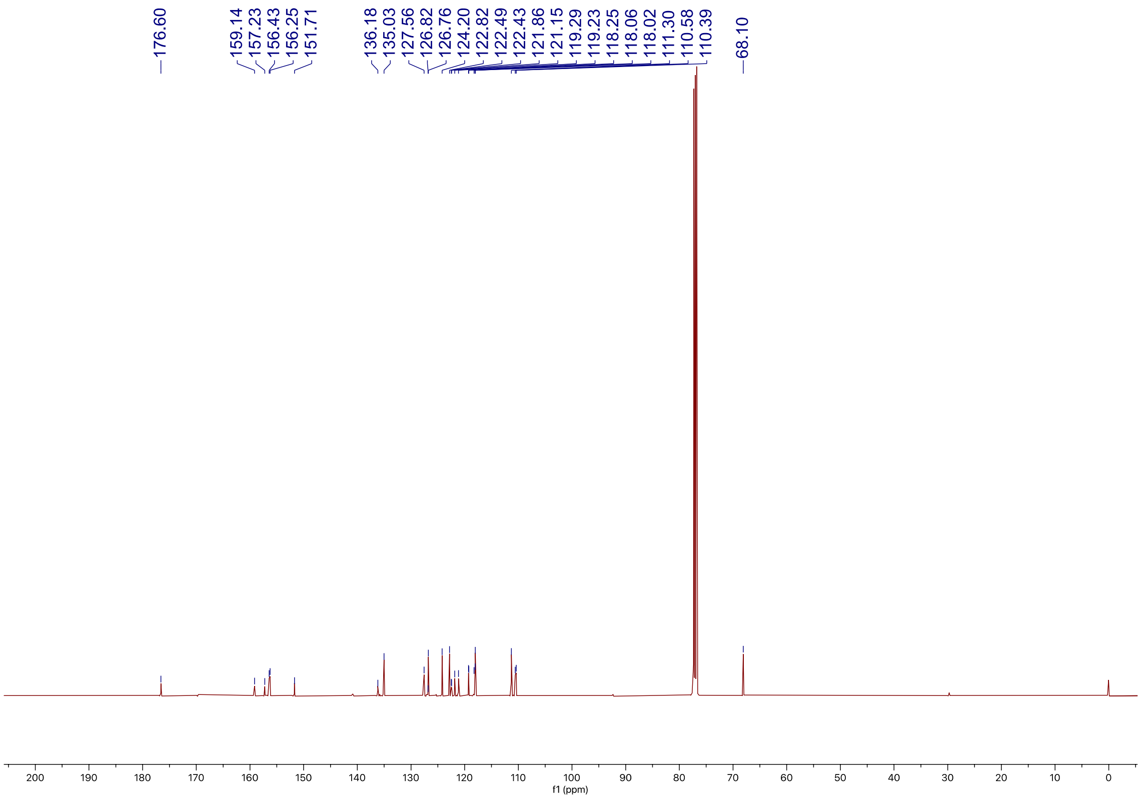

Figure S40. ^{13}C NMR spectrum of chromeno[3,4-*b*]xanthone **11i** (125 MHz, $CDCl_3$).

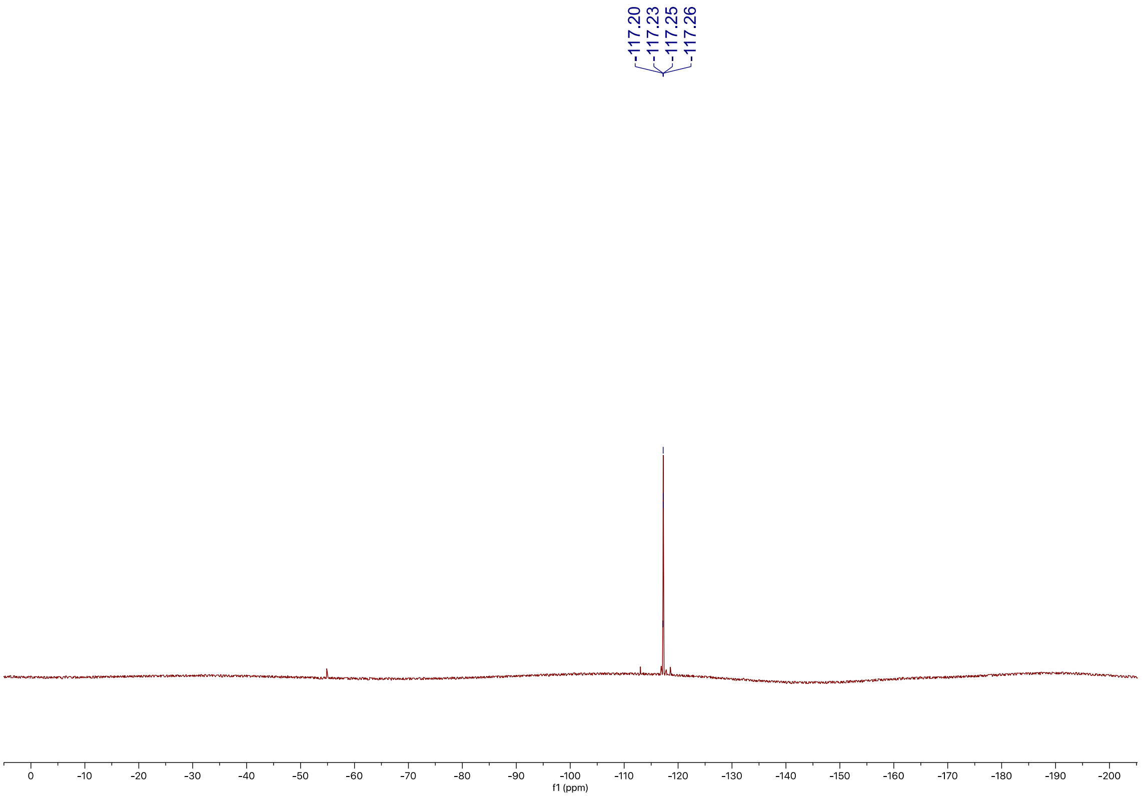

Figure S41. ^{19}F NMR spectrum of (*E*)-2-styrylchromone **11i** (282 MHz, $CDCl_3$).

Compound 11j

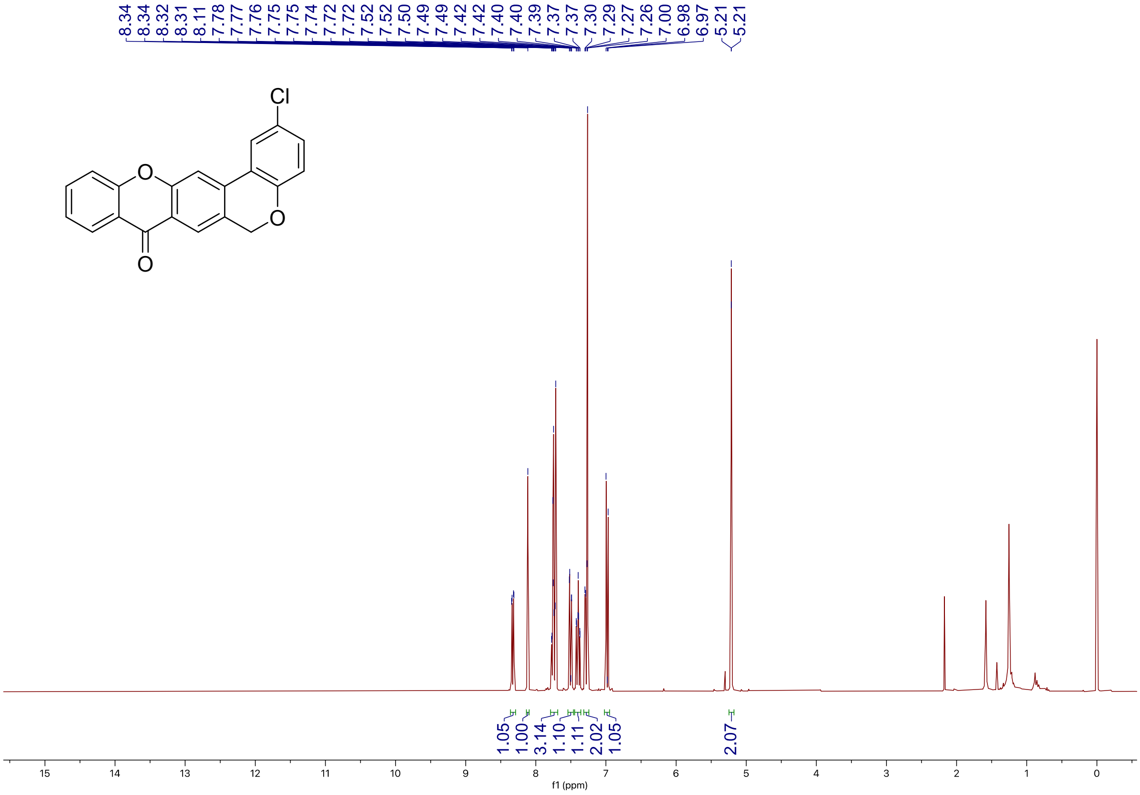

Figure S42. ^{1}H NMR spectrum of chromeno[3,4-*b*]xanthone **11j** (300 MHz, $CDCl_3$).

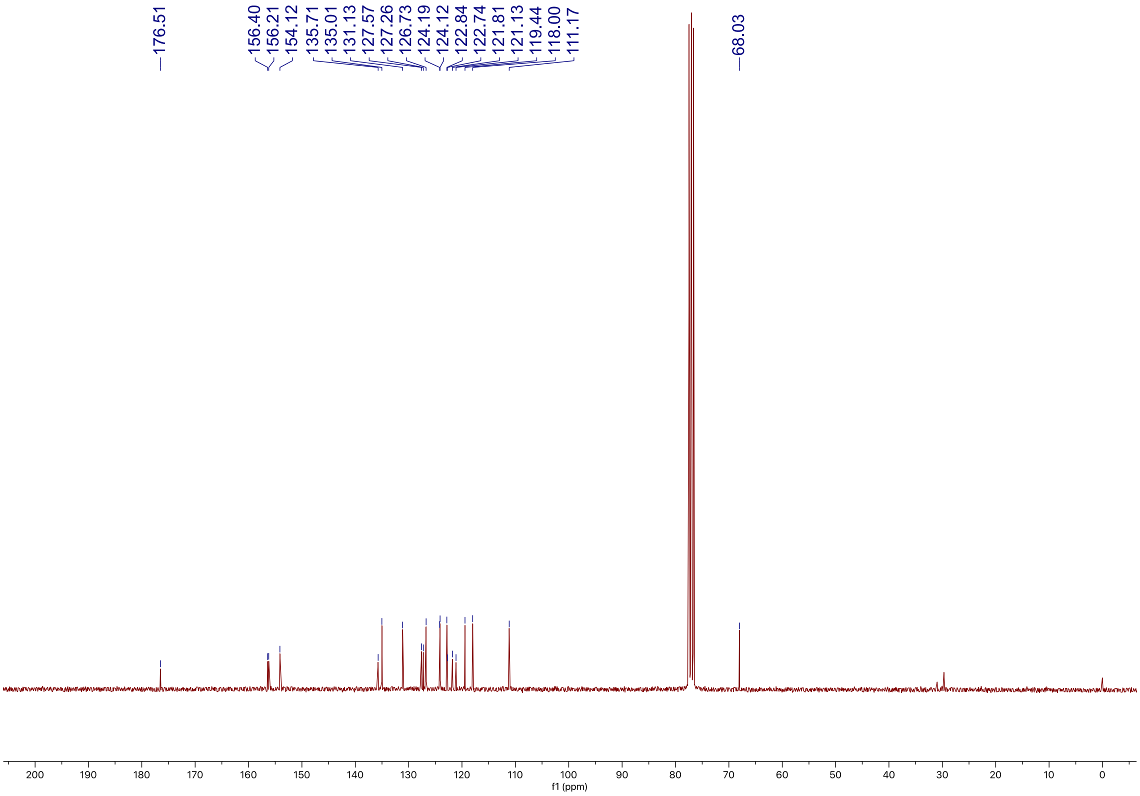

Figure S43. ^{13}C NMR spectrum of chromeno[3,4-*b*]xanthone **11j** (75 MHz, $CDCl_3$).

Compound 11k

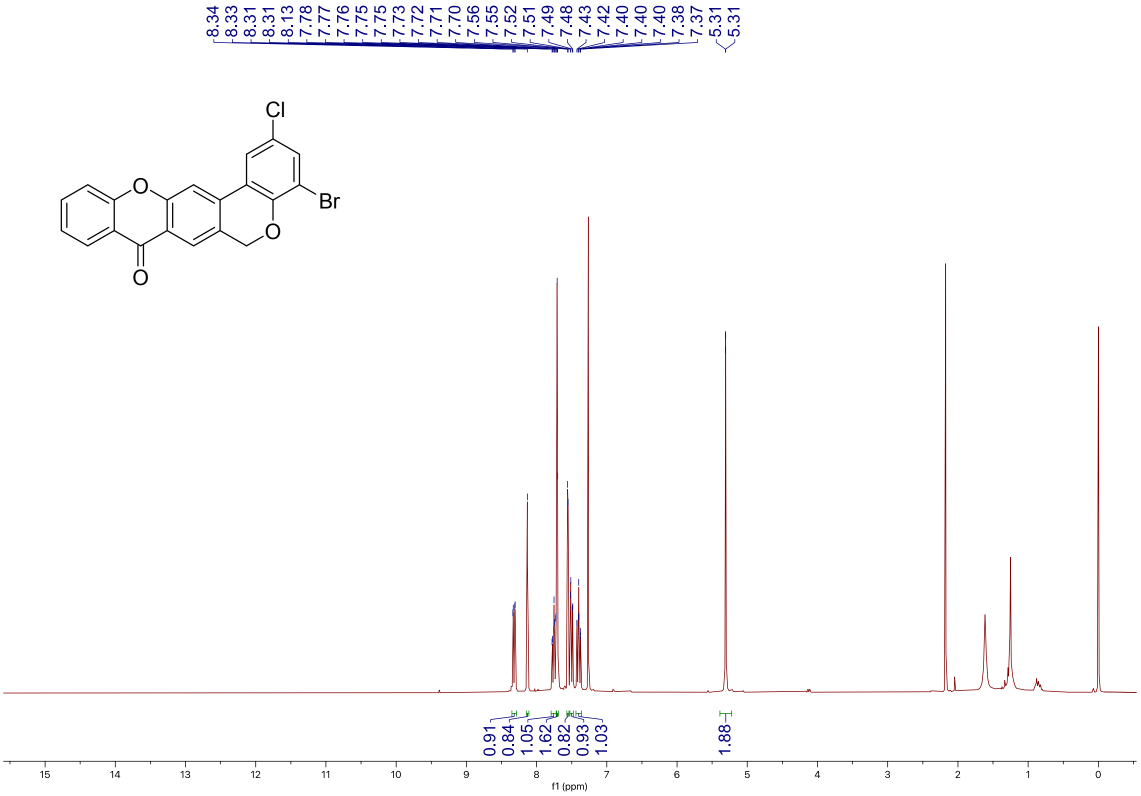

Figure S44. ^{1}H NMR spectrum of chromeno[3,4-*b*]xanthone **11k** (300 MHz, $CDCl_3$).

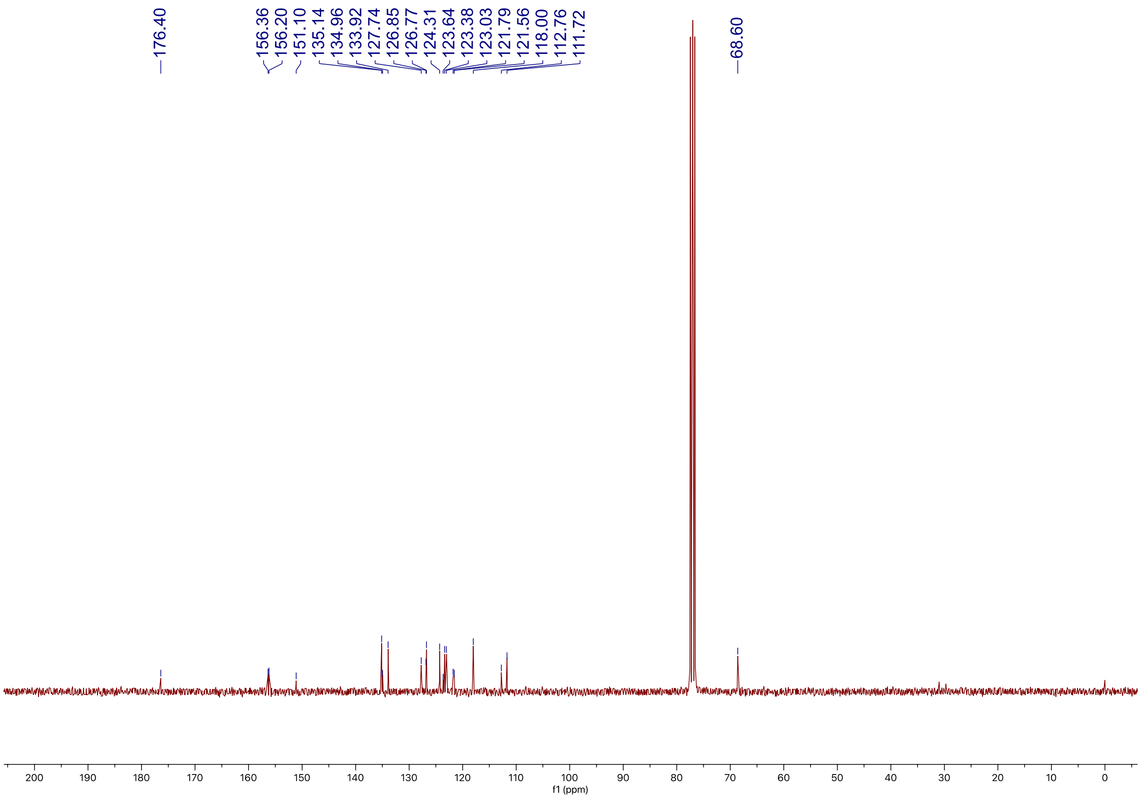

Figure S45. ^{13}C NMR spectrum of chromeno[3,4-*b*]xanthone **11k** (75 MHz, $CDCl_3$).

Compound 11o

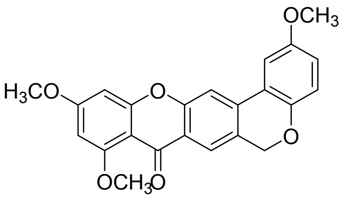

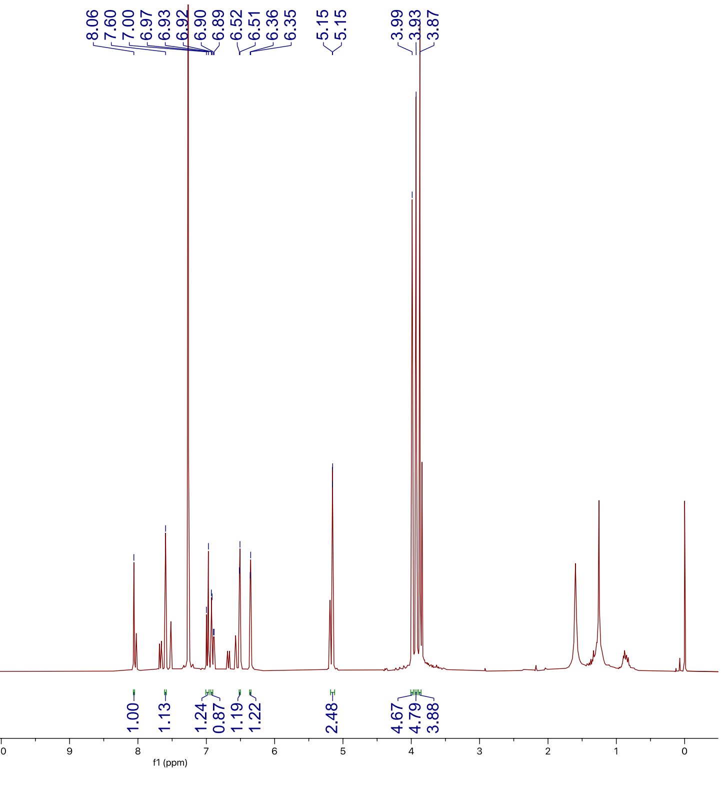

Figure S46. ^{1}H NMR spectrum of chromeno[3,4-*b*]xanthone **11o** (300 MHz, $CDCl_3$).

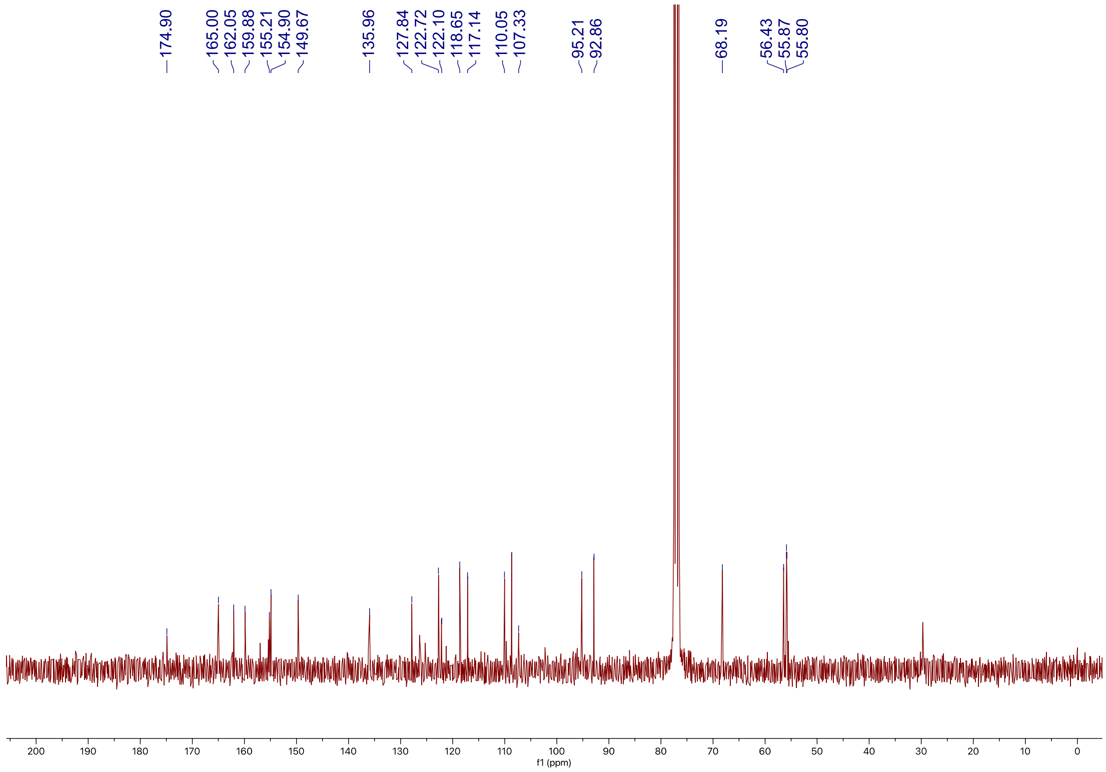

Figure S47. ^{13}C NMR spectrum of chromeno[3,4-*b*]xanthone **11o** (75 MHz, $CDCl_3$).

Compound 11p

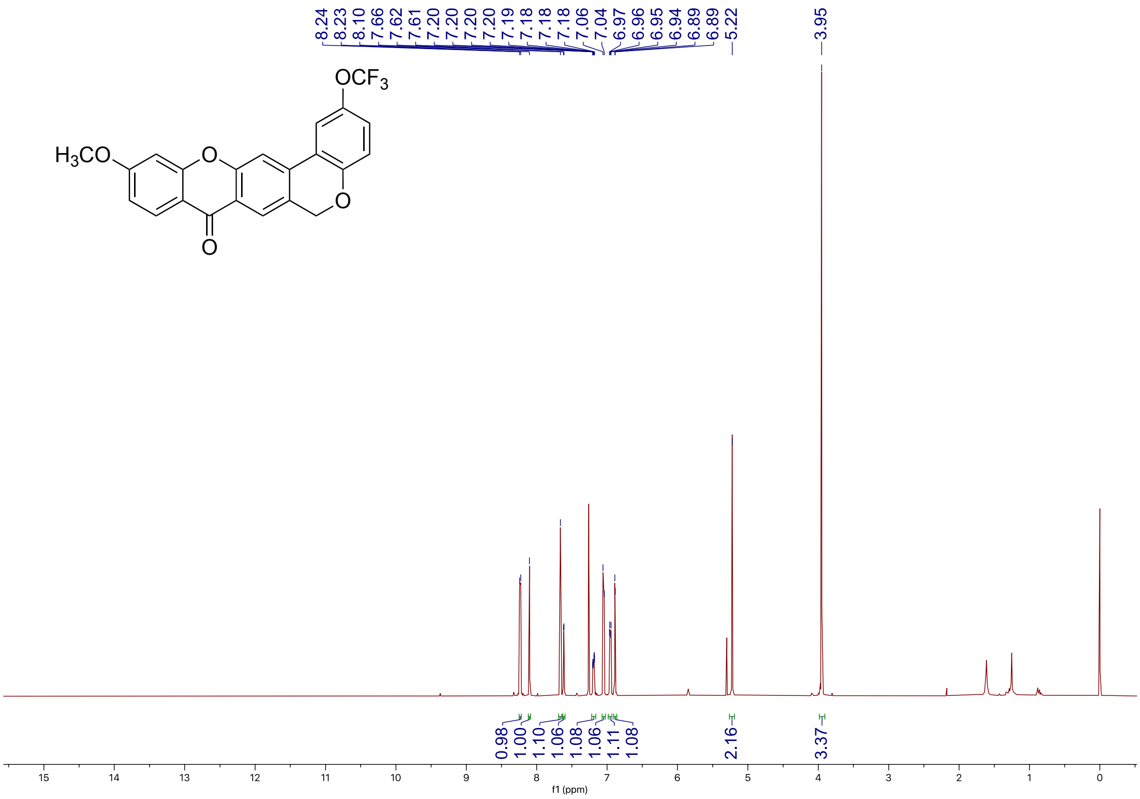

Figure S48. ^{1}H NMR spectrum of chromeno[3,4-*b*]xanthone **11p** (500 MHz, $CDCl_3$).

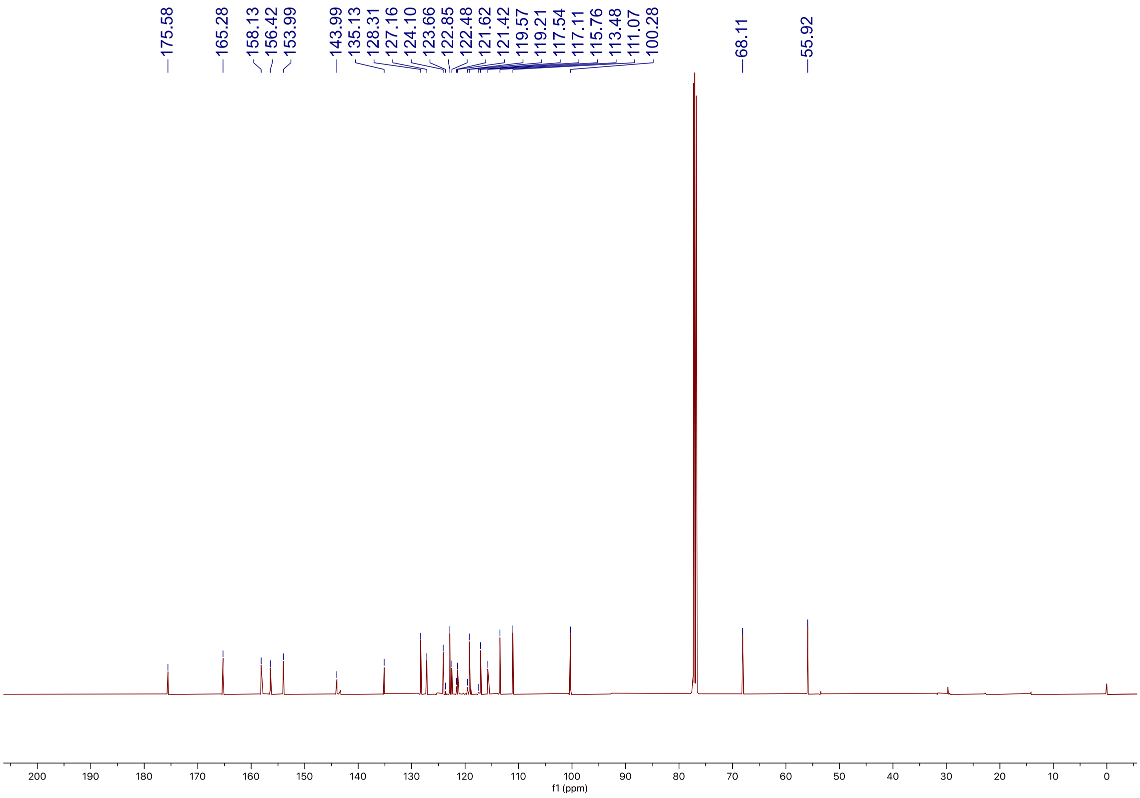

Figure S49. ^{13}C NMR spectrum of chromeno[3,4-*b*]xanthone **11p** (125 MHz, $CDCl_3$).

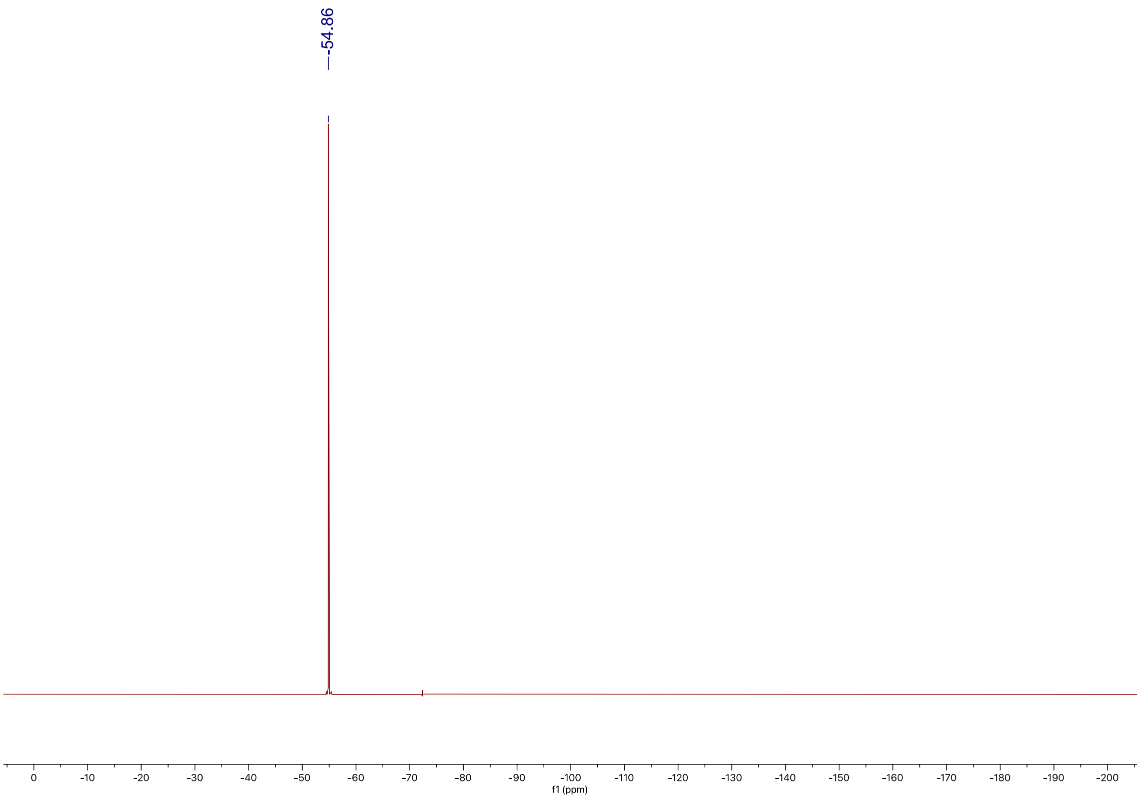

Figure S50. ^{19}F NMR spectrum of (*E*)-2-styrylchromone **11p** (282 MHz, $CDCl_3$).

Compound 11q

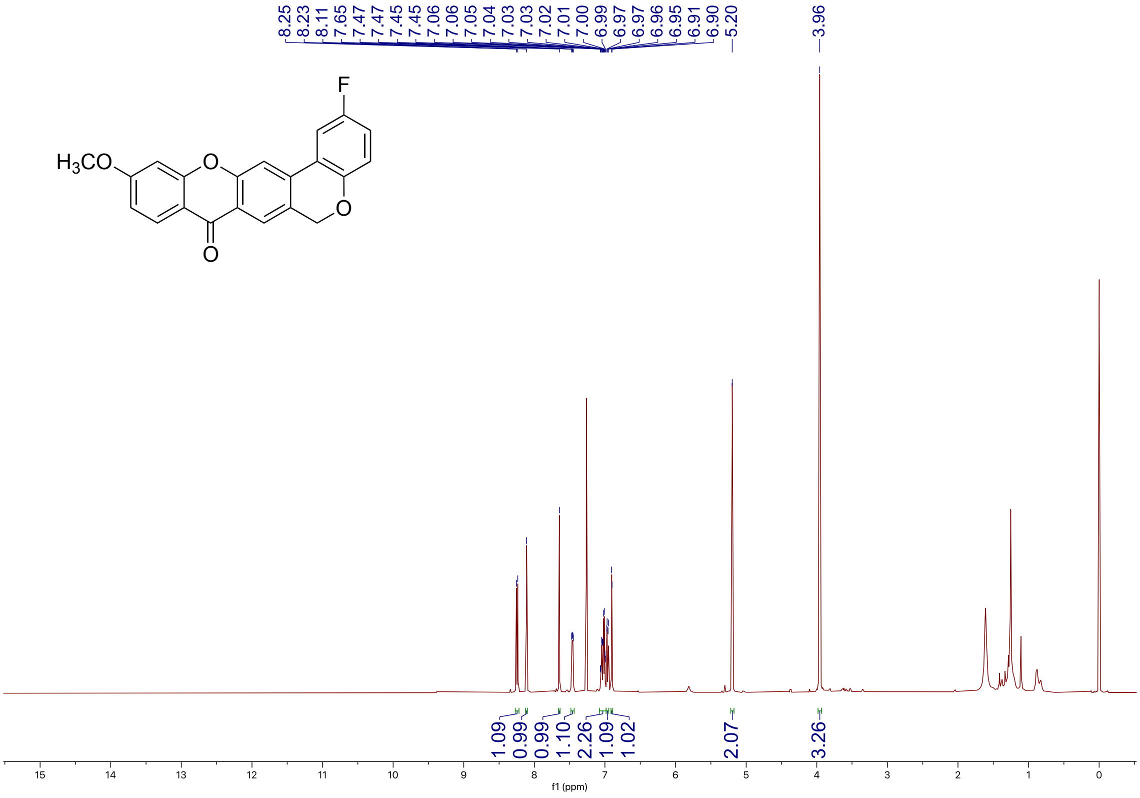

Figure S51. ^{1}H NMR spectrum of chromeno[3,4-*b*]xanthone **11q** (500 MHz, $CDCl_3$).

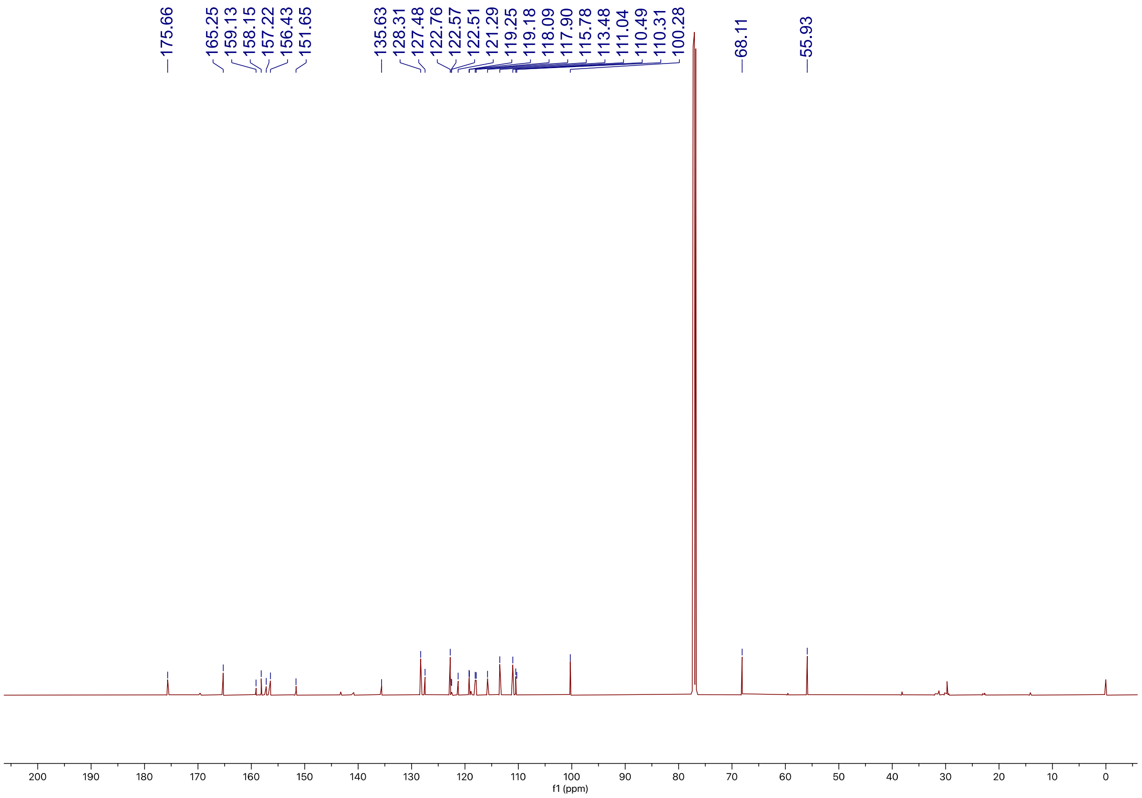

Figure S52. ^{13}C NMR spectrum of chromeno[3,4-*b*]xanthone **11q** (125 MHz, $CDCl_3$).

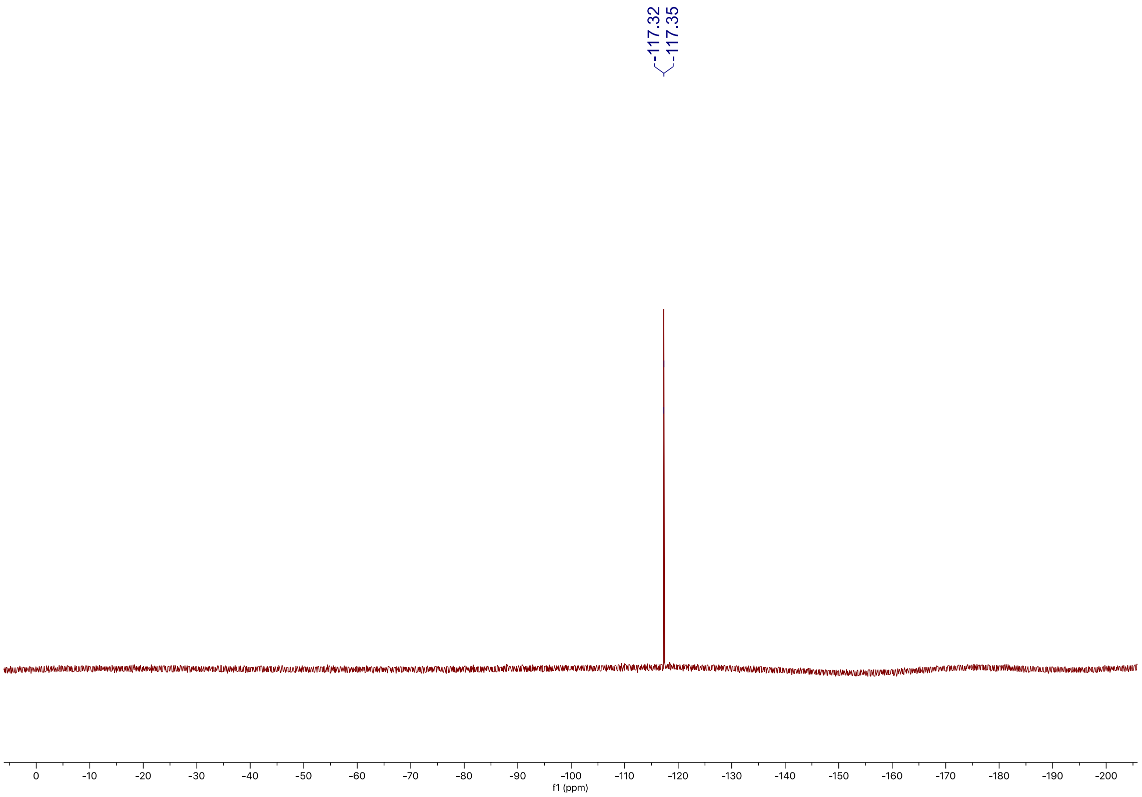

Figure S53. ^{19}F NMR spectrum of (*E*)-2-styrylchromone **11q** (282 MHz, $CDCl_3$).

Compound 11r

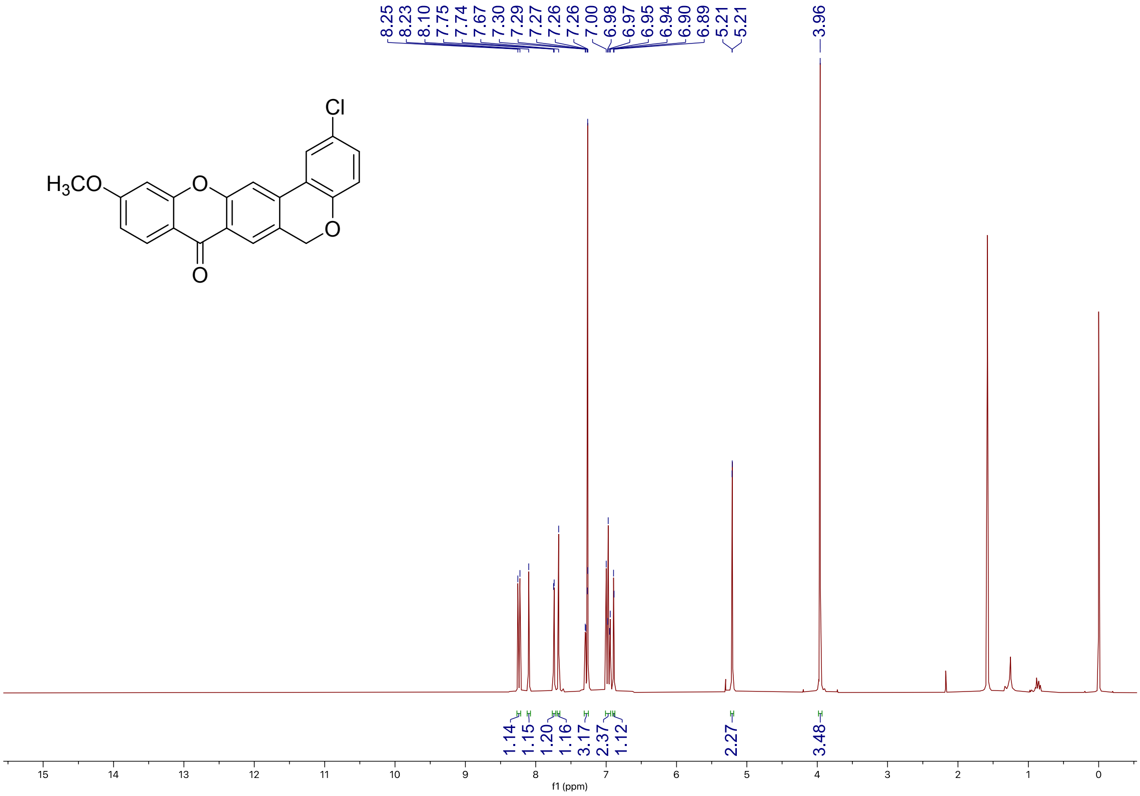

Figure S54. ^{1}H NMR spectrum of chromeno[3,4-*b*]xanthone **11r** (300 MHz, $CDCl_3$).

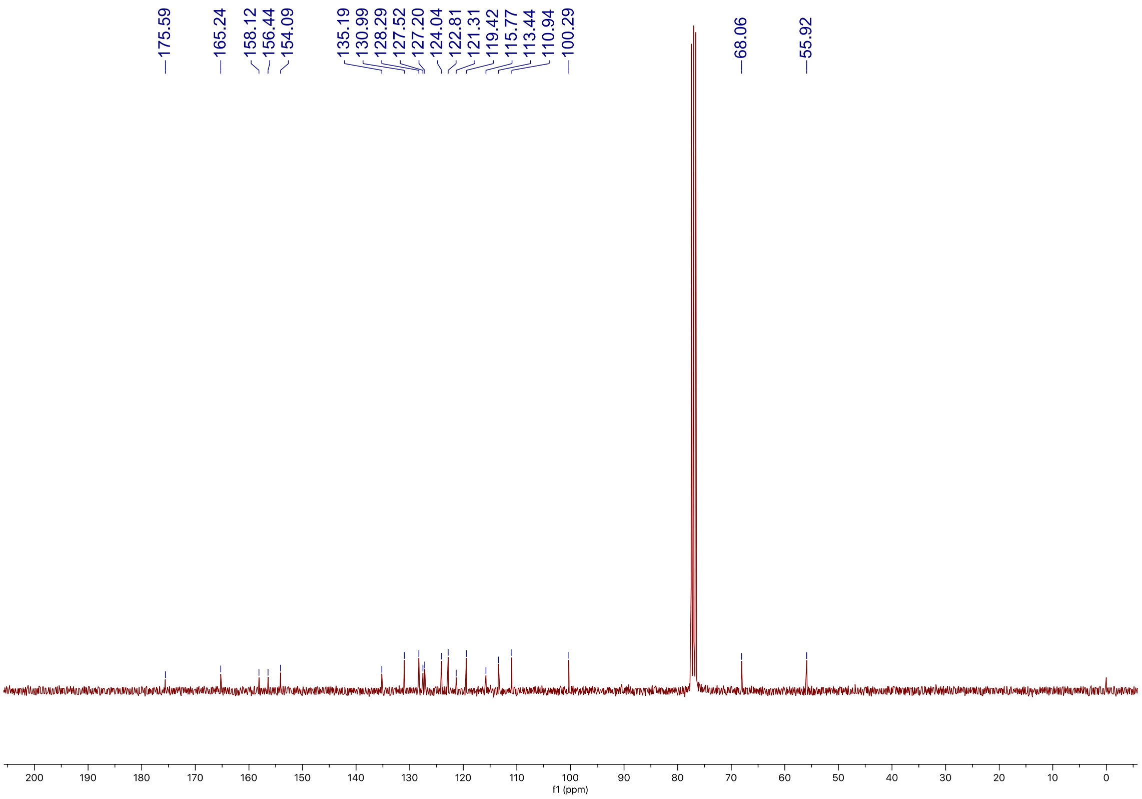

Figure S55. ^{13}C NMR spectrum of chromeno[3,4-*b*]xanthone **11r** (75 MHz, $CDCl_3$).

Compound 11s

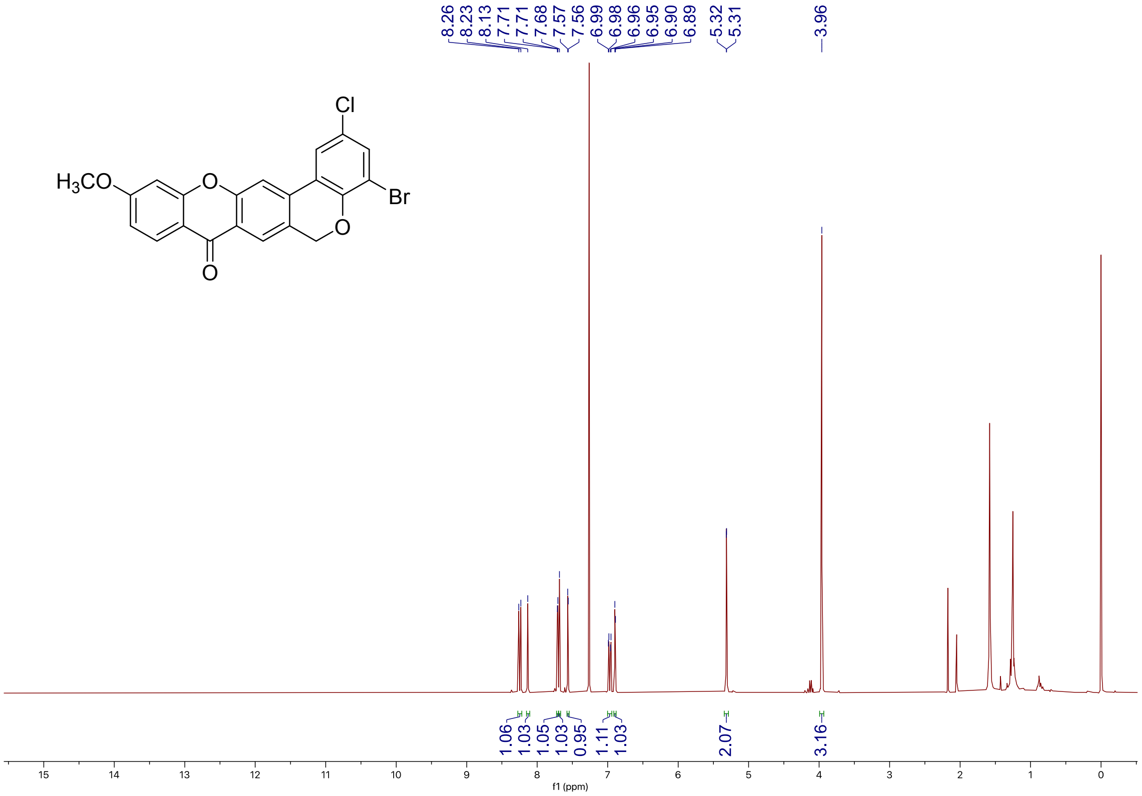

Figure S56. ^{1}H NMR spectrum of chromeno[3,4-*b*]xanthone **11s** (300 MHz, $CDCl_3$).

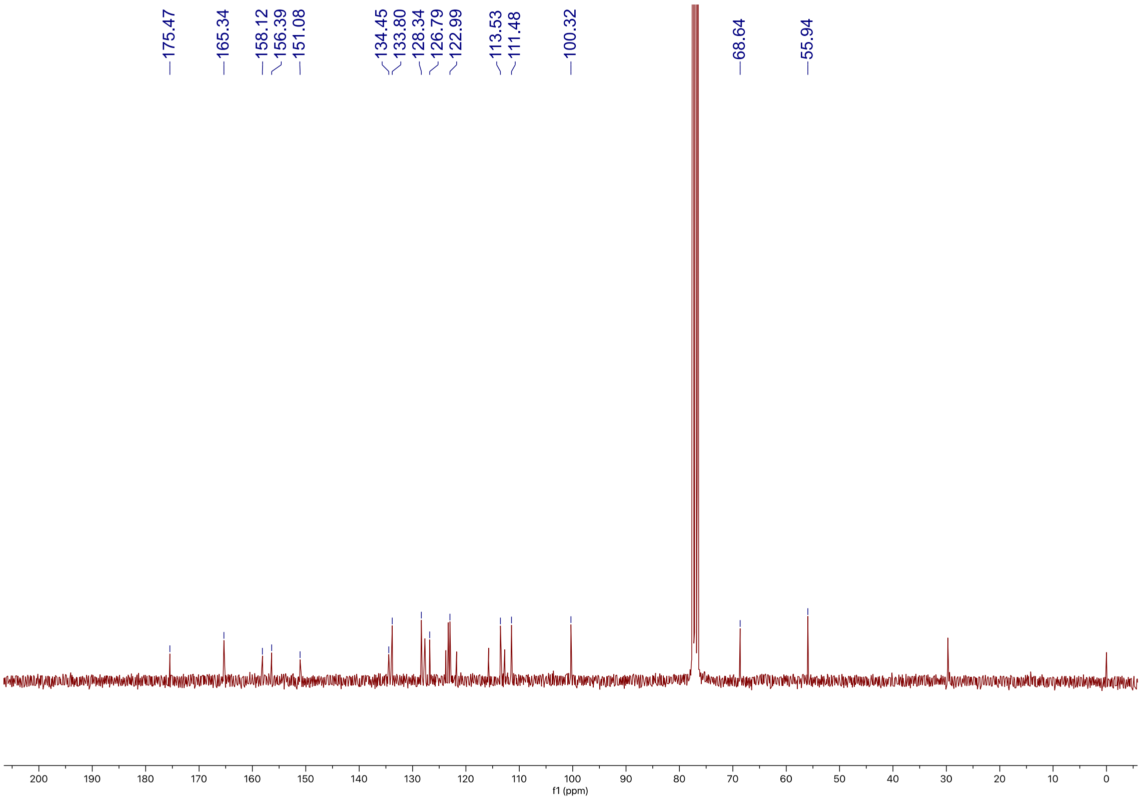

Figure S57. ^{13}C NMR spectrum of chromeno[3,4-*b*]xanthone **11s** (75 MHz, $CDCl_3$).

Compound 12a

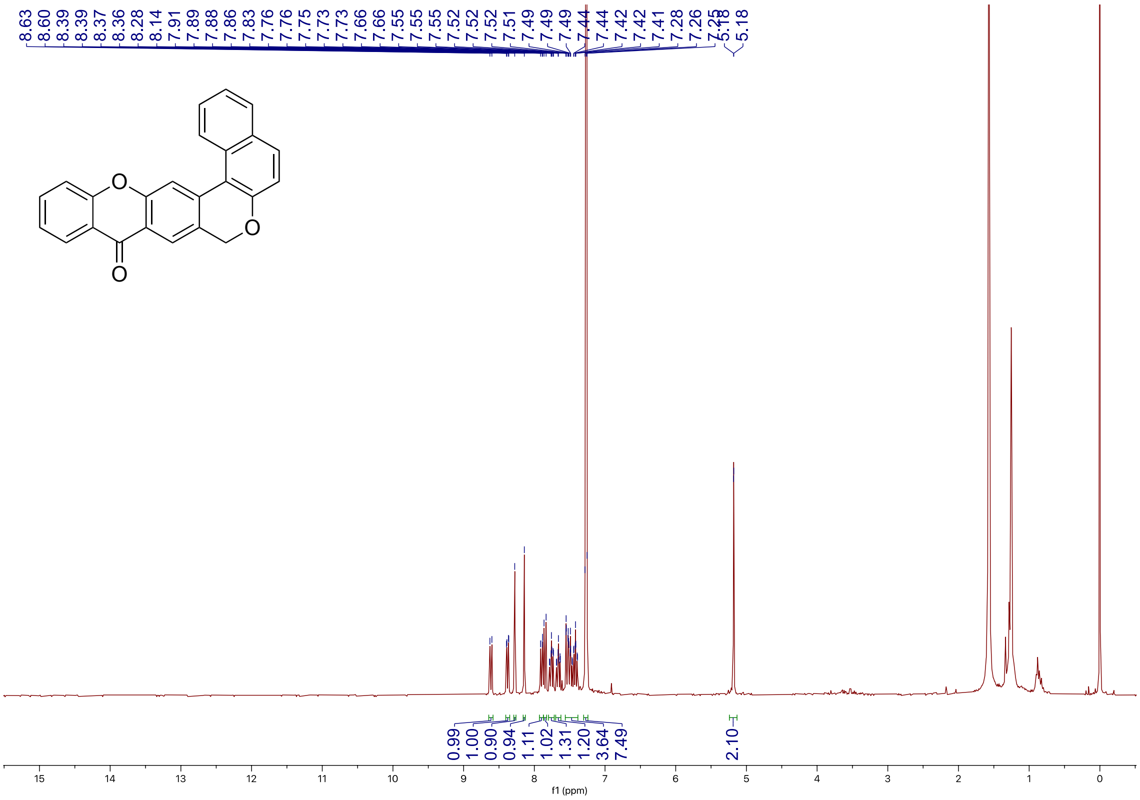

Figure S58. ^{1}H NMR spectrum of chromeno[3,4-*b*]xanthone **12a** (300 MHz, $CDCl_3$).

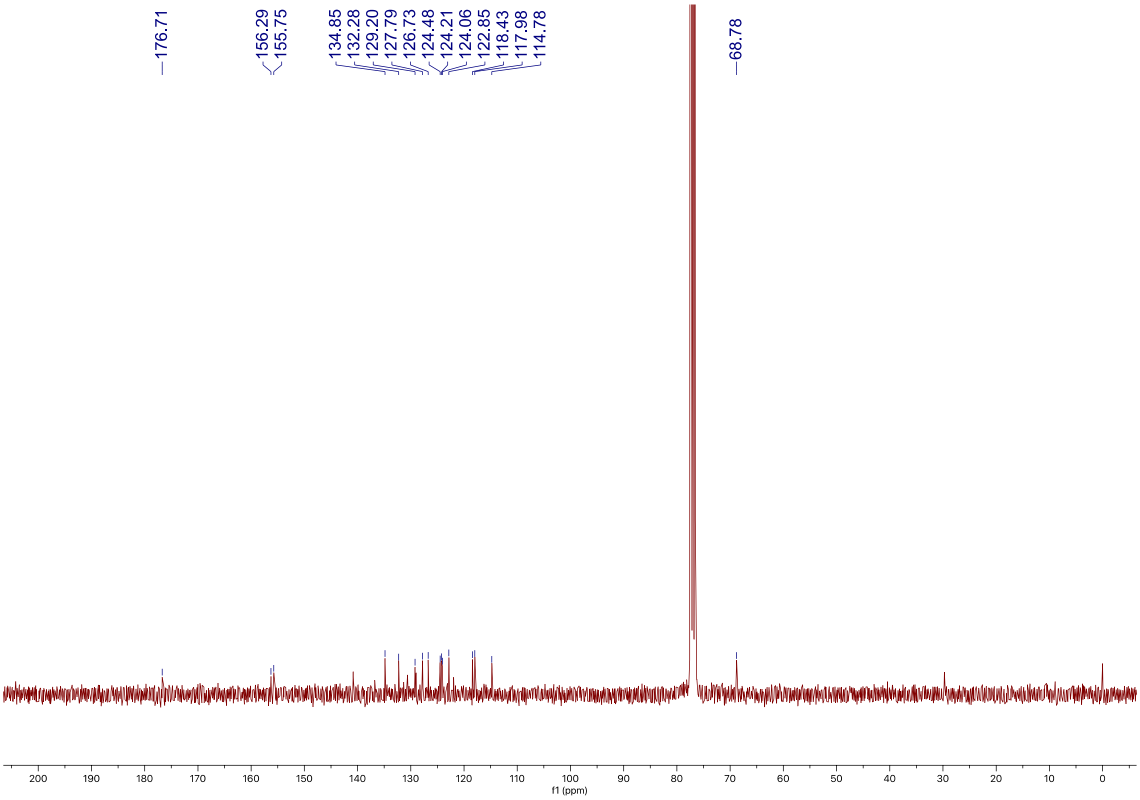

Figure S59. ^{13}C NMR spectrum of chromeno[3,4-*b*]xanthone **12a** (75 MHz, $CDCl_3$).

Compound 12b

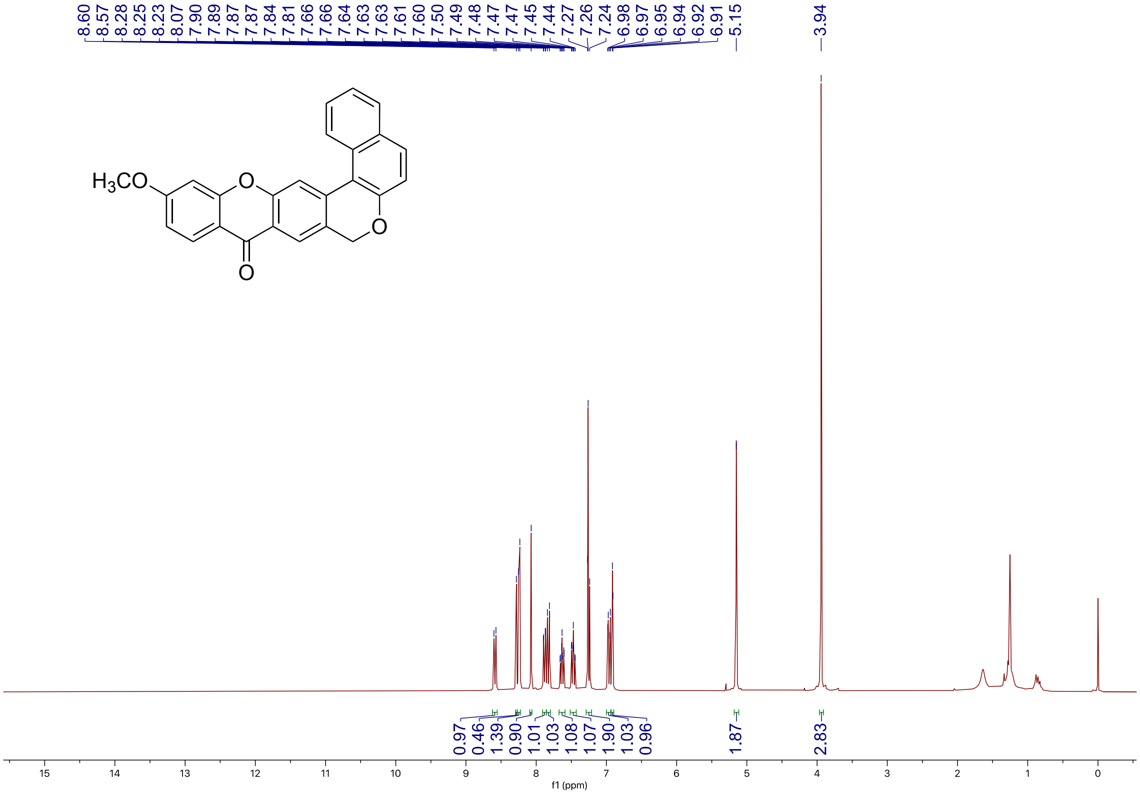

Figure S60. ^{1}H NMR spectrum of chromeno[3,4-*b*]xanthone **12b** (300 MHz, $CDCl_3$).

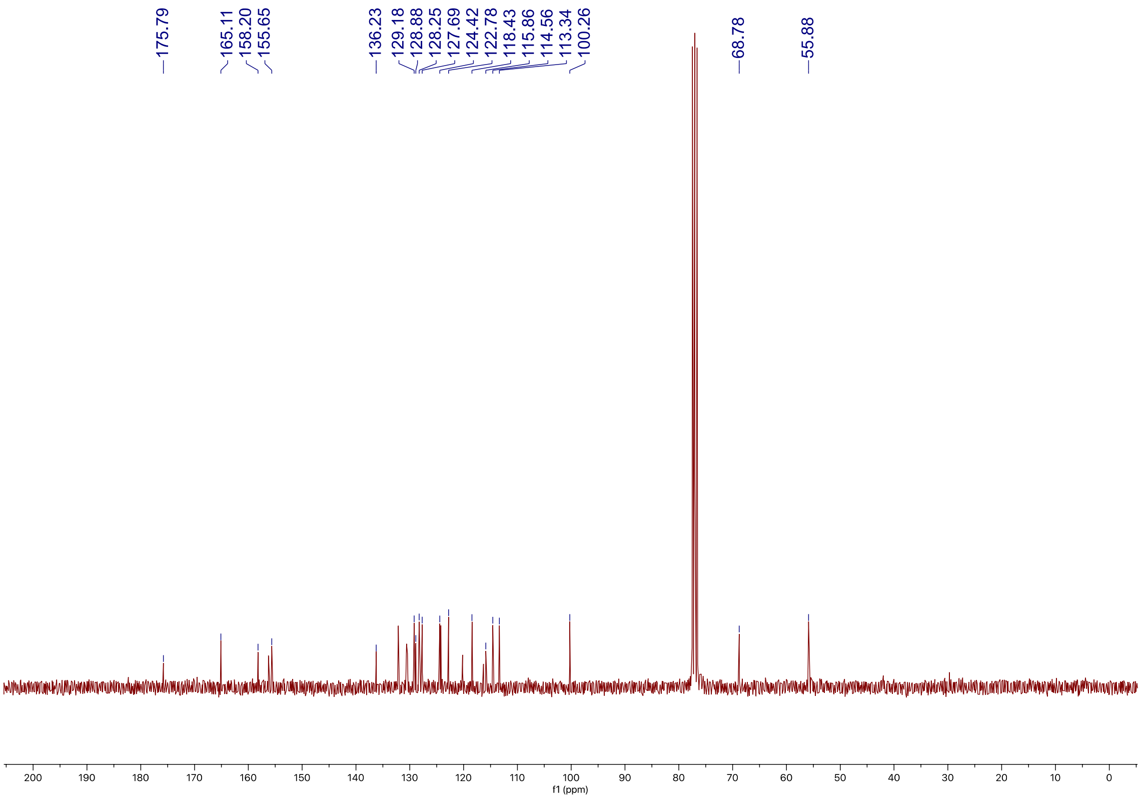

Figure S61. ^{13}C NMR spectrum of chromeno[3,4-*b*]xanthone **12b** (75 MHz, $CDCl_3$).

References

1. Shaw, A. Y.; Chang, C.-Y.; Liau, H.-H.; Lu, P.-J.; Chen, H.-L.; Yang, C.-N.; Li, H.-Y., Synthesis of 2-styrylchromones as a novel class of antiproliferative agents targeting carcinoma cells. *Eur. J. Med. Chem.* **2009,** *44*, 2552-2562.
2. Liu, J.; Li, Z.; Tong, P.; Xie, Z.; Zhang, Y.; Li, Y., TMSI-Promoted Vinylogous Michael Addition of Siloxyfuran to 2-Substituted Chromones: A General Approach for the Total Synthesis of Chromanone Lactone Natural Products. *J. Org. Chem.* **2015,** *80*, 1632-1643.
3. Albuquerque, H. M. T.; Santos, C. M. M.; Cavaleiro, J. A. S.; Silva, A. M. S., First intramolecular Diels–Alder reactions using chromone derivatives: synthesis of chromeno[3,4-b]xanthones and 2-(benzo[c]chromenyl)chromones. *New J. Chem.* **2018,** *42*, 4251-4260.
4. Muthusamy, S.; Gangadurai, C., “On water” cascade synthesis of benzopyranopyrazoles and their macrocycles. *Tetrahedron Lett.* **2018,** *59*, 1501-1505.
5. Qin, L.; Cheng, X.; Wang, S.; Gong, G.; Su, H.; Huang, H.; Chen, T.; Damdinjav, D.; Dorjsuren, B.; Li, Z.; Qiu, Z.; Bian, J., Discovery of Novel Aminobutanoic Acid-Based ASCT2 Inhibitors for the Treatment of Non-Small-Cell Lung Cancer. *J. Med. Chem.* **2024,** *67*, 988-1007.
6. Arepalli, S. K.; Park, B.; Jung, J.-K.; Lee, K.; Lee, H., A facile one-pot regioselective synthesis of functionalized novel benzo[f]chromeno[4,3-b][1,7]naphthyridines and benzo[f][1,7]naphthyridines via an imino Diels-Alder reaction. *Tetrahedron Lett.* **2017,** *58*, 449-454.
7. Fang, W.; Wei, Y.; Tang, X.-Y.; Shi, M., Gold(I)-Catalyzed Cycloisomerization of ortho-(Propargyloxy)arenemethylenecyclopropanes Controlled by Adjacent Substituents at Aromatic Rings. *Chem. Eur. J.* **2017,** *23*, 6845-6852.
8. Hoplamaz, E.; Keskin, S.; Balci, M., Regioselective Synthesis of Benzo[h][1,6]-naphthyridines and Chromenopyrazinones through Alkyne Cyclization. *Eur. J. Org. Chem.* **2017**, 1489-1497.
9. Biju, A. T.; Wurz, N. E.; Glorius, F., N-Heterocyclic Carbene-Catalyzed Cascade Reaction Involving the Hydroacylation of Unactivated Alkynes. *J. Am. Chem. Soc.* **2010,** *132*, 5970-5971.
